# Phytochemical Profile and α‐Amylase Inhibitory Activity of Pomegranate Peel Extract: An In Silico and In Vitro Investigation

**DOI:** 10.1002/fsn3.71191

**Published:** 2025-11-11

**Authors:** Shankar Thapa, Deepti Pandey, Mahalakshmi Suresha Biradar, Monica Arora, Shithin Ann Varghese, Akila Elias, Aleesha Mujeeb Shaheen, Somashekhar M. Metri, Shaik Sadik, Sreeharsha Nagaraja, Sharmila Gote, Bipindra Pandey

**Affiliations:** ^1^ Department of Pharmacy Universal College of Medical Sciences Bhairahawa Nepal; ^2^ Department of Pharmaceutical Chemistry Al‐Ameen College of Pharmacy Bengaluru India; ^3^ Faculty of Health Sciences Male' Maldives; ^4^ Department of Pharmaceutics Al‐Ameen College of Pharmacy Bangaluru India; ^5^ Department of Pharmacognosy Al‐Ameen College of Pharmacy Bangalore India; ^6^ Department of Pharmaceutical Chemistry BLDEA's Shri Sanganabasava Mahaswamiji College of Pharmacy and Research Centre Vijayapur India; ^7^ Department of Pharmacology East Point College of Pharmacy Bengaluru India; ^8^ Department of Pharmaceutical Sciences, College of Clinical Pharmacy King Faisal University Al‐Ahsa Saudi Arabia; ^9^ Department of Pharmaceutical Chemistry Nargund College of Pharmacy Bangalore India; ^10^ Department of Pharmacy Madan Bhandari Academy of Health Sciences Hetauda Nepal

**Keywords:** α‐amylase, docking, MD simulation, phytochemical profiling, pomegranate peel

## Abstract

Diabetes is among the top ten causes of mortality and morbidity. Diabetes has been increasing faster in low‐ and middle‐income nations than it has been rising in high‐income countries. This study explores the phytochemical profiling and in vitro α‐amylase inhibitory activity of the methanolic extract of Nepalese‐origin pomegranate peels. Phytochemical analysis using FT‐IR and LC–MS identified key bioactive compounds, including ellagic acid, punicalagin, punicalin, gallic acid, and ellagic acid‐O‐xylopyranoside, contributing to the observed enzymatic inhibition. Molecular docking studies revealed strong binding affinities of punicalagin (−11.6 kcal/mol) and ellagic acid‐O‐xylopyranoside (−10.6 kcal/mol) to α‐amylase, suggesting potential antidiabetic properties. MD simulations confirmed the stability of these interactions over a 100 ns timeframe, reinforcing their role in enzyme inhibition. The in vitro α‐amylase inhibition assay demonstrated comparable activity of pomegranate peel extract (IC_50_ = 137.74 μg/mL) to reference drug acarbose (IC_50_ = 222.19 μg/mL). These findings provide molecular insights into the bioactivity of pomegranate peel extracts, highlighting their potential in diabetes management.

## Graphical abstract

The graphical abstract illustrates a systematic workflow that begins with the extraction of bioactive compounds from pomegranate. LC–MS analysis identifies punicalagin, and molecular docking evaluates the binding of punicalagin to α‐amylase, followed by in vitro validation of its inhibitory activity. Molecular dynamics (MD) simulation further assesses the stability of the complex, and MM/GBSA energy analysis quantifies the binding affinity. The study highlights the antidiabetic potential of pomegranate‐derived compounds using an integrative experimental and computational approach.

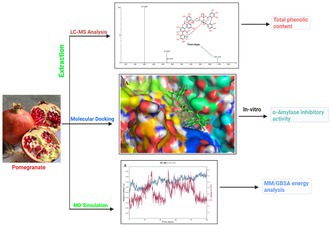

**Abbreviations**
FT‐IR: Fourier Transform‐Infrared Spectroscopy
GAE: gallic acid equivalents
LC–MS: liquid chromatography‐mass spectroscopy
MD: molecular dynamics
MM/GBSA: molecular mechanics generalized born surface area
MolSASA: molecular solvent‐accessible surface area
NMR: nuclear magnetic resonance
PPE: pomegranate peel extract
PSA: polar surface area
QqQ: triple quadrupole
rGyr: radius of gyration
RMSD: root mean square deviation
RMSF: root mean square fluctuation
RT: retention time
SASA: polvent‐accessible surface area

## 1 Introduction

In recent years, the incidence of type 2 diabetes mellitus has been steadily increasing, emerging as a significant global health concern. This growing epidemic poses serious challenges not only to public health systems but also to economic development worldwide (Khan et al. 2020). By 2045, it is projected that 1 in 8 adults, approximately 783 million people, will be living with diabetes, representing a 46% increase from current figures. More than 90% of these cases will be type 2 diabetes, primarily driven by a combination of socio‐economic, demographic, environmental, and genetic factors. Major contributors include rapid urbanization, an aging population, decreased physical activity, and rising rates of overweight and obesity, all of which are intensifying the global diabetes burden (IDF 2021). Compared to high‐income countries, prevalence has been increasing more quickly in low‐ and middle‐income countries (Hossain et al. 2024). One of the key therapeutic targets in diabetes management is α‐amylase, an enzyme responsible for carbohydrate digestion, whose inhibition can effectively regulate postprandial blood glucose levels (Ma et al. 2024). Acarbose, miglitol, and voglibose are α‐amylase/β‐glucosidase inhibitors for managing type 2 diabetes, but prolonged use can cause gastrointestinal discomfort, severe hypoglycemia, and weight gain (Cisneros‐Yupanqui et al. 2023; Kashtoh and Baek 2023). Given the increasing global prevalence of diabetes and the search for safer, natural alternatives to synthetic α‐amylase inhibitors, exploring the bioactive potential of natural products is of high relevance.

Pomegranate ( *Punica granatum* L.) is widely recognized for its rich phytochemical composition, particularly in its peel, which contains abundant polyphenols, flavonoids, and tannins with significant pharmacological activities. More than 40 phenolic compounds have been identified in the pomegranate peel and other parts of the fruit (Saparbekova et al. 2023; Singh et al. 2023). Research indicates that hydrolysed tannins like punicalagin and punicalin may give the fruit its anticancer, anti‐inflammatory, anti‐diabetic, anti‐obesity, and antioxidant properties (Siddiqui et al. 2024; Teniente et al. 2023). The pomegranate peel additionally has a potent anti‐diabetic effect with improvements in insulin sensitivity and a decrease in oxidative stress in diabetic mouse models (Rak‐Pasikowska et al. 2024). Among these, the potential of pomegranate peel extract (PPE) in managing diabetes has gained increasing attention (Mo et al. 2022).

A phytochemical‐based approach allows for the identification of key bioactive phytochemicals, while molecular docking and molecular dynamics (MD) simulations can provide insights into their binding mechanisms at a molecular level (Nyambo et al. 2024). Although several studies have reported the anti‐diabetic potential of pomegranate extracts (Aslam et al. 2024; Gull et al. 2023; More et al. 2024; Royapuram Parthasarathy et al. 2023), no research has been conducted on Nepalese‐origin pomegranate, which may possess distinct phytochemical profiles due to unique climatic and geographical conditions. Additionally, the molecular interactions between pomegranate peel phytochemicals and α‐amylase remain poorly understood, necessitating an in‐depth investigation using in vitro and computational methods. This study integrates phytochemical profiling, in vitro enzyme inhibition assays, and computational analysis to establish a molecular explanation for the observed bioactivity of PPE.

## 2 Materials and Methods

### 2.1 Fruit Collection and Authentication

The pomegranate fruits were bought from the farm in Butwal, Nepal (geographical coordinate 27.6983° N, 83.4653° E). The peels of the fruit were collected and dried for research work. Later, an herbarium was prepared from the fresh fruit peel and sent to the Department of Horticulture and Plant Protection, Pakhlihawa Campus, Rupandehi, Nepal, for identification and certification. Certification was provided by Pushpa Raj Poudel, PhD.

### 2.2 Extraction Method

The pomegranate peels were separated from the arils or seeds, collected, and naturally dried on trays away from sunlight at room temperature. The peel was powdered to obtain particles and passed through an 80‐mesh sieve. 40 g pomegranate peel powder was weighed and extracted in a Soxhlet apparatus using 80% methanol as a solvent. The temperature was maintained at 40°C throughout the extraction. The final extract was dried and stored at 4°C until used for further analysis. The extractive value in percentage was calculated by using the following formula (Equation (1)) and recorded (Fawole et al. 2012).

(1) $$\text{Extractive value}\left(\%\right)=\frac{\text{weight of dried extract}}{\text{weight of plant material}}\times 100$$

### 2.3 Phytochemical Screening

Phytochemical screening of *Punica granatum* peel extract was subjected to qualitative analysis for the identification of various active compounds such as alkaloids, terpenoids, glycosides, saponins, flavonoids, tannins, and carbohydrates (Yadav et al. 2023).

### 2.4 Determination of Total Phenolic Content

Total phenolic content was analyzed using the Folin–Ciocalteu method. In this method, 70 μL of pomegranate peel extract was placed into a 10 mL test tube, and 250 μL of Folin–Ciocalteu's reagent and 750 μL of 1.9 M sodium carbonate were added. The total volume was made up to 5 mL by adding distilled water and mixing for 1 min, and then incubated for 2 h in the dark. Subsequently, the absorbance was measured at 765 nm using a UV–visible spectrophotometer. An appropriate calibration curve was prepared using standard solutions of gallic acid. Results were expressed as gallic acid equivalents (GAE) in mg/g dry solids (Magangana et al. 2021).

### 2.5 Fourier Transform‐Infrared Spectroscopy (FT‐IR) Screening

FT‐IR provides insight into the functional group present in the phytochemical. The Department of Central Instrumental Analysis, Nargund College of Pharmacy, Bengaluru, India, provided the space and facility for spectral analysis. The IR spectra of the methanolic extract of Pomegranate peel were recorded from FT‐IR (Model Shimadzu 8700) in the range of 500–4000 cm^−1^ by the KBr pellet method (Bachari et al. 2024).

### 2.6 Liquid Chromatography‐Mass Spectroscopy (LC–MS) Protocol

An LC–MS experiment was conducted in the advanced analytical instrumentation facility, Honeychem Pharma Research Pvt. Ltd., Bengaluru, India. The system consisted of a liquid chromatographic system UPLC Acquity H class series with separations on a WATERS XBridge (50 × 4.6 mm 3.5 μ), C18 column. The mobile phase was 0.1% formic acid in water (A) and Acetonitrile HPLC grade (B) with a flow rate of 0.5 mL/min. Analysis was performed using the gradient elution method. The pomegranate extract sample was prepared, and both positive and negative analyses were performed on each sample. Triple Quadrupole (QqQ) MS/MS analyzers were used to perform MS analysis. MassLynxV4.1SCN805 software was used to analyze the data, which included retention time (RT) and mass spectra, that were obtained from the mass spectrometer (Yadav et al. 2023). High‐resolution mass spectra and LC retention time were used to identify secondary metabolites in the pomegranate extracts. Moreover, compounds were putatively identified by comparing mass spectra and RT with published articles (Elkahoui et al. 2024; Khan et al. 2017; Peršurić et al. 2020; Setty et al. 2023) and web servers (MassBank, https://massbank.eu/MassBank/Search, Chemdata, https://chemdata.nist.gov/, RefMetaPlant, https://www.biosino.org/RefMetaDB/, IMPPAT 2.0, https://cb.imsc.res.in/imppat/).

### 2.7 Determination of α‐Amylase Inhibitory Activity

An α‐amylase inhibitory assay was conducted to establish the antidiabetic effect of pomegranate peel extract. In a test tube, an equal volume of 0.02 M sodium phosphate buffer and enzyme (20 μL) was taken. The pomegranate peel extract in a concentration range of 20–100 μg/mL was added to the test tube and incubated for 10 min at room temperature, followed by the addition of 200 μL of starch in all test tubes. The reaction was terminated with the addition of 400 μL DNS reagent and placed in a boiling water bath for 5 min, cooled, and diluted with 15 mL of distilled water, and absorbance was measured at 540 nm. Acarbose was used as a positive control. A sample without peel extract was taken as a negative control. A sample without peel extract and enzyme was taken as a blank. The result was expressed as concentrations of the extract that led to the 50% decrease of α‐amylase activity (IC_50_) (Mechchate et al. 2021). The percentage inhibition of α‐amylase activity was calculated according to the following Equation (2).

(2) $$\%\text{inhibition}=\frac{\;\text{control}-\;\text{sample}}{\;\text{control}}\times 100$$

where control is the absorbance of the reaction mixture without extract, and sample is the absorbance of the reaction mixture with extract.

### 2.8 Molecular Explanation

#### 2.8.1 Molecular Docking

LC–MS profiling allowed us to select the ligand molecules from pomegranate peel. A total of 72 phytochemicals were determined based on m/z value and previous literature search. All the phytochemicals were selected for molecular docking. The 3D structure of phytochemicals was downloaded from the PubChem database in SDF format. Some of the phytochemicals were only available in 2D format (Pandey et al. 2024). Thereafter, we converted them into 3D format manually through Marvin Sketch software. The chirality of the phytochemicals was manually checked along with the inbuilt feature of Marvin Sketch software. The energy minimization (FFMM96 force field) and conversion into the pdbqt file format were run in the Ubuntu system, utilizing the OpenBabel software (Gote et al. 2023).

An α‐amylase enzyme is responsible for the breakdown of carbohydrates into glucose. The α‐amylase enzyme was retrieved from the PDB web server (PDBID 3BAJ) (Vorhees and Williams 2006). The protein was selected because it was present in high resolution (2.10 Å), without mutation, and with a good Ramachandran outlier (Figure S1). Furthermore, it also contained acarbose as a native co‐crystal ligand. All the heteroatoms were removed using BIOVIA Discovery Studio Visualizer 2021 (Thapa et al. 2023). The polar hydrogen atom, Kollmann charge, and missing residues were repaired in AutoDock Tools v1.5.7. Finally, the purified protein was saved in a pdbqt file format and used for a docking study. Site‐specific docking was conducted by defining the grid box around the acarbose binding site (Figure S2). The grid dimensions were generated from Discovery Studio Visualizer 2021. Binding site amino acids were covered by applying the center_x = 11.824, center_y = 18.652, center_z = 45.55 dimension. The molecular docking was performed using AutoDock Vina v1.2.0 software on the Linux platform (Trott and Olson 2009). After docking analysis, we reported the top 10 phytochemicals having a binding score < −9.5 kcal/mol. The visualization was conducted in PyMOL v3.1 software and Discovery Studio Visualizer 2021 (Seeliger and De Groot 2010).

#### 2.8.2 Docking Protocol Validation

Docking protocol validation was performed using the redocking approach. The accuracy of the docking protocol was assessed by calculating the root‐mean‐square deviation (RMSD) between the experimentally determined (crystal) pose and the re‐docked pose. An RMSD value below 2.0 Å was considered acceptable, indicating that the docking protocol could reliably reproduce the native binding conformation and was suitable for further virtual screening or docking studies (Sriharsha et al. 2023).

#### 2.8.3 MD Simulation

MD simulation was performed to validate the ligand‐receptor stability. We ran an MD simulation with Desmond software for 100 ns. To prepare the ligand‐protein complex (punicalagin‐3BAJ), we used the Protein Preparation Wizard from Schrödinger's Maestro suite. This step made sure we added any missing hydrogen atoms and set the right protonation states for physiological pH. We then solvated the system in an explicit TIP3P water model, all contained within an orthorhombic box that kept a 10 Å buffer distance. To balance things out, we added counterions (Na^+^ and Cl^−^) to neutralize the system, followed by a round of energy minimization. After that, we equilibrated the system using the NPT ensemble at 300 K and 1 atm pressure. The simulation itself was carried out with the OPLS4 force field, and we analyzed the trajectory data for metrics like root mean square deviation (RMSD), root mean square fluctuation (RMSF), radius of gyration (rGyr), solvent‐accessible surface area (SASA), and hydrogen bonding interactions (Thapa et al. 2024).

### 2.9 Molecular Mechanics Generalized Born Surface Area (MM/GBSA) Calculation

The last 10 ns snapshot of the simulation trajectory was used for the binding free‐energy (Δ*G* bind) analysis. The punicalagin showed the strongest binding affinity. Therefore, it is selected for MM/GBSA energy calculation. The binding free energy of the punicalagin‐3BAJ docked complex was calculated using the prime module of Maestro (Equation (3)) (Jawarkar et al. 2022).

(3) ∆G=Ebond+Eel+Evdw+Gpolar+Gnpolar−TS

where “Ebond, Eel, and EvdW correspond to the standard MM bonded, electrostatic, and vdW energy terms. Gpolar and Gnpolar are the polar and non‐polar contributions to the solvation free energy, respectively. The final term is the temperature T multiplied by the entropy S, estimated from a normal‐mode analysis or quasi‐harmonic approximation approach” (Taylor and Ho 2023; Thapa et al. 2025).

### 2.10 Statistical Analysis

The in vitro experiment was conducted in triplets, and the data were presented as mean ± SD. Statistical analysis between the control and treatment groups was performed using Microsoft Excel (Office 2016).

## 3 Results

### 3.1 Extractive Value

The extract was dried, and then the percentage yield of plant extract was calculated, which was found to be 49.87%.

### 3.2 Phytochemical Screening

In the present study, phytochemical screening of the methanolic extract detected the presence of alkaloids, phenols, tannins, terpenoids, carbohydrates, saponins, flavonoids, reducing sugars, and the absence of glycosides (Table 1).

**TABLE 1:** Results of phytochemical screening of methanolic extract of pomegranate peel.

SN	Test		Result
1.	Alkaloids	Mayer's test	+
		Wagner's test	+
		Hager's test	−
2.	Glycosides	Brontrager's test	−
3.	Carbohydrates	Molish's test	+
4.	Reducing sugar	Benedicts test	+
5.	Tannins	Fecl_3_ test	+
6.	Phenol	FeCl_3_ test	+
7.	Terpenoids	Salkowski test	+
8.	Flavonoids	FeCl_3_ test	+
9.	Saponin	Froth test	+

*Note:* In the above table, (+) indicates the presence of the respective class of compounds and (−) indicates the absence of the respective class of compounds.

### 3.3 Total Phenolic Content

**FIGURE 1:**
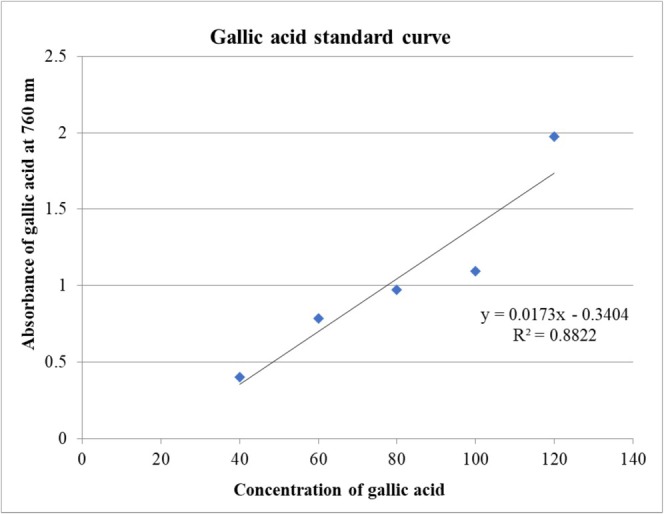
Calibration curve of gallic acid.

In this study, the total phenolic content (TPC) was obtained as mg gallic acid equivalents per gram of dried sample (mg QE/g). The equation obtained from the graph (*y* =0.017*x*‐0.340, *R* ^2^ = 0.882) determined the total phenolic content (mg of gallic acid equivalent/g dry extract) (Figure 1). The value ranges from 29.64 ± 0.97 to 49.54 ± 0.74 mg of gallic acid equivalent/g dry extract. Among them, the highest phenolic content (49.54 ± 0.74) was found at a concentration of 120 μg/mL (Table 2).

**TABLE 2:** Mean absorbance observed by gallic acid and methanolic extract.

Concentration (μg/mL)	Mean absorbance (nm) by methanolic extract	Mean absorbance (nm) by gallic acid	Total phenolic content (GAE) ± SD
40	0.16 ± 0.01	0.39 ± 0.01	29.64 ± 0.97
60	0.23 ± 0.01	0.78 ± 0.06	34.09 ± 0.74
80	0.35 ± 0.03	0.97 ± 0.04	41.15 ± 1.76
100	0.43 ± 0.01	1.09 ± 0.09	45.70 ± 1.12
120	0.50 ± 0.01	1.97 ± 0.08	49.54 ± 0.74

### 3.4 FT‐IR Spectrum Analysis

**FIGURE 2:**
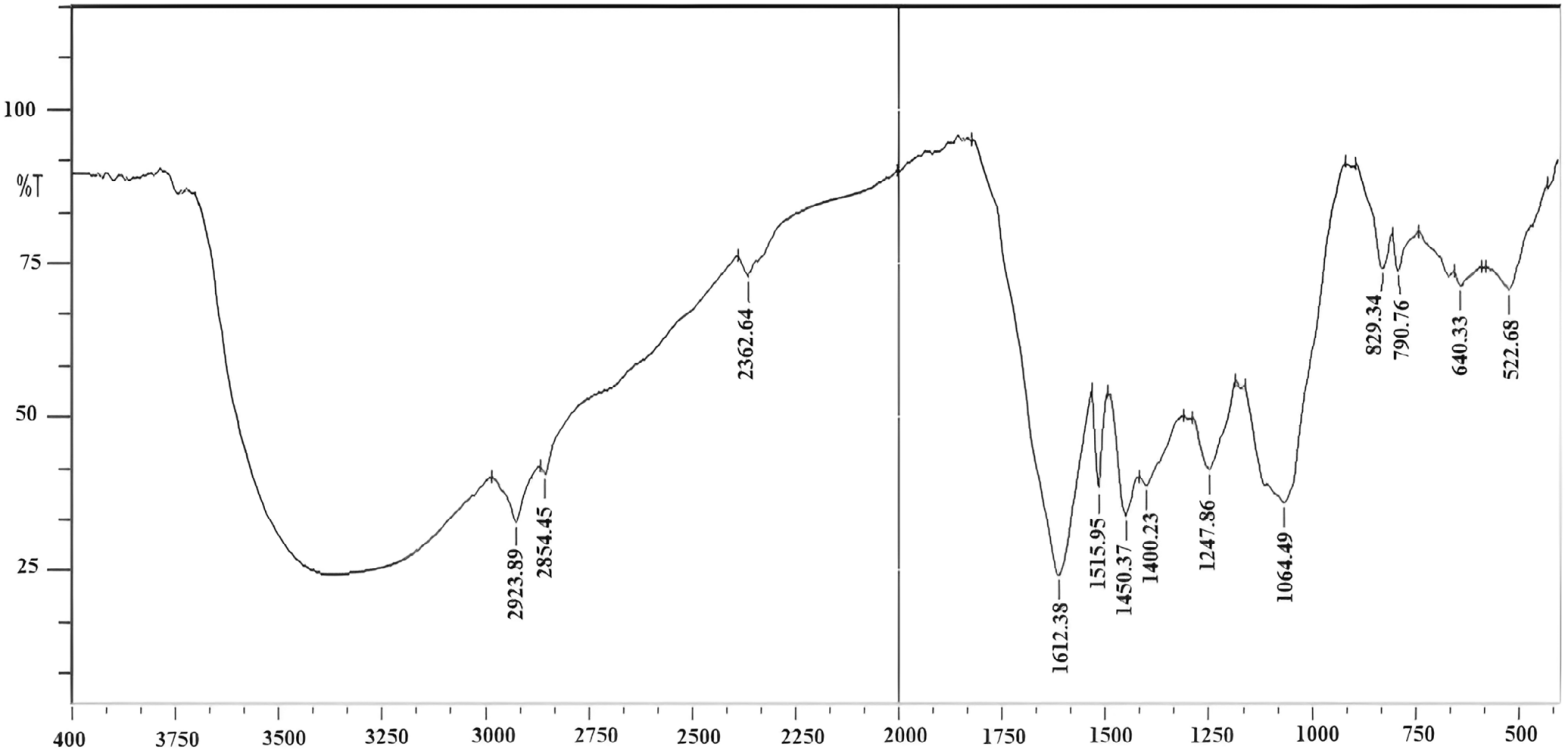
FT‐IR spectrum of pomegranate peel extract.

The FT‐IR spectrum of pomegranate peel exhibited characteristic absorption bands corresponding to various functional groups present in its composition. Figure 2 shows the distinctive peak of functional groups like hydroxyl (‐OH), carbonyl (C=O), and the fingerprint peak around 500–1200 cm^−1^. These spectral features putatively identified the presence of bioactive compounds, including polyphenols, flavonoids, and glycosidic linkages, in pomegranate peel.

### 3.5 LC–MS Screening and Fragmentation

**FIGURE 3:**
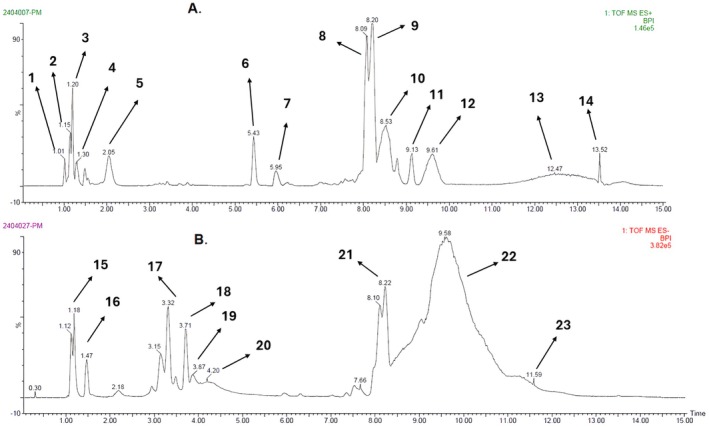
Positive (A) and negative (B) mode chromatograms of the extract. The numbers (**1–23**) in this figure are equivalent to the SN of Table 3, which represents the names of putatively identified phytoconstituents.

**FIGURE 4:**
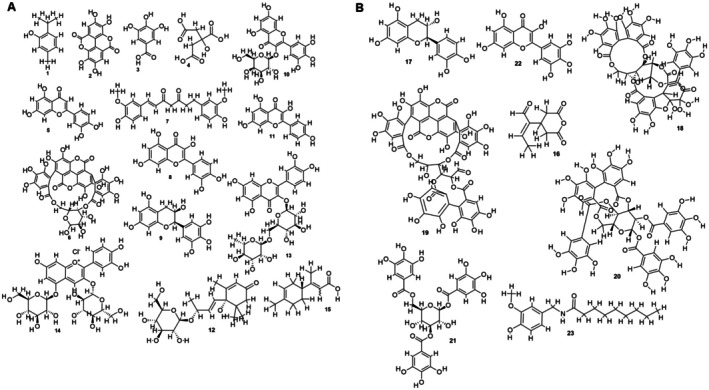
(A) Putatively identified phytoconstituents of pomegranate (**1–15**). (B) Putatively identified phytoconstituents of pomegranate (**16–23**).

The LC–MS analysis of pomegranate peel uncovered a significant variety of phytochemicals, such as phenolic acids, flavonoids, tannins, ellagitannins, anthocyanins, and terpenes, all detected in both positive and negative ionization modes (Table 3). We putatively identified 23 phytoconstituents based on m/z value, and about 48 phytoconstituents were reported from published articles and presented in (Table S1 and Figures S3–S56). Among the flavonoids, we found luteolin (RT 2.05 min, m/z 286), quercetin (RT 8.09 min, m/z 302), epigallocatechin (RT 8.20 min, m/z 306), and kaempferol (RT 9.13 min, m/z 286). We also putatively identified cyanidin‐3,5‐di‐O‐glucoside (RT 13.52 min, m/z 611), a significant anthocyanin that contributes to the red color of pomegranates. Additionally, among the ellagitannins, punicalin (RT 5.43 min, m/z 782) was detected, showing major fragment ions at m/z 481 and 191 (Figure 3). The 2D structure of the putatively identified 23 phytoconstituents is depicted in Figure 4A,B.

**TABLE 3:** Putatively identified phytoconstituents from positive and negative mode ionization.

SN	Name of Predicted compounds	Retention time	m/z value	Fragmentation	Classification
*Positive mode ionization (M + H)* ^+^					
1.	Thymol	1.01	150	123, 111, 110	Monoterpene
2.	Ellagic acid	1.15	302	302	Phenolic acid
3.	Gallic acid	1.20	170	170	Phenolic acid
4.	Citric acid	1.30	192	192	Carboxylic acid
5.	Luteolin	2.05	286	287	Flavonoid
6.	Punicalin	5.43	782	481, 191	Ellagitannin
7.	Dihydrocurcumin	5.95	370	268, 209, 149	Polyphenol
8.	Quercetin	8.09	302	301	Flavanol
9.	Epigallocatechin	8.20	306	305	Flavonoid
10.	Myricetin‐3‐galactoside	8.53	480	463, 393, 388, 291, 147, 130	Flavanol
11.	Kaempferol	9.13	286	224, 212, 141, 93	Flavonoid
12.	Roseoside	9.61	387	363, 347, 194, 174, 124	Glycoside
13.	Rutin	12.47	610	570, 224, 142, 114	Flavonoid
14.	Cyanidin‐3,5‐di‐o‐glucoside	13.52	611	449, 301, 266, 183, 142, 114	Anthocyanin
*Negative mode ionization (M‐H)* ^*−*^					
15.	Limonene carboxylic acid	1.18	182	182	Carboxylic acid
16.	Jasmin anhydride	1.47	182	182	Anhydride
17.	Catechin	3.32	290	277, 147, 139, 130	Flavonoid
18.	Granatin b	3.71	952	541	Tannin
19.	Punicalagin	3.87	1084	541, 301	Tannin
20.	Punicafolin	4.20	938	756, 643, 488, 303, 175, 142	Ellagitannin
21.	Gallotannin	8.22	636	520, 304, 284, 174, 130	Tannin
22.	Myricetin	9.58	318	318	Flavonoid
23.	Desmethyldihydrocapsaicin	11.59	293	293	Capsaicinoid

### 3.6 α‐Amylase Inhibitory Activity

In this study, acarbose was shown to exhibit a greater α‐amylase percentage inhibition value of 76.2349 ± 0.0064 than the pomegranate peel extract, 55.6742 ± 0.0058 at a concentration of 250 μg/mL. The IC_50_ value of pomegranate peel extract was found to be higher than the IC_50_ value of acarbose, that is, 222.19 μg/mL and 137.74 μg/mL, respectively, which are represented in Table 4.

**TABLE 4:** Percentage inhibition of α‐amylase activity of different concentrations of standard drug (acarbose) and plant extract, and IC_50_ value of standard drug and plant extract.

Concentration (μg/mL)	Percentage α‐amylase inhibition and IC_50_ value			
	Acarbose % inhibition	IC_50_ (μg/mL)	Extract % inhibition	IC_50_ (μg/mL)
50	28.70 ± 0.006	137.74	19.49 ± 0.005	222.19
100	43.12 ± 0.004		24.96 ± 0.0041	
150	52.60 ± 0.0025		34.57 ± 0.0045	
200	63.68 ± 0.0080		47.12 ± 0.003	
250	76.23 ± 0.0064		55.67 ± 0.0058	

*Note:* Comparison of the percentage inhibition of α‐amylase and IC_50_ value between the standard and the extract of varying concentrations from 50 to 250 μg/mL. The results were expressed in mean ± SD (*n* = 3).

### 3.7 Molecular Docking

**FIGURE 5:**
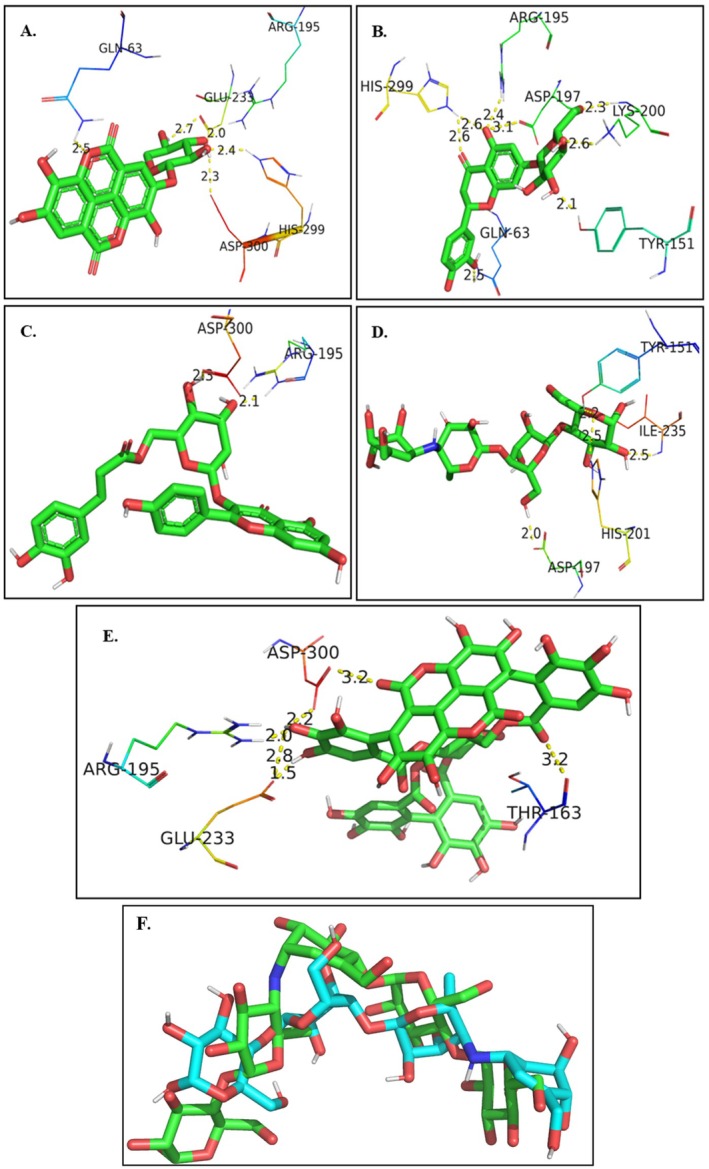
3D binding interactions of key phytochemicals with α‐amylase. Ellagic acid‐O‐xylopyranoside forms multiple hydrogen bonds with catalytic residues GLU233 and ASP300 as well as interactions with GLN63 and ARG195, indicating strong stabilization within the active site (A). Eriodictyol‐7‐O‐glucoside engages ASP197, LYS200, TYR151, GLU63, HIS299, and ARG195, showing extensive hydrogen bonding within the substrate‐binding pocket (B). Kaempferol 3‐(6″‐caffeoylglucoside) binds through hydrogen bonds with ASP300 and ARG195, suggesting a compact and stable interaction (C). Reference drug acarbose exhibits strong binding to TYR151, ILE235, HIS201, and ASP197, reflecting its well‐known inhibitory mechanism (D). Punicalagin displays the highest interaction density, forming hydrogen bonds with ARG195, GLU233, ASP300, and THR163, confirming its stable and high‐affinity binding mode (E). Distances are shown in Å and represent hydrogen bond lengths. Protein residues are colored by atom type: Nitrogen (blue), oxygen (red), carbon (green for ligand and cyan/orange for protein side chains), and sulfur (yellow). Ligands are shown in green stick representation, and hydrogen bond interactions are indicated by dashed lines. (F) Superimposition of the redocked ligand (acarbose, blue) and the native co‐crystallized ligand (green) within the active site of the target protein.

The binding score of 72 phytochemicals from the pomegranate peel was calculated using AutoDock Vina v1.2.0 software against the α‐amylase enzyme. The binding score ranges from −6.0 to −11.6 kcal/mol (Table S1). Ten phytoconstituents exhibited significantly strong interaction, having a binding score of −9.5 to −10.6 kcal/mol when compared to the acarbose −7.9 kcal/mol (Table 5). Acarbose was used as a reference compound as it was a co‐crystal ligand of α‐amylase protein (PDBID 3BAJ). Punicalagin showed the strongest interaction against α‐amylase (3BAJ) with a binding score of −11.6 kcal/mol. Figure 5A–E displays the molecular interaction of four best phytochemicals and acarbose. 2D interaction of remaining phytochemicals is given in (Figures S57–S61).

**TABLE 5:** Binding score, interactive amino acids, and number of hydrogen bonds of phytochemicals against α‐amylase enzyme.

Phytochemicals	Binding score (kcal/mol)	Interactive amino acids	No of H‐bonds
Punicalagin	−11.6	ASP‐300, ARG‐195, GLU‐233, THR‐165	6
Ellagic acid‐o‐xylopyranoside	−10.6	ARG‐195, HIS‐299, ASP‐300, GLU‐233, GLN‐63	5
Kaempferol 3‐(6″‐caffeoylglucoside)	−10.1	ARG‐195 and ASP‐300	3
Eriodictyol‐7‐o‐glucoside	−9.9	ARG‐195, ASP‐197, HIS‐299, TYR‐151, LYS‐200, GLN‐63	6
Tiliroside	−9.9	GLN‐63, HIS‐299, ASP‐300, GLU‐233, LYS‐200	5
Apigetrin	−9.8	HIS‐299, ASP‐300, LYS‐200	3
Luteolin‐7‐glucoside	−9.8	HIS‐299, ASP‐300, LYS‐200, GLU233	4
Odoratone	−9.7	GLU‐233, TRP‐59	2
Luteolin	−9.5	ASP‐197, HIS299	2
Kaempferol 3‐(2″‐galloyl‐alpha‐l‐arabinopyranoside)	−9.5	GLU‐233, ASP‐197, HIS‐299, GLN‐63, HIS‐300, LYS‐200	6
Punicalin	−7.8	GLN‐63	3
Reference acarbose	−7.9	ASP‐197, HIS‐202, ILE‐235, TYR‐151	4

#### 3.7.1 Docking Protocol Validation Analysis

The docking protocol was validated using a redocking approach, where the co‐crystallized ligand (acarbose) was removed from the protein structure and re‐docked into its original binding site using the same docking parameters intended for virtual screening. The resulting pose was then superimposed on the native co‐crystal conformation, and the root‐mean‐square deviation (RMSD) between them was calculated. An RMSD of 1.78 Å was obtained (blue: redocked ligand, green: native co‐crystal ligand), indicating a high degree of accuracy and confirming that the docking protocol reliably reproduces the experimentally determined binding orientation.

### 3.8 MD Simulation

**FIGURE 6:**
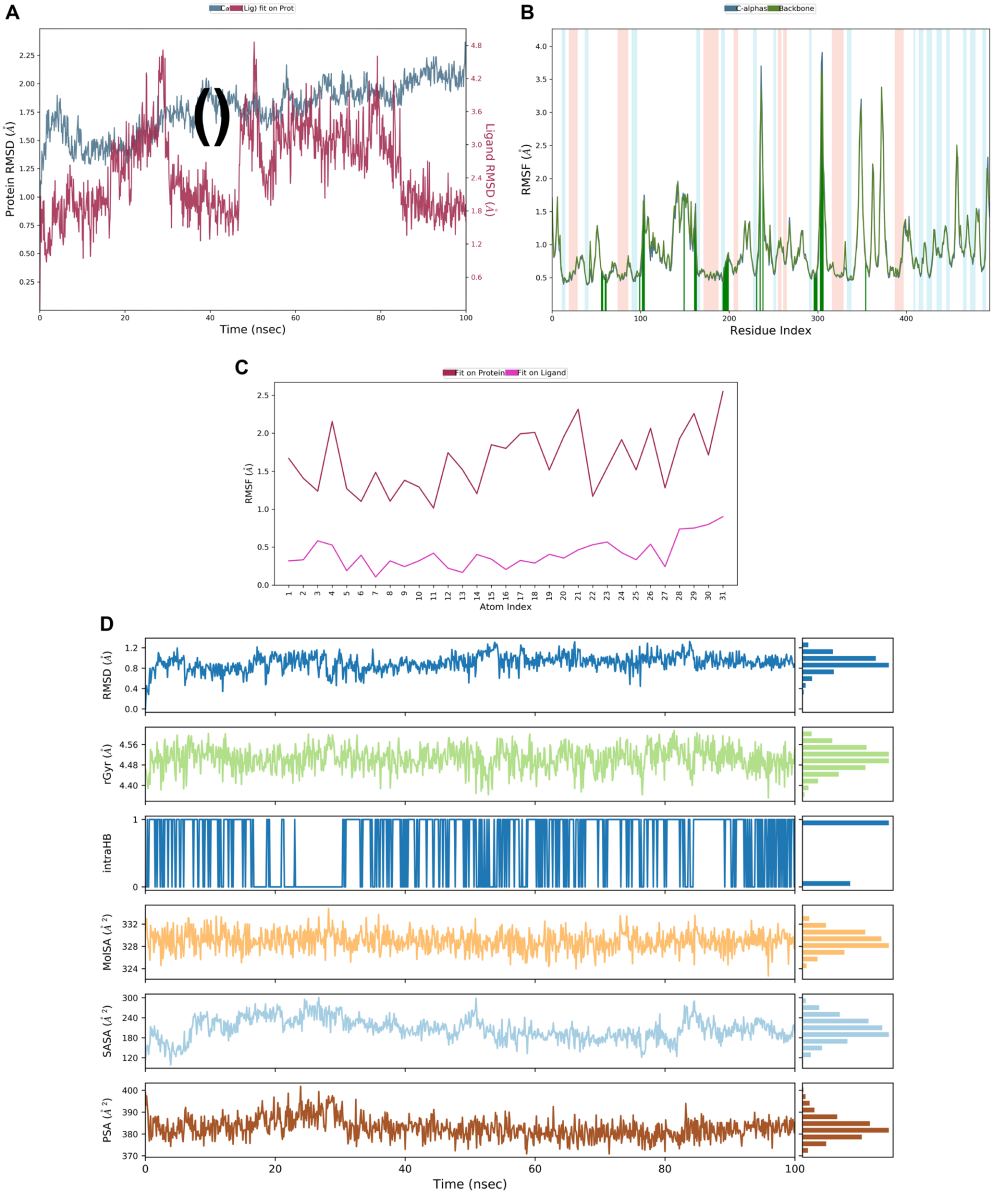
MD simulation with RMSD, RMSF, *r*Gyr, and SASA of punicalagin against α‐amylase.

The MD simulation of punicalagin bound to the α‐amylase enzyme over a 100 ns time scale provided insights into the stability and interactions of the ligand‐protein complex. Figure 6A shows the protein‐ligand RMSD, indicating stable binding with minor ligand fluctuations at 10–12 ns. Figure 6B presents RMSF, highlighting flexible residues mainly in loop regions. Figure 6C illustrates ligand atom fluctuations, where atoms interacting with the protein exhibit lower RMSF, suggesting stable contacts. The stable radius of gyration (rGyr) indicates a compact structure, while consistent solvent‐accessible surface area (SASA) and polar surface area (PSA) values confirm solubility, stability, and bioavailability, supporting punicalagin's role as an α‐amylase inhibitor (Figure 6).

### 3.9 MM/GBSA Energy Analysis

The MM/GBSA analysis yields detailed information about the binding free energies of the punicalagin‐3BAJ complex. The MM/GBSA analysis of punicalagin against the α‐amylase protein reveals a favorable binding free energy (Δ*G* bind = −86.67 kcal/mol), indicating strong interaction stability. The lipophilic contribution (−53.09 kcal/mol) is the dominant stabilizing force, suggesting that hydrophobic interactions play a crucial role in binding (Table 6).

**TABLE 6:** MM‐GBSA binding free energy components of punicalagin‐α‐amylase complex.

Parameter	Mean value (kcal/mol)
Δ*G* bind	−86.67
Coulomb energy	25.07
H‐Bond energy	−2.01
Lipophilic energy	−53.09
Solvation energy	−10.18
Ligand efficiency	−1.87

## 4 Discussion

This study explored the phytochemical composition of pomegranate peel extract using LC–MS, alongside in vitro and in silico evaluations of α‐amylase inhibitory activity. The results demonstrate that the extract possesses strong antidiabetic potential, with in vitro activity comparable to acarbose, the reference standard. Integration of the phytochemical profiling and molecular docking/MD analyses suggests that this bioactivity is largely attributable to ellagitannins—particularly punicalagin—supported by other phenolics and flavonoids identified in the extract.

The Soxhlet extraction with 80% methanol produced a higher yield than many previous reports, which may be attributed to solvent polarity, extraction duration, and the maturity stage of the collected fruits. The extract contained abundant phenolic compounds, confirmed by preliminary phytochemical screening and quantification of total phenolic content (TPC). The measured TPC (49.54 ± 0.74 mg QE/g) fell within the range reported in related studies (Wang et al. 2011; Ranjha et al. 2020) but was lower than some high‐ellagitannin cultivars (Ardekani et al. 2011). Pomegranates from subtropical Nepal, where this study's sample originated, possess a distinct phytochemical profile influenced by high‐altitude conditions, which favor ellagitannin accumulation over anthocyanins. This can be supported by the fact that pomegranate trees need plenty of sunlight and well‐drained soil to flourish in semi‐arid to subtropical regions. Temperature plays an important role in extraction. Generally, a rise in phenolic content can be observed with increasing temperature (Luitel and Khanal 2023).

The methanolic extract inhibited α‐amylase in a dose‐dependent manner however, an IC_50_ value was higher than acarbose. This strong activity aligns with the high levels of punicalagin and related ellagitannins, which are known to modulate carbohydrate digestion. A study identified ellagic acid, gallic acid, and kaempferol as key bioactives, supporting the antidiabetic potential of pomegranate extract (Shi et al. 2022). Similarly, Pottathil et al. attributed the antidiabetic and antioxidant properties of pomegranate peel extract to the presence of phenolic compounds, particularly gallic acid, ellagic acid, and apigenin (Pottathil et al. 2020). These comparative findings highlight not only the variability in antidiabetic activity based on extraction methods and dosage but also support the potential of pomegranate peel as a multifunctional therapeutic agent, for managing diabetes and its complications.

On the other hand, the somewhat lower potency of the extract compared to acarbose may relate to the relative abundance of highly active phenolics such as ellagic acid, catechin, vanillin, and vanillic acid, as suggested by earlier studies (Laaraj et al. 2022; Di Sotto et al. 2019). Variations reported in the literature for α‐amylase inhibition by pomegranate peel extracts are likely due to differences in extraction solvents, phenolic composition, and formulation strategies (Gościniak et al. 2024). For instance, nanoemulsion, nanoparticle (Royapuram Parthasarathy et al. 2023), and anthocyanin‐enriched formulations have been shown to enhance activity by improving solubility and bioavailability (Eid et al. 2024).

**FIGURE 7:**
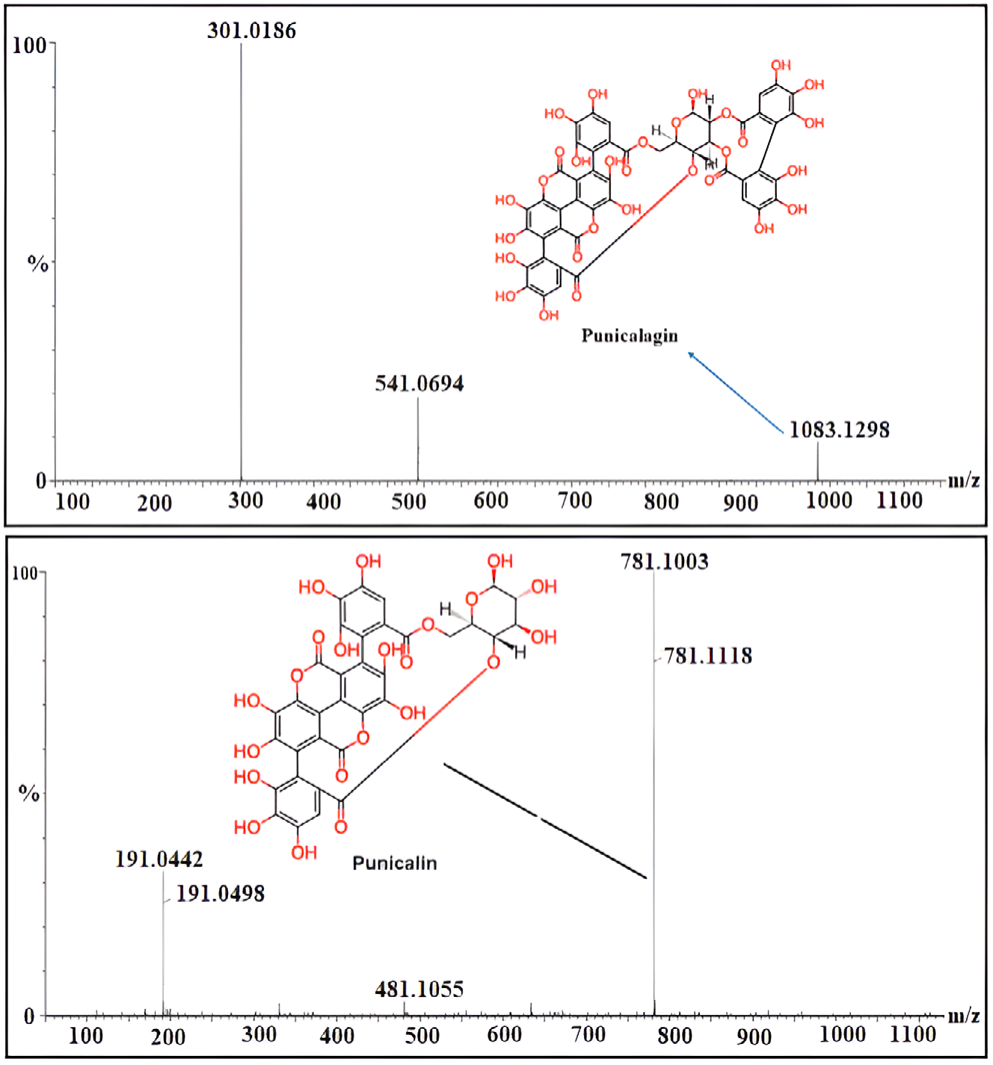
MS spectra of punicalagin and punicalin.

LC–MS analysis putatively identified the presence of multiple phenolic acids, flavonoids, tannins, and anthocyanins. Punicalagin and punicalin, both hydrolysable ellagitannins, were identified as major constituents. The fragmentation patterns supported their structural identification, showing characteristic glycosidic cleavage and production of ellagic acid fragments (Figure 7). The profile also included gallic acid, catechin, kaempferol derivatives, and anthocyanins such as cyanidin‐3,5‐di‐O‐glucoside. Notably, Nepalese pomegranate peels showed a narrower but more ellagitannin‐rich profile compared to Chinese cultivars (Man et al. 2022), which tend to exhibit a broader flavonoid diversity. This difference may influence both antioxidant and enzyme‐inhibitory activities.

Molecular docking targeted the α‐amylase active site known to bind acarbose. Acarbose helps to slow carbohydrate breakdown and glucose absorption in the small intestine. Acarbose is a competitive inhibitor of intestinal α‐glucosidase and pancreatic α‐amylase enzymes. Acarbose, when bound to the pancreatic α‐amylase enzyme binding site, modulates starch breakdown (Magaji et al. 2020). Hence, the acarbose binding site was chosen for docking. The binding site consists of ten amino acids which include TRP‐59, TYR‐62, GLN‐63, THR‐163, ARG‐195, LYS‐200, GLU‐233, GLU‐240, HIS‐299, and ASP‐300 (Figure S7). Acarbose interacts through hydrogen bonding with 9 amino acids and pi‐donor hydrogen bonding with TYR‐59 (X‐ray crystallographic interaction/co‐crystal ligand interaction) (Vorhees and Williams 2006). Thus, it slows down the breakdown of carbohydrates.

**FIGURE 8:**
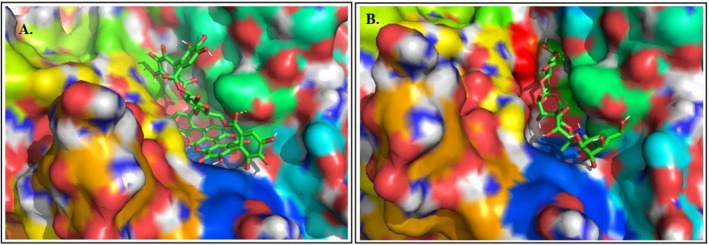
Binding interaction of punicalagin to the acarbose binding site (A), binding interaction of acarbose (B).

Punicalagin exhibited the strongest binding affinity (−11.6 kcal/mol), forming multiple hydrogen bonds with key catalytic residues (ASP‐300, ARG‐195, GLU‐233, and THR‐165). This interaction pattern closely resembles the interaction pattern of acarbose (Figure 8), suggesting a competitive inhibition mechanism. Gull et al. explained the role of punicalagin in imparting the α‐amylase inhibitory effect through in silico validation. They reported that the binding interaction of punicalagin against α‐amylase is very significant in slowing down carbohydrate metabolism. Furthermore, to improve the inhibitory activity of the extract, they observed that the support of punicalin (−11.3 kcal/mol) and ellagic acid (−8.3 kcal/mol) is crucial (Gull et al. 2023). This shows the synergistic effect of phytochemicals. In our study, other constituents—kaempferol 3‐(6″‐caffeoylglucoside), ellagic acid‐O‐xylopyranoside, and eriodictyol‐7‐O‐glucoside—also demonstrated strong affinities, reinforcing the likelihood of pomegranate possessing synergistic effects.

**FIGURE 9:**
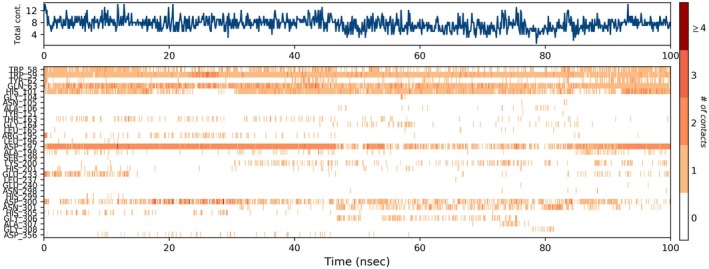
A timeline representation of the interactions and contacts (H‐bonds, hydrophobic, ionic, water bridges) of punicalagin against α‐amylase.

MD simulations confirmed the stability of the punicalagin–α‐amylase complex, with both protein and ligand RMSD values stabilizing (Figure 9) over the simulation period. The MM/GBSA analysis yielded a favorable binding free energy (ligand efficiency −1.87 kcal/mol), supporting the sustained interaction predicted by docking. These findings align with prior reports, which indicated that glycosylated ellagitannins maintain both strong binding and structural stability against α‐amylase (Cardullo et al. 2020). Such integration underscores the utility of combining chemical profiling with computational and biological assays for identifying bioactive leads (Glick and Jacoby 2011; Wawer et al. 2014).

Pomegranate peel is generally regarded as safe when consumed in reasonable quantities. Jahromi et al. conducted an in vivo study to evaluate the toxicity of pomegranate peel extract. Their findings indicated that the extract is safe and exhibited no signs of toxicity (Jahromi et al. 2015). Furthermore, various in vitro and in silico research showed that the pomegranate peel extracts at a concentration of 300 μg/mL have no obvious cytotoxic and hepatotoxic effects (Batool et al. 2024; Qabaha et al. 2019).

## 5 Limitation and Future Prospective

Due to resource limitations, we could not perform the nuclear magnetic resonance (NMR) experiment to confirm and characterize the metabolomic finding. Our study was designed to analyze the secondary metabolites by comparing the RT with published articles and online databases. We have predicted the phytoconstituents present in the extract. However, a confirmatory study can be done through isolation and NMR spectroscopic characterization. The potency, efficacy, toxicity, and dosing of individual phytoconstituents can be further investigated in future research. Further analysis, such as MM‐PBSA calculations, could provide deeper insights into the binding free energy of pomegranate peel against the proteins. Future research should focus on isolating individual bioactive compounds, conducting in vivo studies, and exploring formulation strategies to enhance the bioavailability and therapeutic efficacy of pomegranate peel.

## 6 Conclusion

The study successfully profiled the phytochemical composition of pomegranate peel extract of Nepalese origin and demonstrated an α‐amylase inhibitory (IC_50_ = 222.19 μg/mL) potential through in vitro and computational approaches. As confirmed by docking and MD simulations, the key phytoconstituents in the pomegranate peel used in this study, particularly punicalagin (−11.6 kcal/mol), ellagic acid‐O‐xylopyranoside (−10.6 kcal/mol), and kaempferol 3‐(6″‐caffeoylglucoside) (−10.1 kcal/mol), exhibited strong enzyme binding and inhibitory activities. Furthermore, MD simulation confirmed the stability of the punicalagin‐α‐amylase complex at 100 ns. The results of our study suggest that pomegranate peel extract could serve as a natural alternative for managing postprandial hyperglycaemia.

## Author Contributions

**Shankar Thapa:** conceptualization (lead), data curation (lead), formal analysis (lead), methodology (lead), software (lead), writing – original draft (lead), writing – review and editing (lead). **Deepti Pandey:** conceptualization (equal), formal analysis (equal), investigation (equal), methodology (equal), writing – original draft (equal). **Mahalakshmi Suresha Biradar:** formal analysis (equal), methodology (equal), software (equal), validation (equal). **Monica Arora:** data curation (equal), investigation (equal), validation (equal), writing – review and editing (equal). **Shithin Ann Varghese:** data curation (equal), investigation (equal), resources (equal), validation (equal), writing – review and editing (equal). **Akila Elias:** formal analysis (equal), investigation (equal), visualization (equal), writing – review and editing (equal). **Aleesha Mujeeb Shaheen:** data curation (equal), methodology (equal), writing – review and editing (equal). **Somashekhar M. Metri:** formal analysis (equal), investigation (equal), writing – review and editing (equal). **Shaik Sadik:** validation (equal), visualization (equal), writing – review and editing (equal). **Sreeharsha Nagaraja:** validation (equal), visualization (equal), writing – review and editing (equal). **Sharmila Gote:** formal analysis (equal), visualization (equal), writing – review and editing (equal). **Bipindra Pandey:** visualization (equal), writing – review and editing (equal).

## Ethics Statement

The authors have nothing to report.

## Conflicts of Interest

The authors declare no conflicts of interest.

## Supporting information


**Appendix S1:** fsn371191‐sup‐0001‐AppendixS1.docx.

## Data Availability

Available in Supporting Information.
