# Supplementary material for: Phytochemical Profile and α‐Amylase Inhibitory Activity of Pomegranate Peel Extract: An In Silico and In Vitro Investigation
Source: Food Sci Nutr. 2025 Nov 11;13(11):e71191. doi: 10.1002/fsn3.71191 (PMC12605963; doi:10.1002/fsn3.71191)
Supplement: Supplementary file 1 — Appendix S1: fsn371191‐sup‐0001‐AppendixS1.docx. [file FSN3-13-e71191-s001.docx]

**Research article**

**Phytochemical Profile and α-Amylase Inhibitory Activity of Pomegranate Peel Extract: An In-Silico and In-Vitro Investigation**

**Supplementary Data**

Table 1S: LC-MS predicted phytoconstituents and binding energy.

| **Phytoconstituents** | **MW** | **Reported m/z value** | **Ionization** | **Binding energy** |
| --- | --- | --- | --- | --- |
| Quercetin | 302 | 301 | M-H | -8.9 |
| Taxifolin | 304 | 304 | M | -9.2 |
| Trimethoxyflavone | 312 | 311 | M-H | -7.6 |
| Beta-Sitosterol | 414 | 413 | M-H | -8.9 |
| Myricetin 3-galactoside | 480 | 481 | M+H | -8.2 |
| Myricetin | 318 | 319 | M+H | -9.0 |
| Dihydrocurcumin | 370 | 371 | M+H | -7.8 |
| Ellagic Acid | 302 | 301 | M-H | -8.6 |
| Epigallocatechin | 306 | 305 | M-H | -8.7 |
| Kaempferol | 286 | 285 | M-H | -8.7 |
| Luteolin | 286 | 287 | M+H | -9.5 |
| Citric acid | 192 | 191 | M-H | -5.3 |
| Ellagic Acid 4-O-Xylopyranoside | 434 | 433 | M-H | -10.6 |
| Galloyl-glucose | 332 | 331 | M-H | -7.9 |
| Thymol | 150 | 151 | M+H | -6 |
| Apigetrin | 432 | 432 | M | -9.8 |
| Querciturone | 478 | 477 | M-H | -8.4 |
| Eriodictyol-7-O-glucoside | 450 | 449 | M-H | -9.9 |
| Genistein | 270 | 271 | M+H | -8.7 |
| Gallic acid | 170 | - | - | -6.1 |
| Catechin | 290 | 291 | M+H | -9.0 |
| Rutin | 610 | 611 | M+H | -8.9 |
| Punicic acid | 278 | 279 | M+H | -5.6 |
| Punicalin | 782 | 781 | M-H | -7.8 |
| Pedunculagin | 782 | 783 | M+H | -11.6 |
| Corilagin | 634 | 633 | M-H | -9.3 |
| Tiliroside | 594 | 595 | M+H | -9.9 |
| Procyanidin B1 | 578 | 577 | M-H | -8.4 |
| Datiscin | 594 | 595 | M+H | -9.2 |
| Limonene carboxylic acid | 180 | 181 | M+H |  |
| Naringenin | 272 | 272 | M | -9.1 |
| Tuberatolide A | 290 | 291 | M+H | -6.7 |
| Desmethyl dihydrocapsaicin | 293 | 293 | M | -6.7 |
| SB 218655 | 309 | 309 | M | -7.8 |
| Roseoside | 386 | 387 | M+H | -7.7 |
| Kaempferol-7-O-alpha-L-rhamnoside | 432 | 433 | M+H | -9 |
| Kaempferol-3-Glucoside | 448 | 449 | M+H | -8.4 |
| Luteolin-7-glucoside | 448 | 449 | M+H | -9.8 |
| Myzodendrone | 343 | 344 | M+H | -7.6 |
| Gibboside | 362 | 363 | M+H | -7.6 |
| Demethoxycurcumin | 338 | 337 | M-H | -8.4 |
| Methyl pheophorbide b | 620 | 619 | M-H | -9.0 |
| Dihydrokaempferol | 288 | 289 | M+H | -9 |
| Undulatoside A | 354 | 353 | M-H | -8.8 |
| Cyanidin | 287 | 287 | M | -9.0 |
| Cyanidin-3-O-galactoside | 449 | 449 | M | -8.3 |
| Cyanidin 3-O-glucoside | 449 | 449 | M | -8.3 |
| Cyanidin-3,5-di-O-glucoside (D49) | 611 | 612 | M+H | -8.3 |
| Isochondrodendrine | 594 | 595 | M+H | -9.1 |
| 3-Oxo-2-(2Z-Pentenyl) cyclopentyl propionic acid | 224 | 224 | M | -6.8 |
| Bakankoside | 381 | 381 | M | -7.5 |
| Cryptophycin 46 | 639 | 663 | M+Na | -10.1 |
| Octadecanedioic acid | 314 | 315 | M+H | -6.6 |
| Odoratone | 472 | 472 | M | -9.7 |
| Ebracteatoside A | 520 | 520 | M |  |
| Welloside (D58) | 700 | 702 | M+2 | -8.2 |
| Stellettin J | 452 | 453 | M+H | -8.3 |
| Yungensin E | 662 | 663 | M+H | -9.5 |
| TMC 2A | 570 | 570 | M | -6.3 |
| Jasminanhydride | 182 | 181 | M-H | -6.2 |
| Ginsenoside Rh7 | 618 | - |  | -12 |
| Tetragalloyl-alpha-D-glucose | 788 | 780 | M+H | -9.7 |
| Punicafolin | 938 | 938 | M | -12.2 |
| Gallotannin | 636 | 636 | M | -9.2 |
| Kaempferol 3-(6''-caffeoylglucoside) | 610 | 611 | M+1 | -10.1 |
| Phaeochromycin A | 312 | 311 | M-H | -8.1 |
| Kynapcin 13 (D69) | 266 | 265 | M-H | -6.7 |
| Catechin 3-O-alpha-L-rhamnoside | 436 | 437 | M+H |  |
| Kaempferol 3-(2''-galloyl-alpha-L-arabinopyranoside) | 570 | 570 | M | -9.5 |
| 2-Methoxyoctadecanoic acid | 314 | 315 | M+H | -6.1 |

**
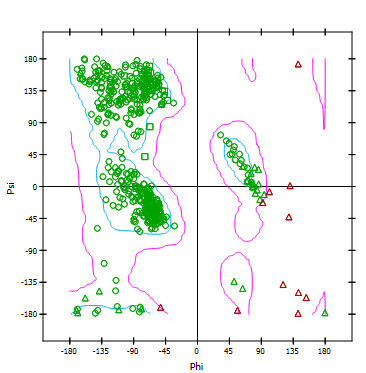
**

FIGURE 1S: Ramachandran plot of protein 3BAJ.

**
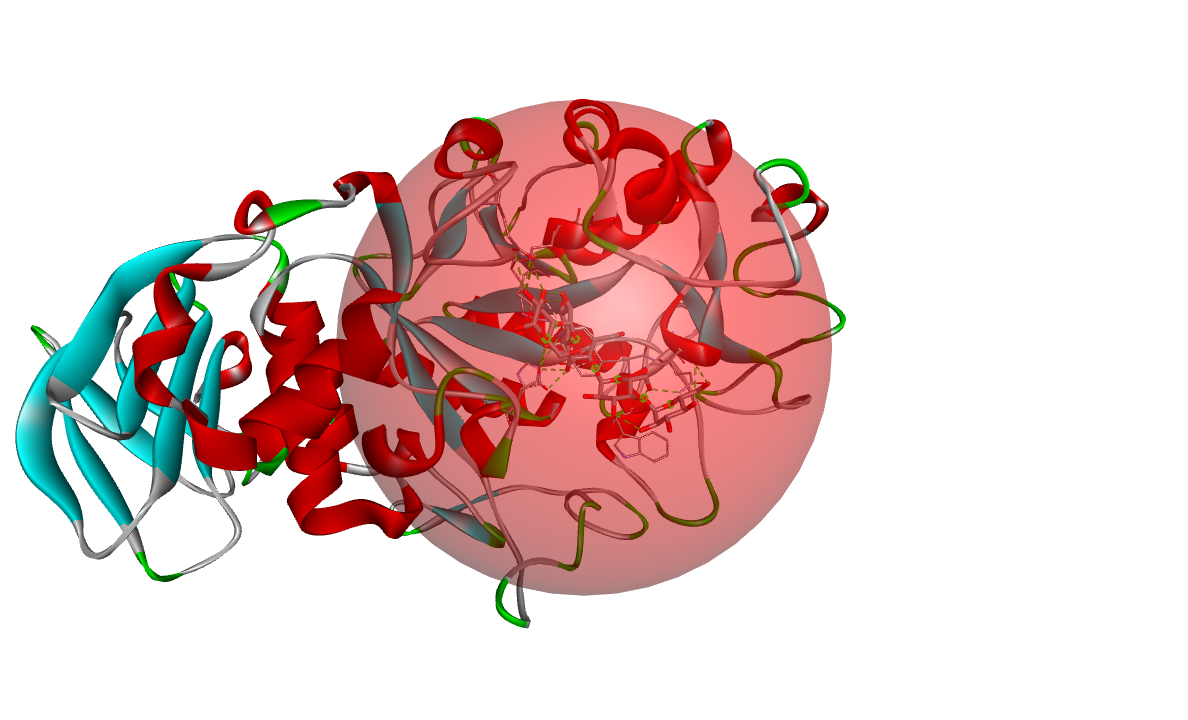
**

FIGURE 2S: Acarbose binding site of α-amylase.

**LCMS spectrum**

**
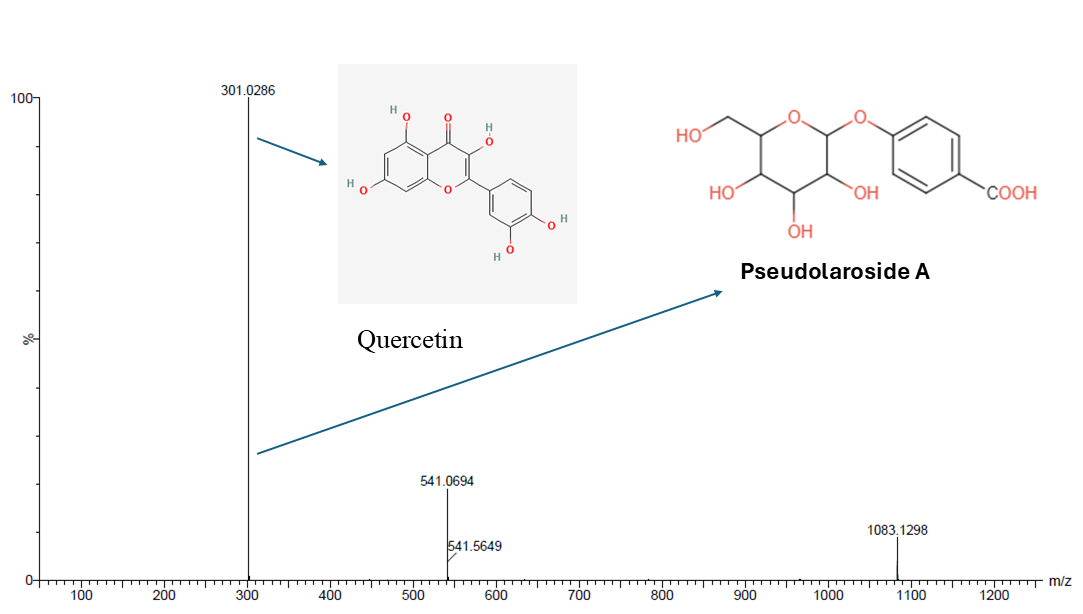
**

FIGURE 3S: LCMS spectra of Quercetin and Pseudolaroside A.

**
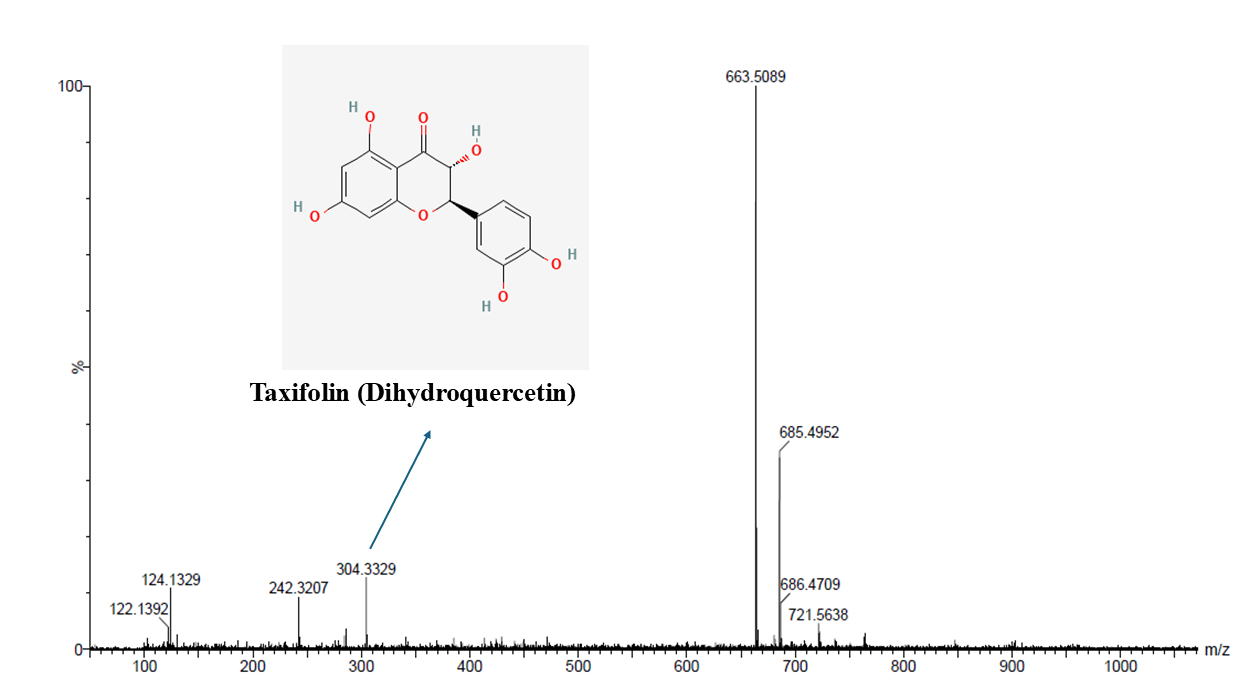
**

FIGURE 4S: LCMS spectra of Taxifolin.

**
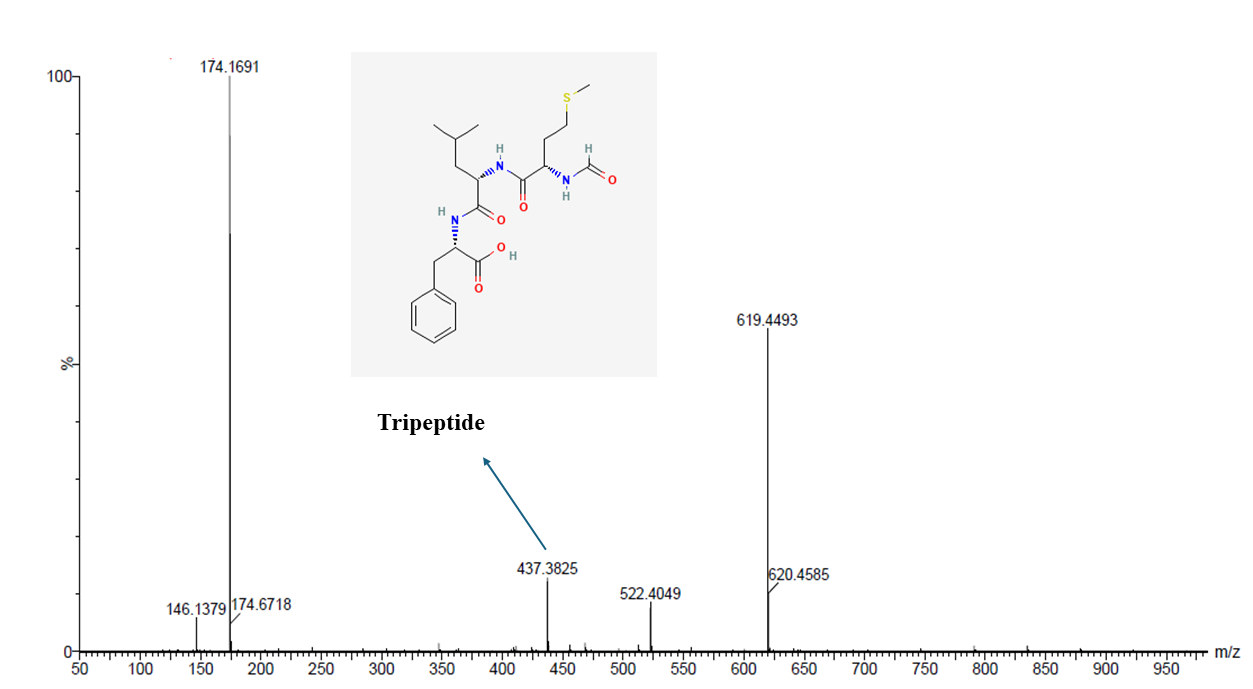
**

FIGURE 5S: LCMS spectra of Tripeptide.

**
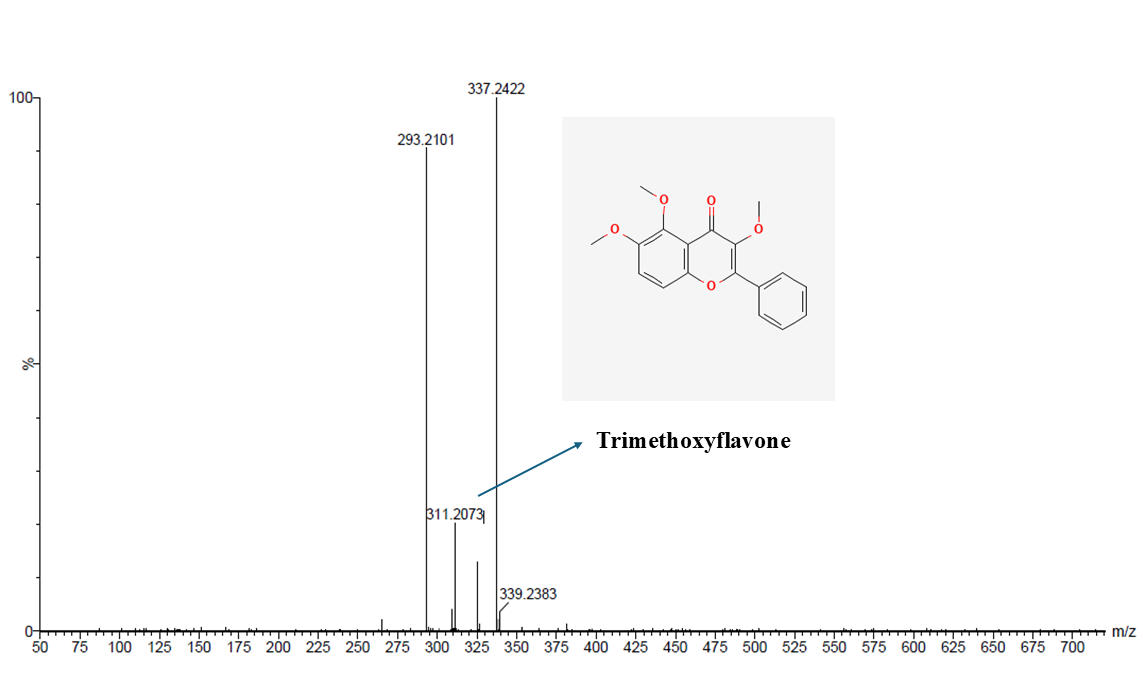
**

Figure 6S: LCMS spectra of Trimethoxyflavone.

**
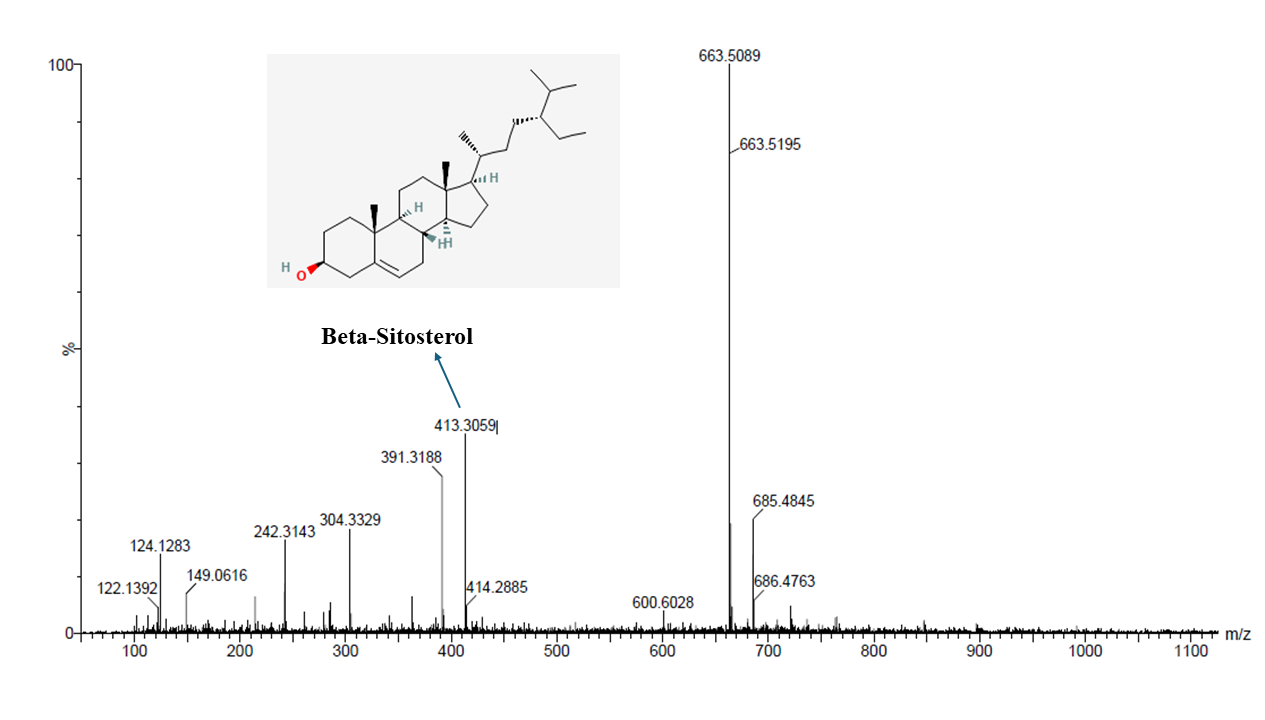
**

Figure 7S: LCMS spectra of Beta-Sitosterol.

**
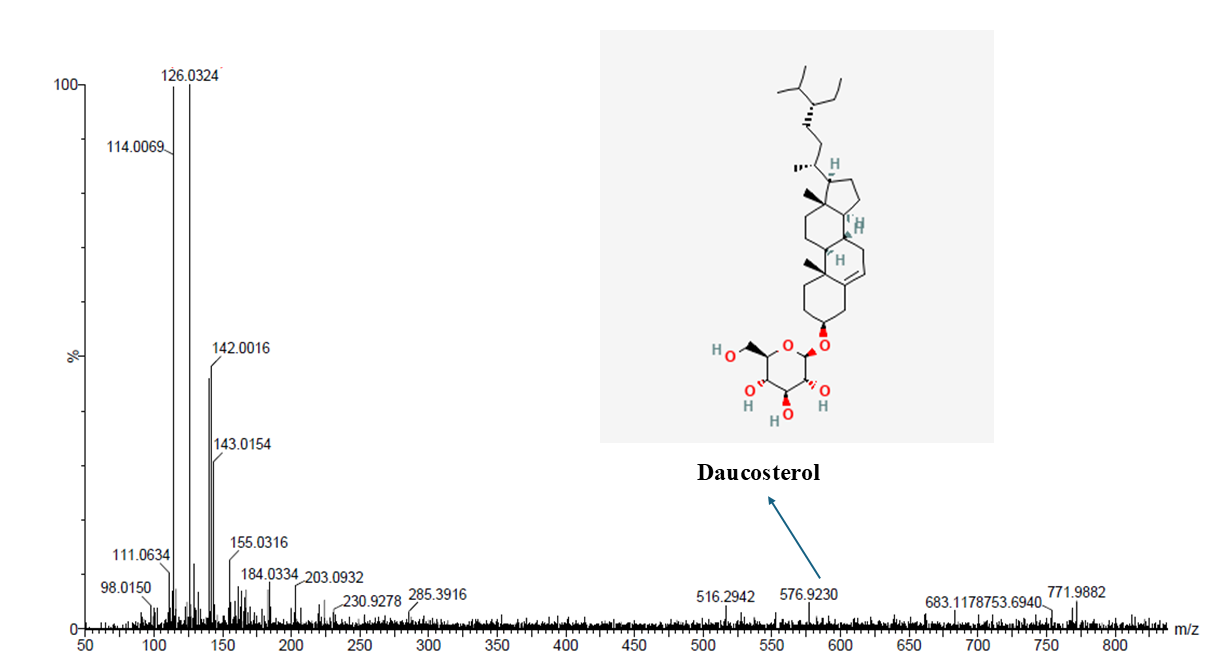
**

Figure 8S: LCMS spectra of Daucosterol.

**
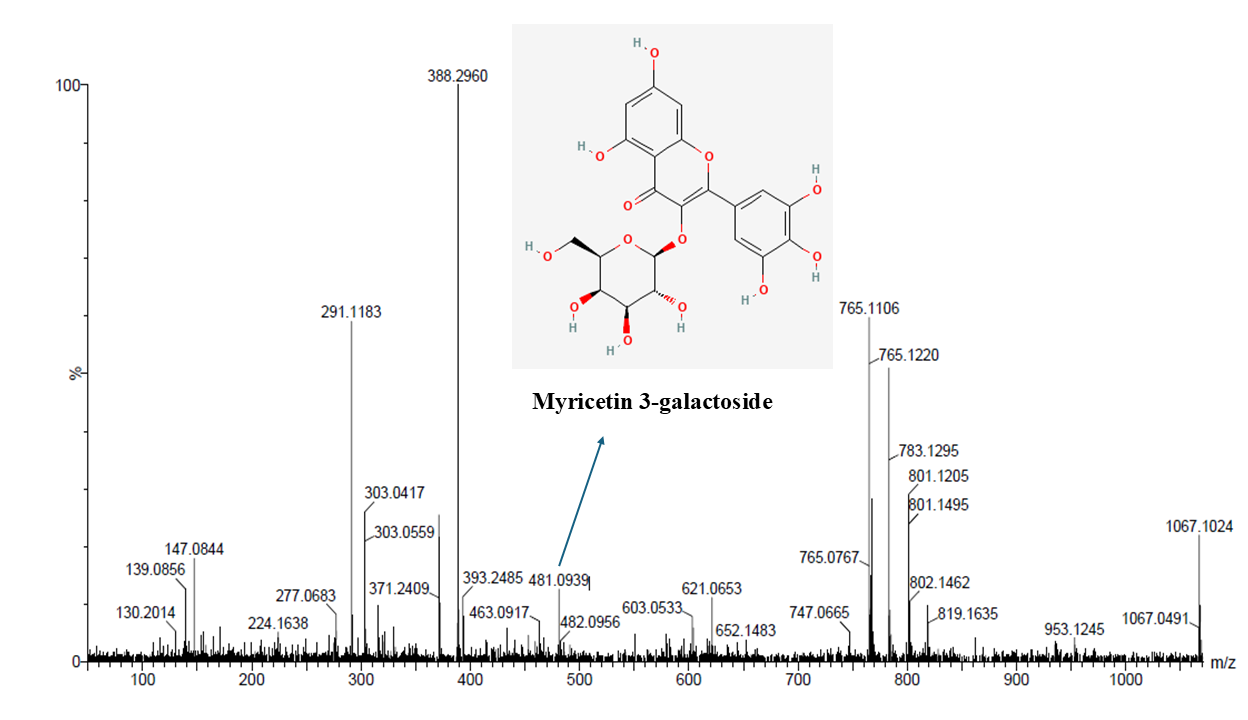
**

Figure 9S: LCMS spectra of Myricetin 3-galactoside.

**
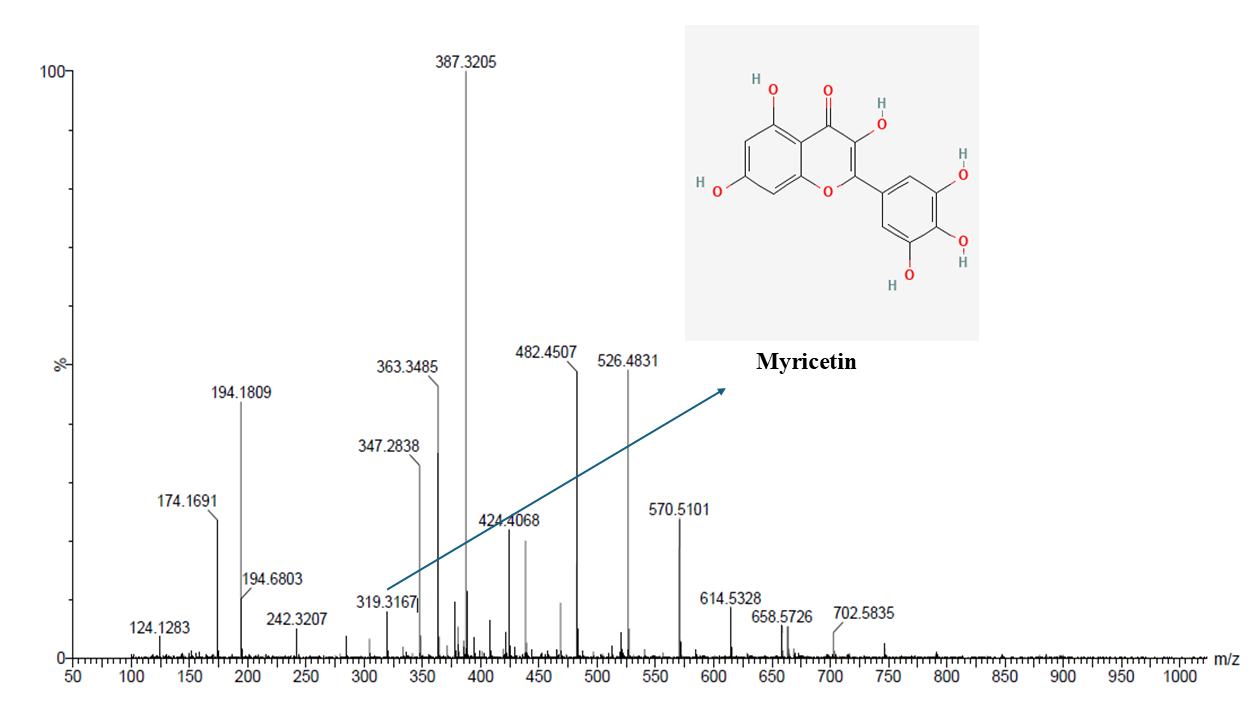
**

Figure 10S: LCMS spectra of Myricetin.

**
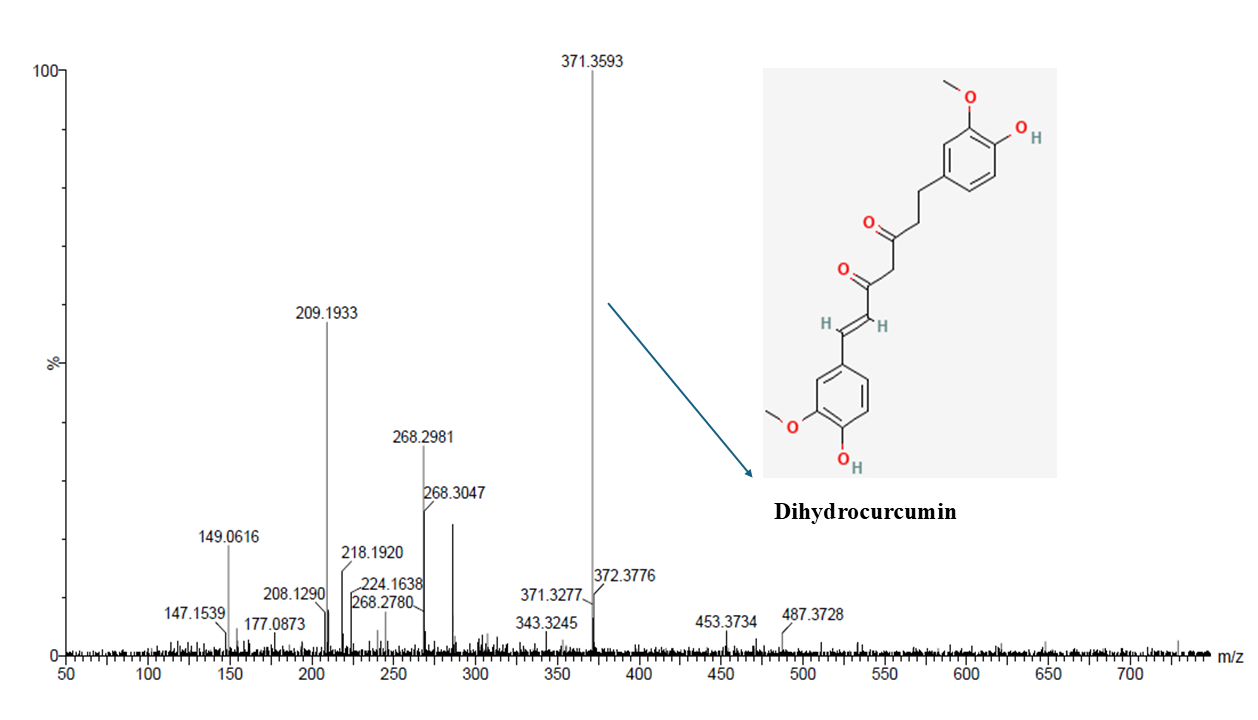
**

Figure 11S: LCMS spectra of Dihydrocurcumin.

**
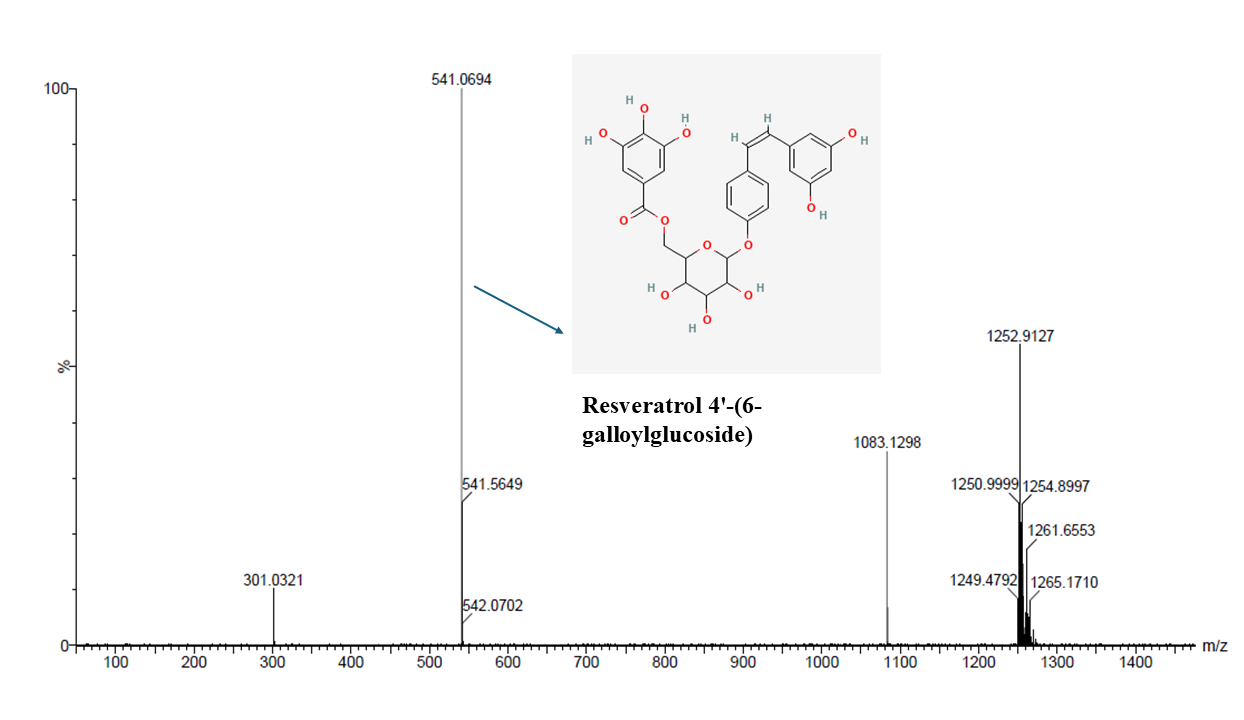
**

Figure 12S: LCMS spectra of Resveratrol 4’-(6-galloylglucoside)

**
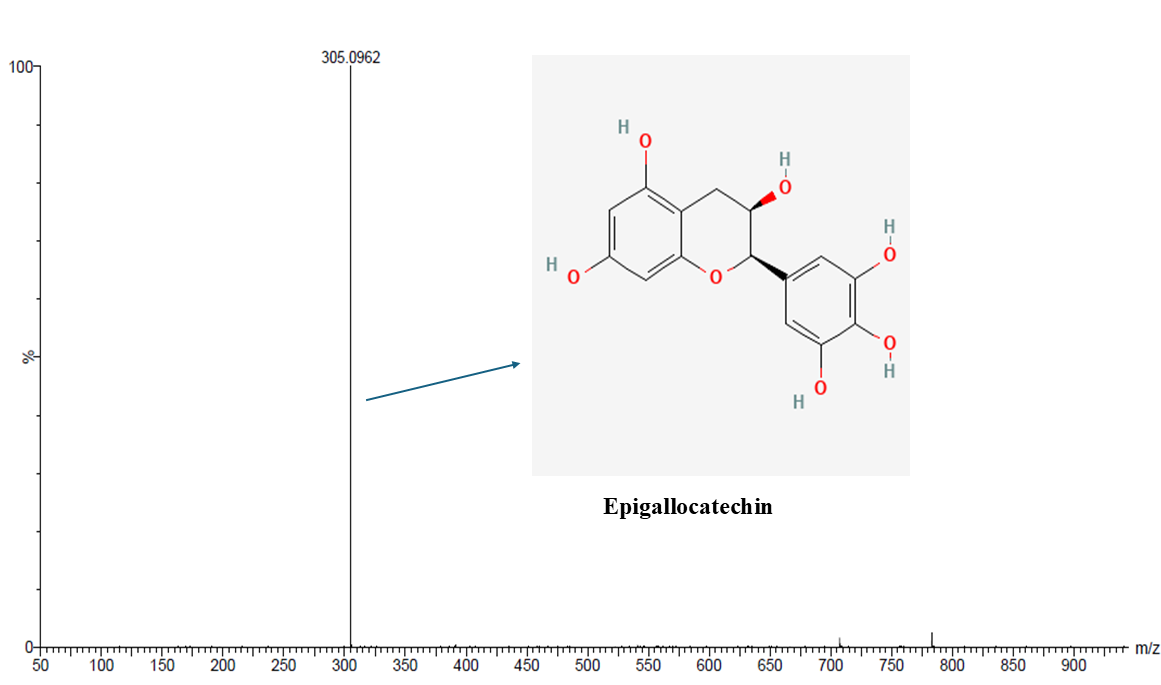
**

Figure 13S: LCMS spectra of Epigallocatechin.

**
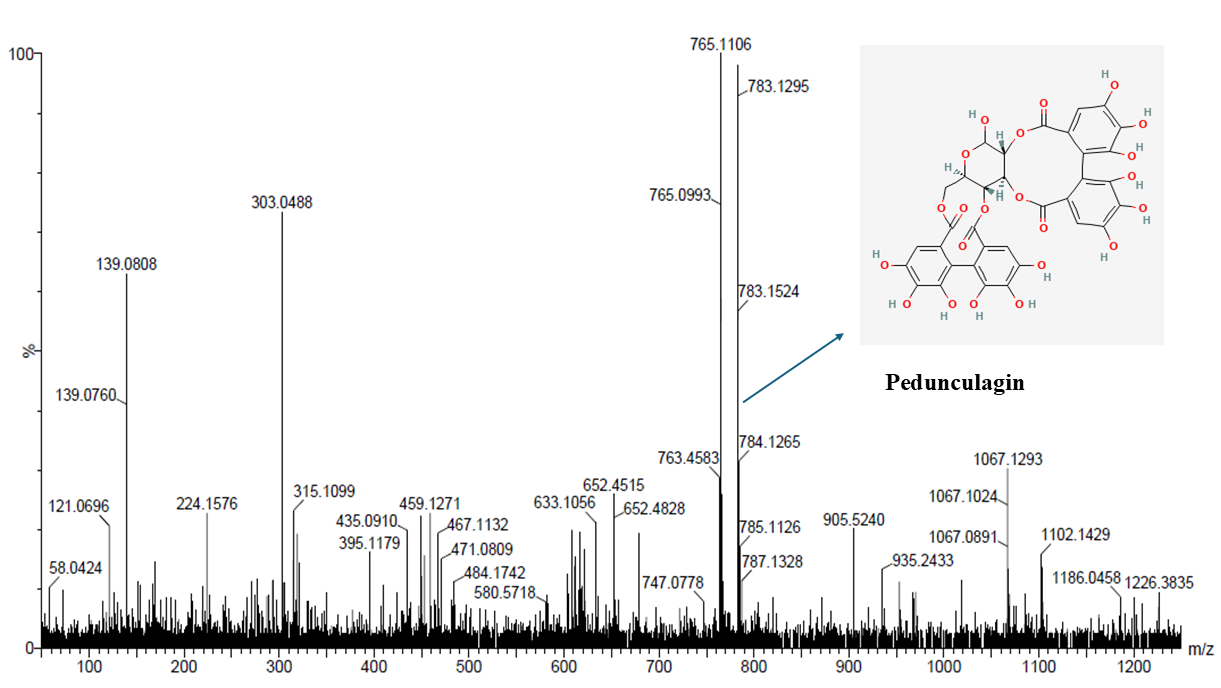
**

Figure 14S: LCMS spectra of Pedunculagin.

**
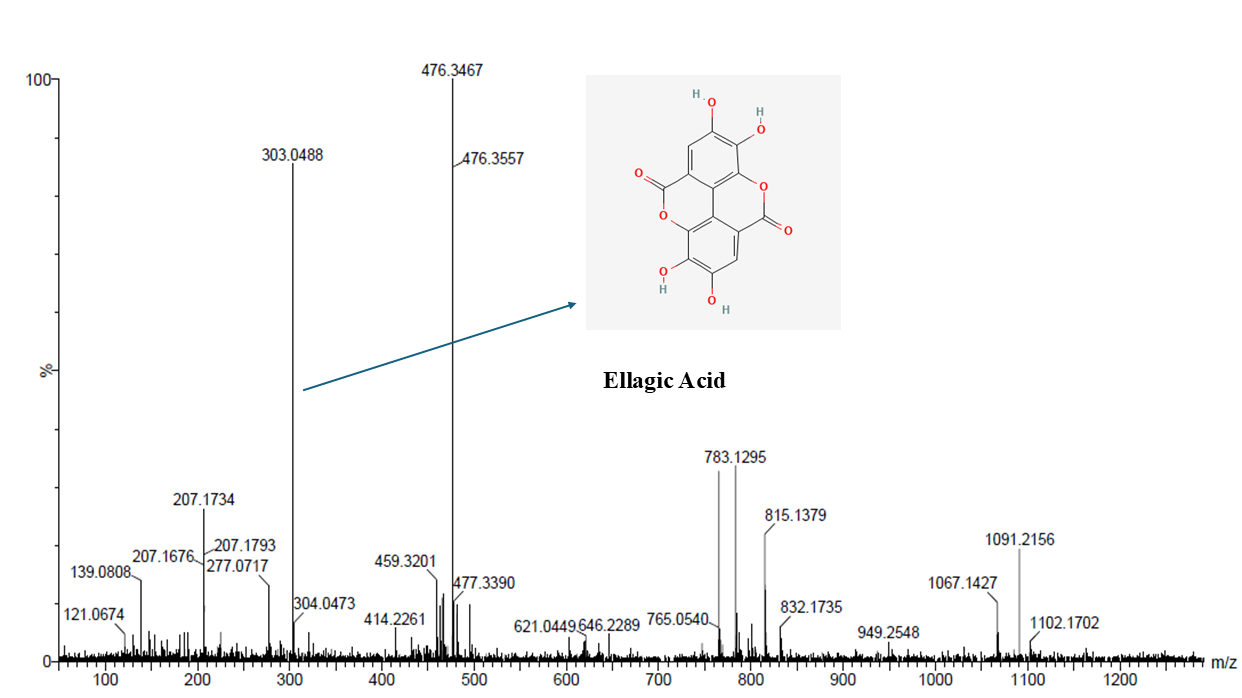
**

Figure 15S: LCMS spectra of Ellagic acid.

**
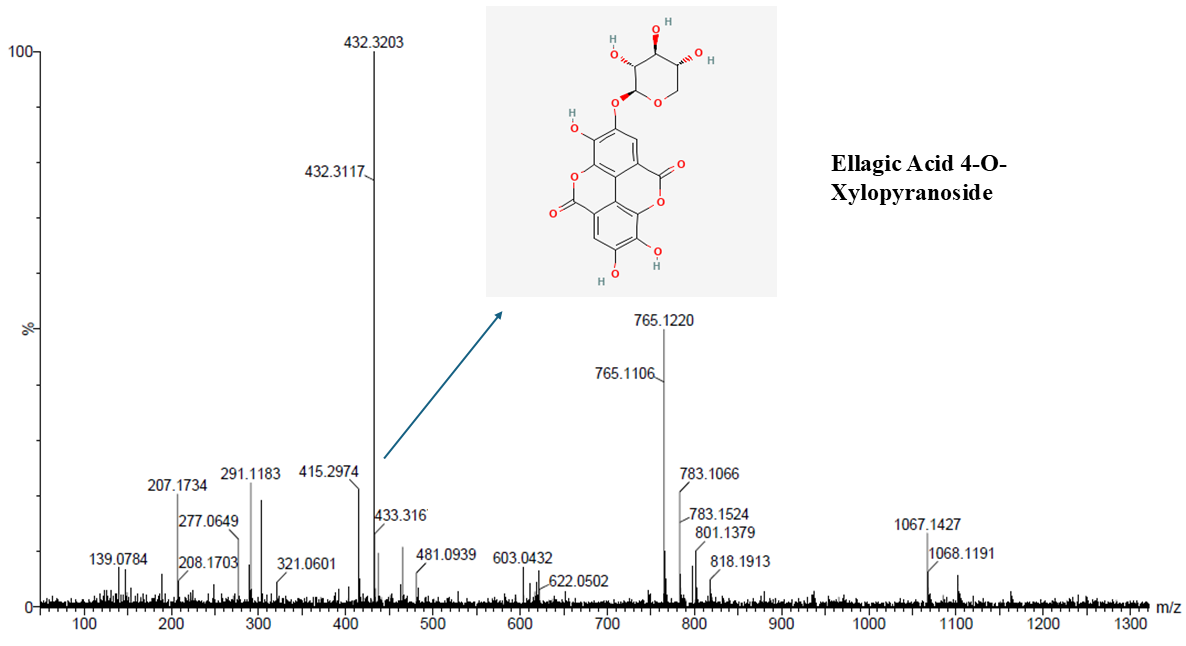
**

Figure 16S: LCMS spectra of Ellagic acid 4-O-Xylopyranoside.

**
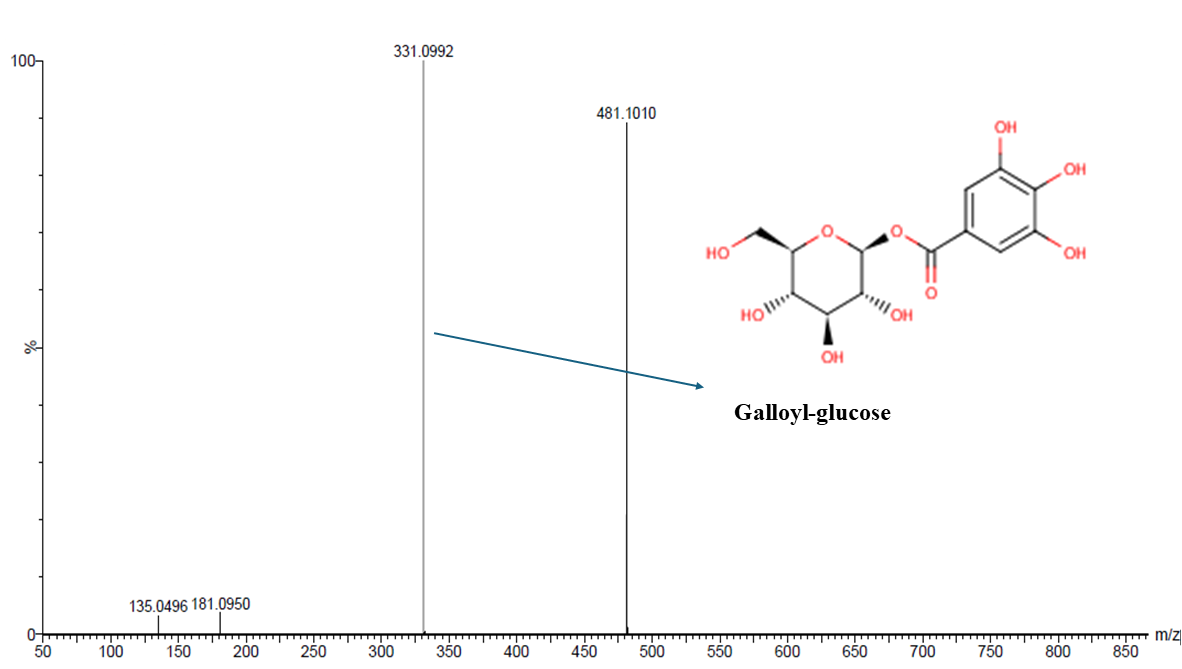
**

Figure 17S: LCMS spectra of Galloyl-glucose.

**
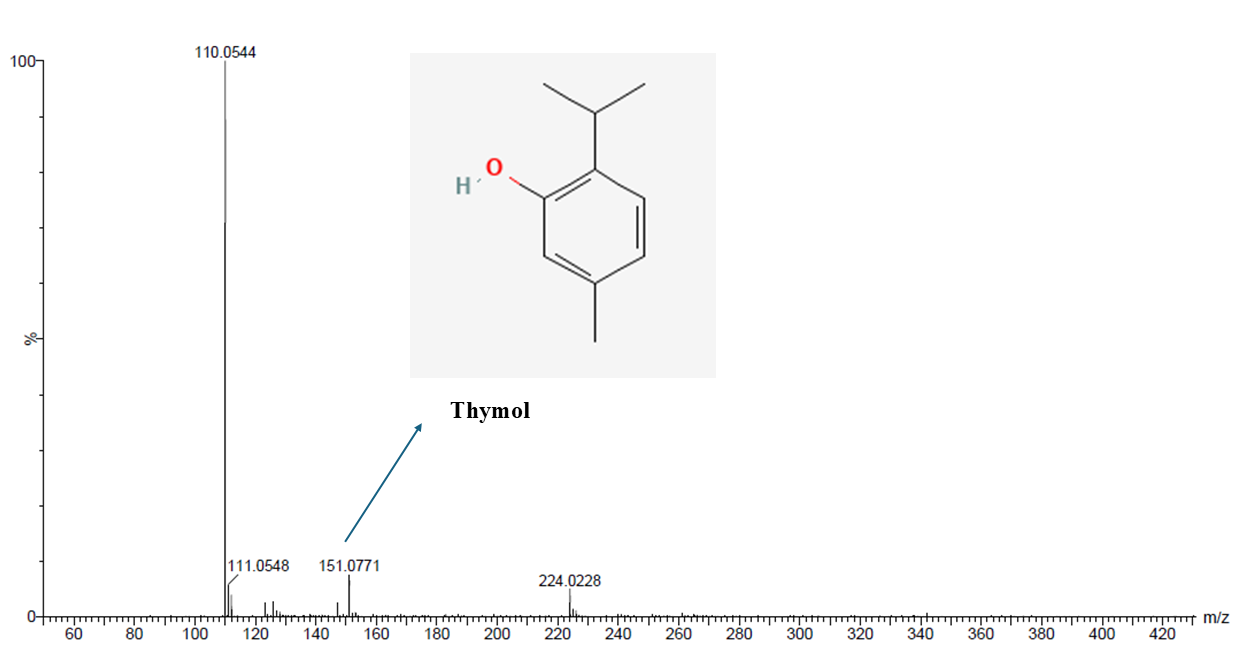
**

Figure 18S: LCMS spectra of Thymol.

**
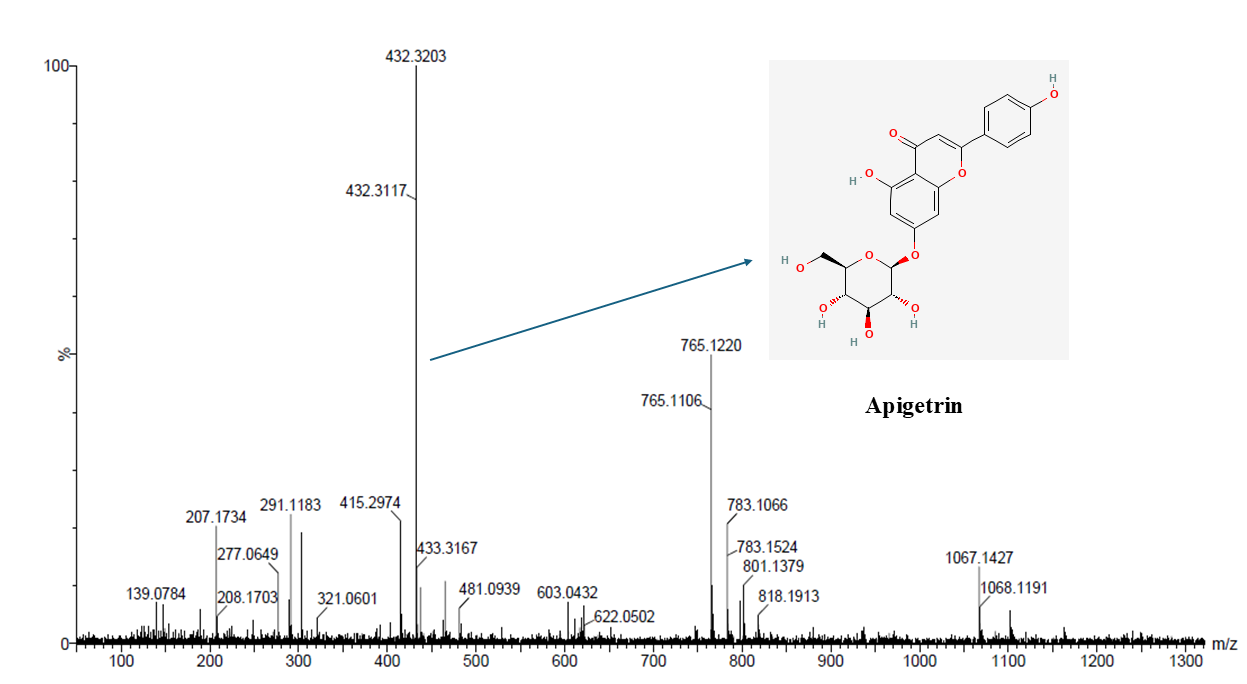
**

Figure 19S: LCMS spectra of Apigetrin.

**
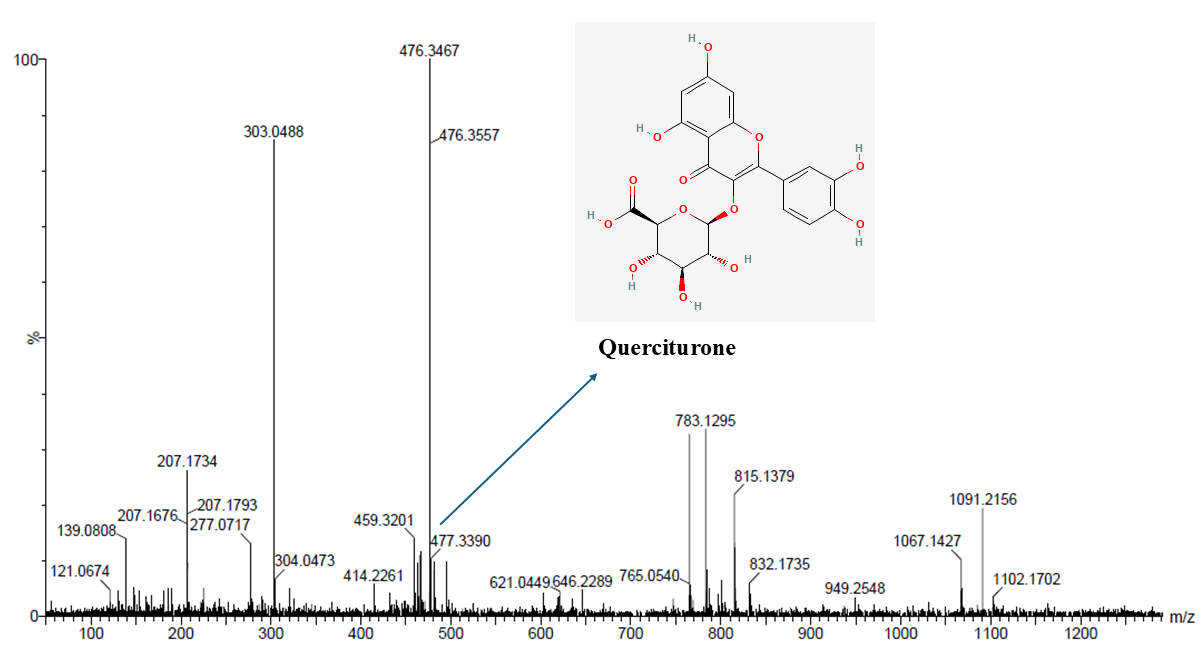
**

Figure 20S: LCMS spectra of Querciturone.

**
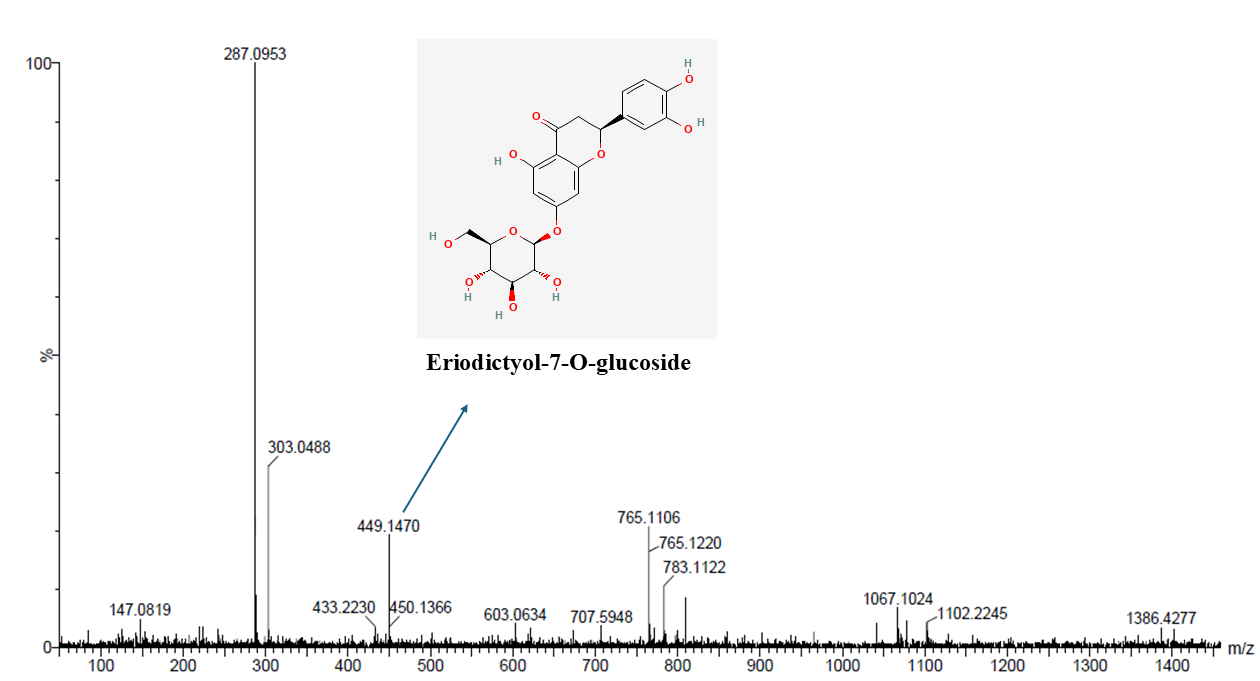
**

Figure 21S: LCMS spectra of Eriodictyol-7-O-glucoside.

**
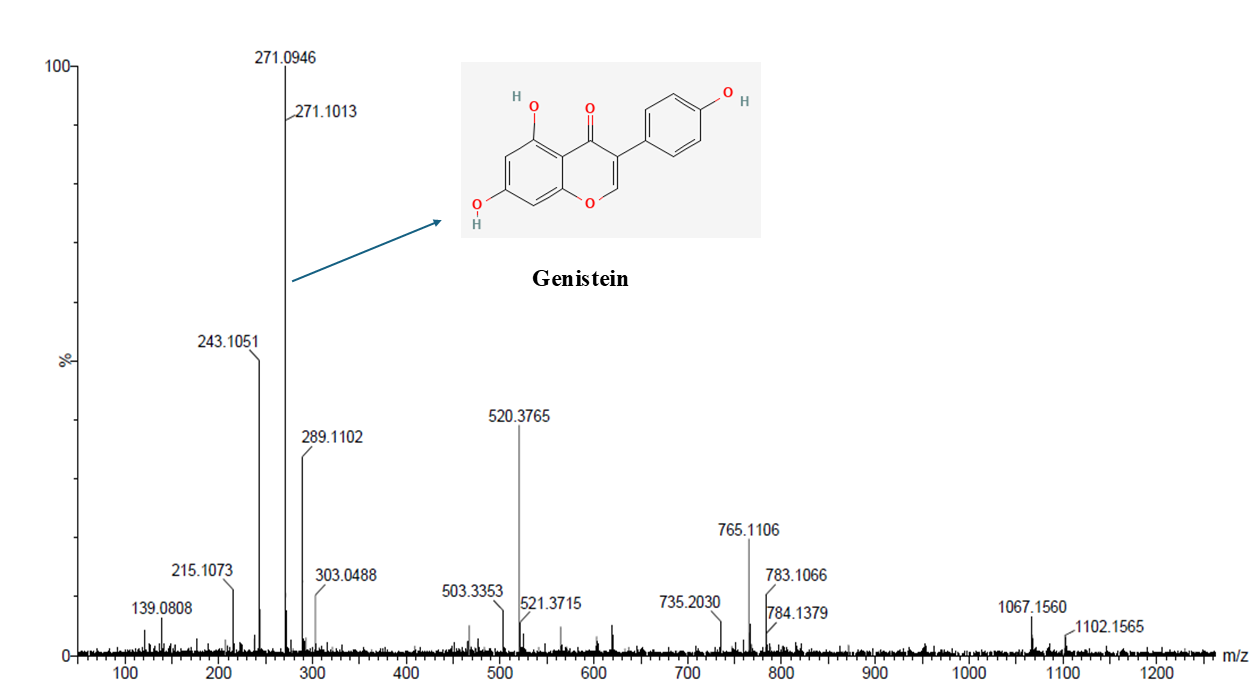
**

Figure 22S: LCMS spectra of Genistein.

**
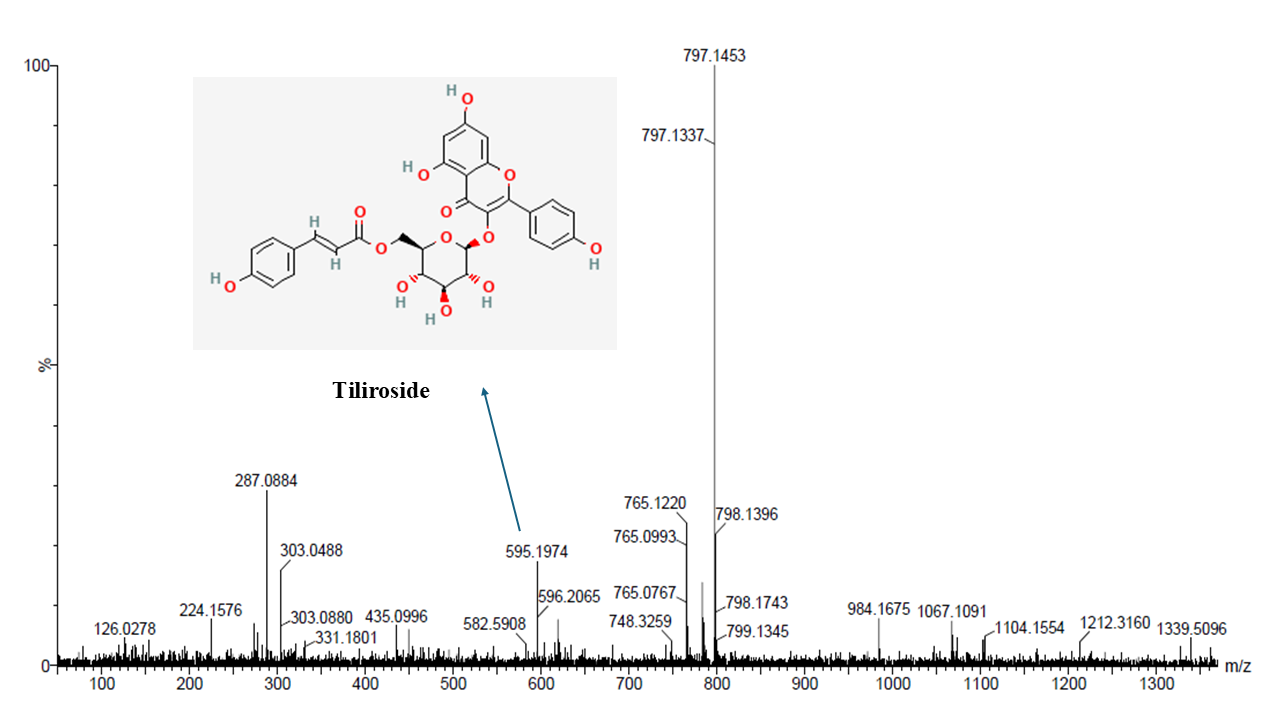
**

Figure 23S: LCMS spectra of Tiliroside.

**
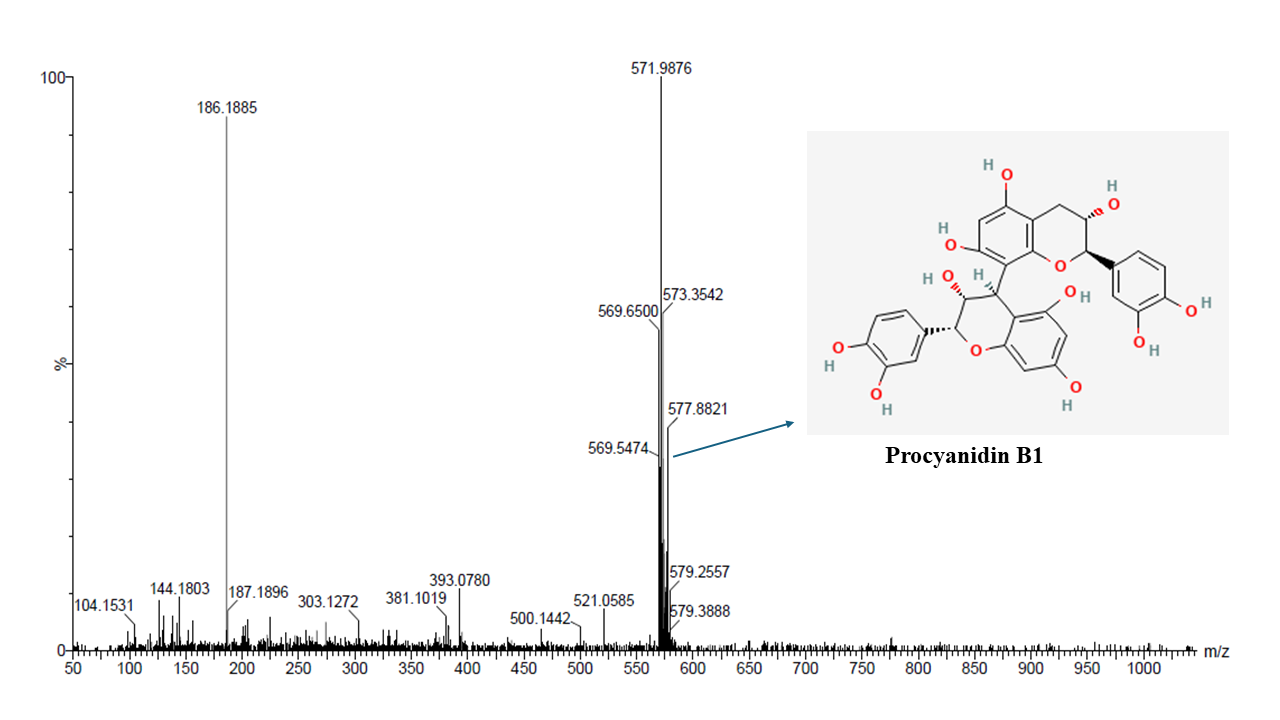
**

Figure 24S: LCMS spectra of Procyanidin B1.

**
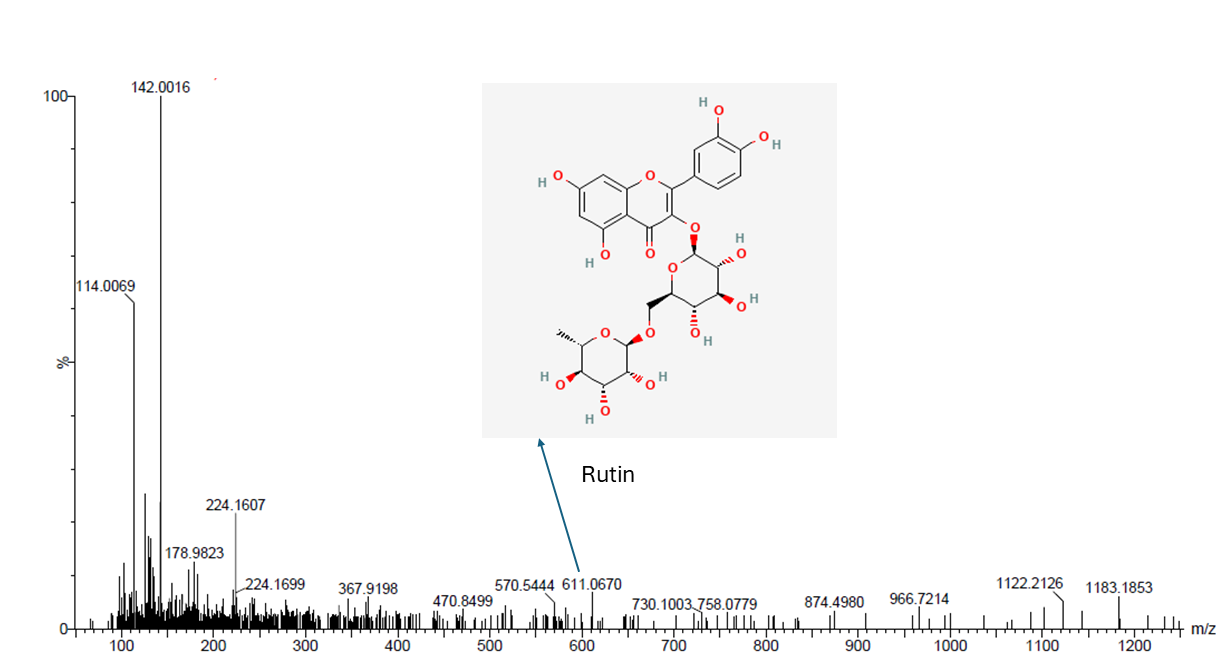
**

Figure 25S: LCMS spectra of Rutin.

**
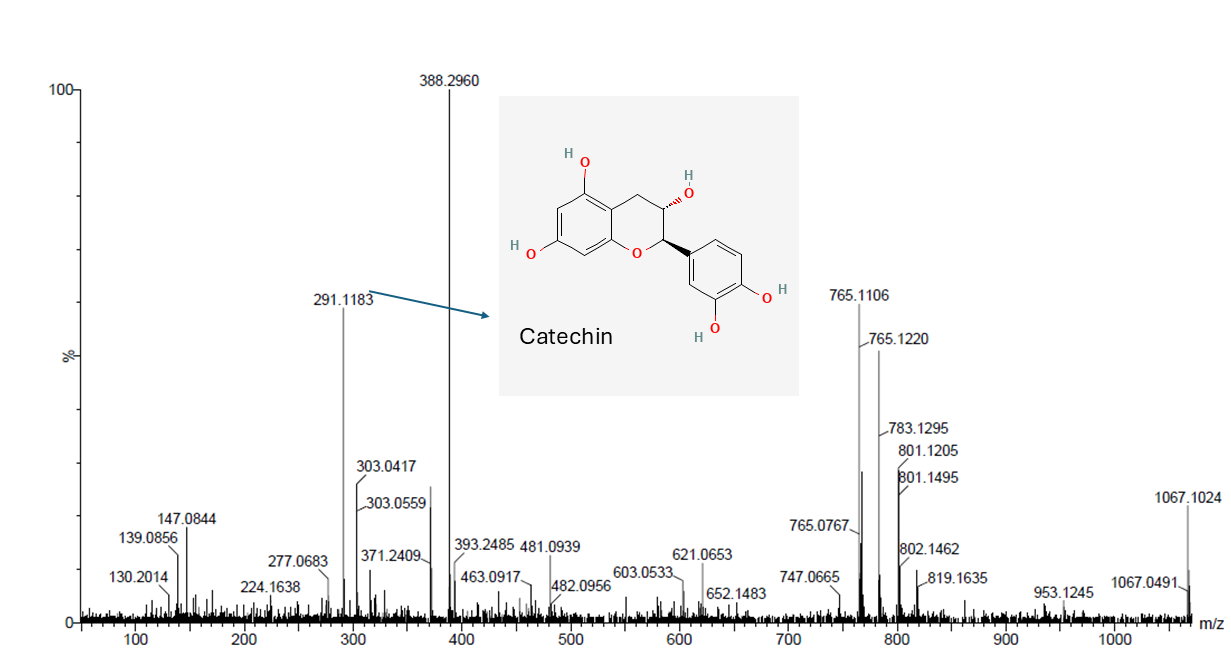
**

Figure 26S: LCMS spectra of Catechin.

**
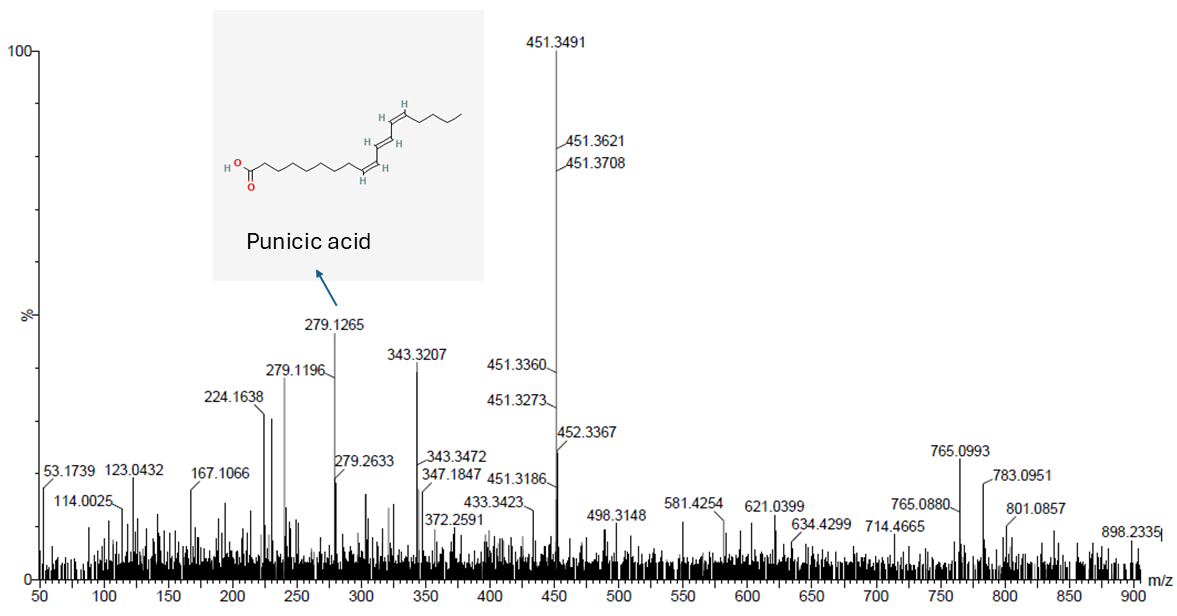
**

Figure 27S: LCMS spectra of Punicic acid.

**
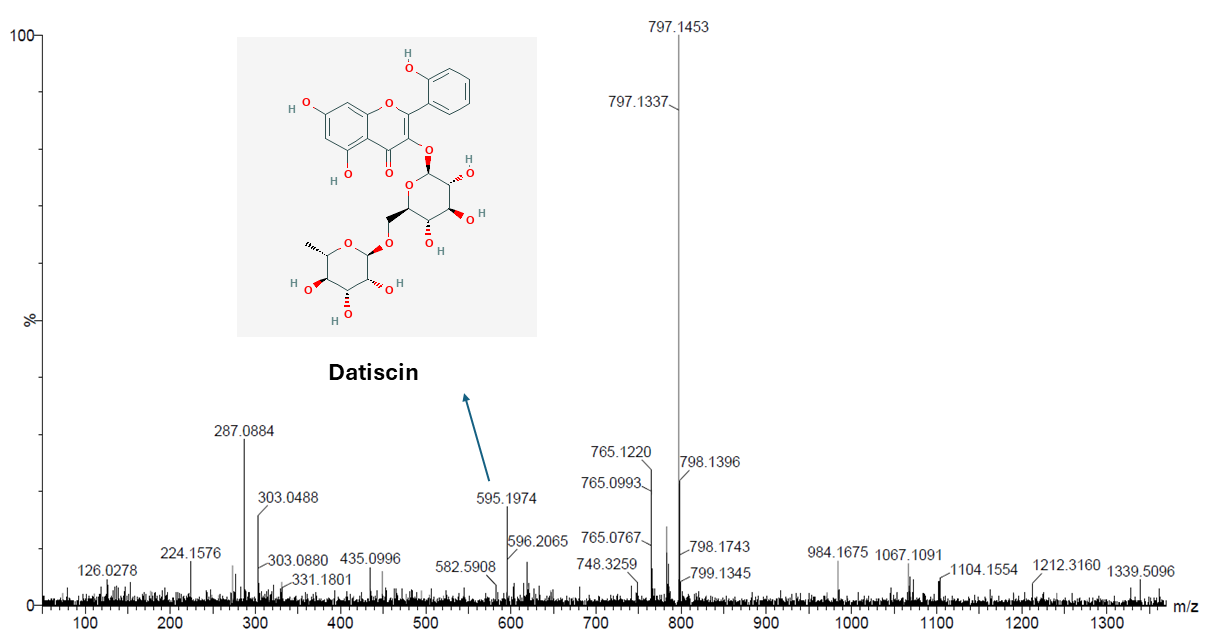
**

Figure 28S: LCMS spectra of Datiscin.

**
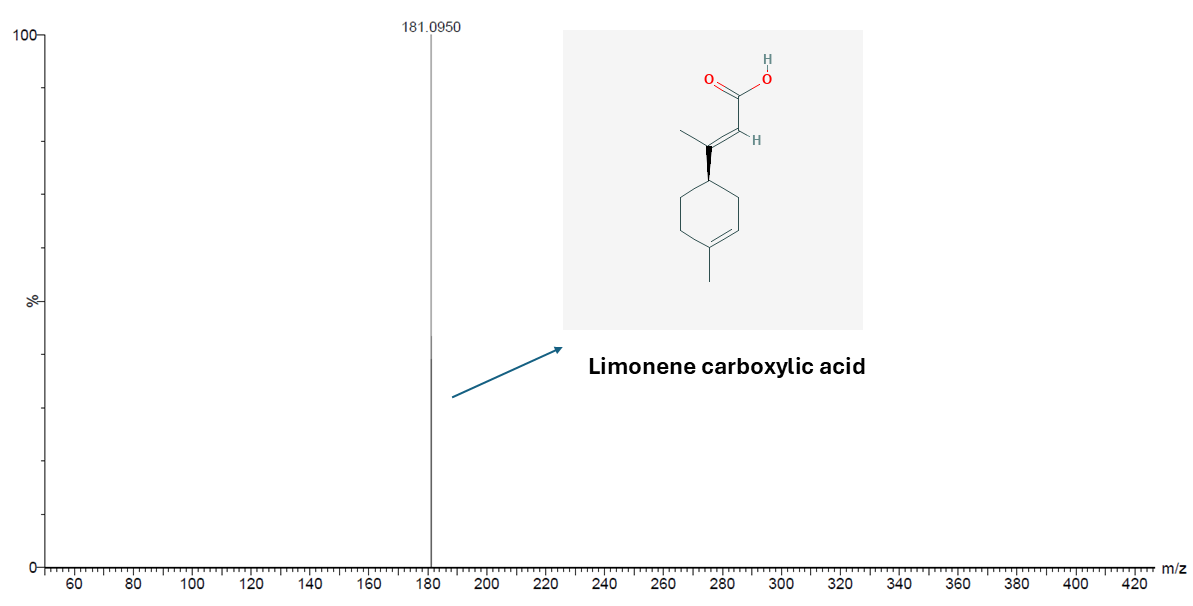
**

Figure 29S: LCMS spectra of Limeonene carboxylic acid.

**
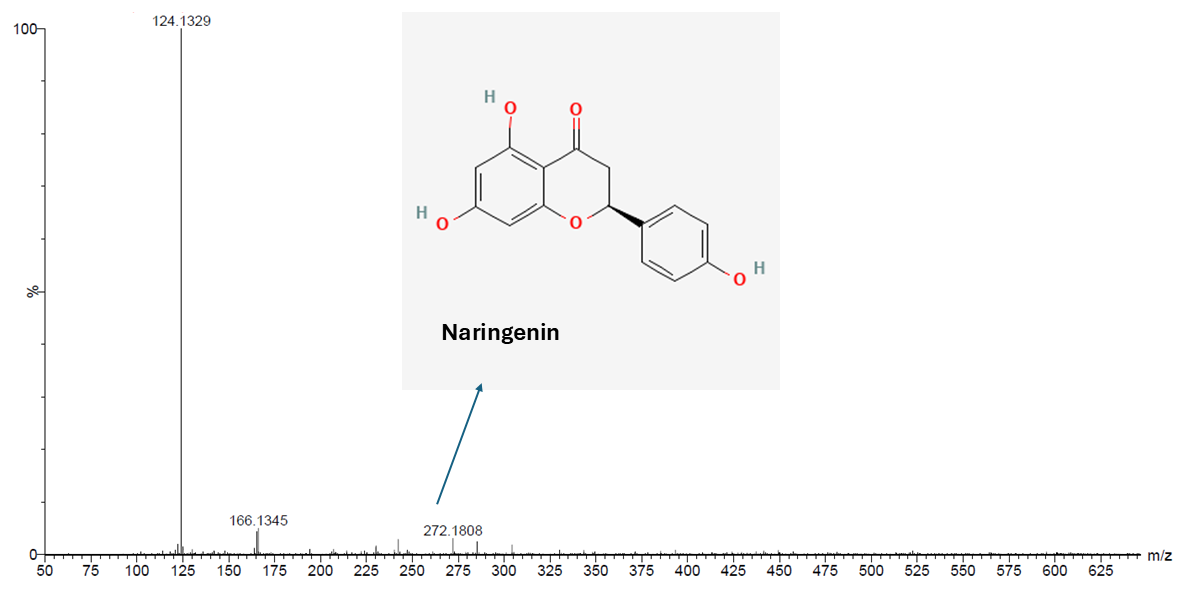
**

Figure 30S: LCMS spectra of Naringenin.

**
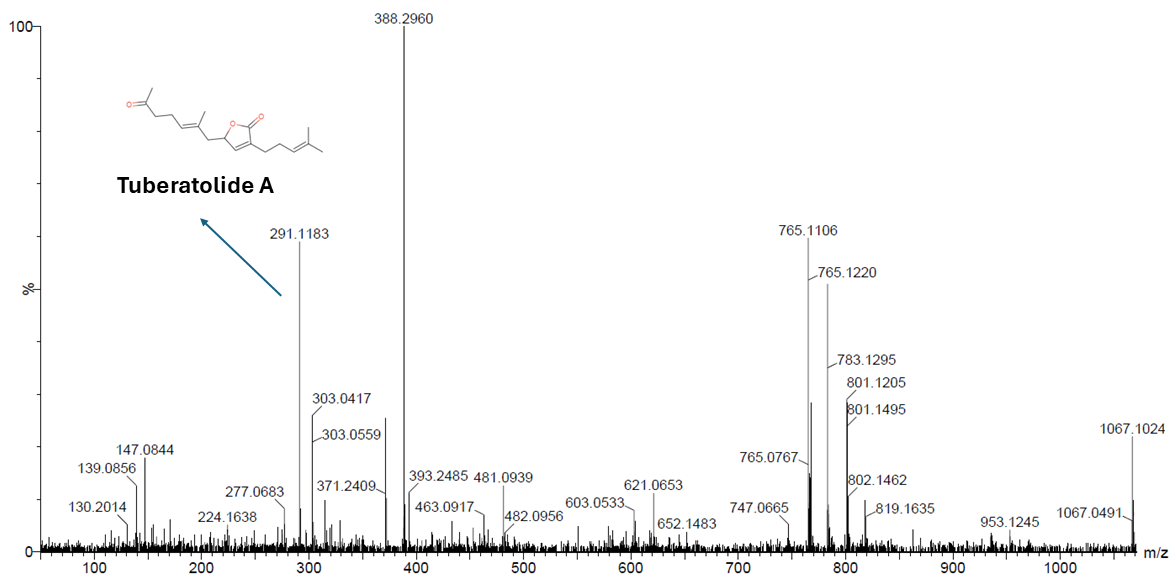
**

Figure 31S: LCMS spectra of Tuberatolide A.

**
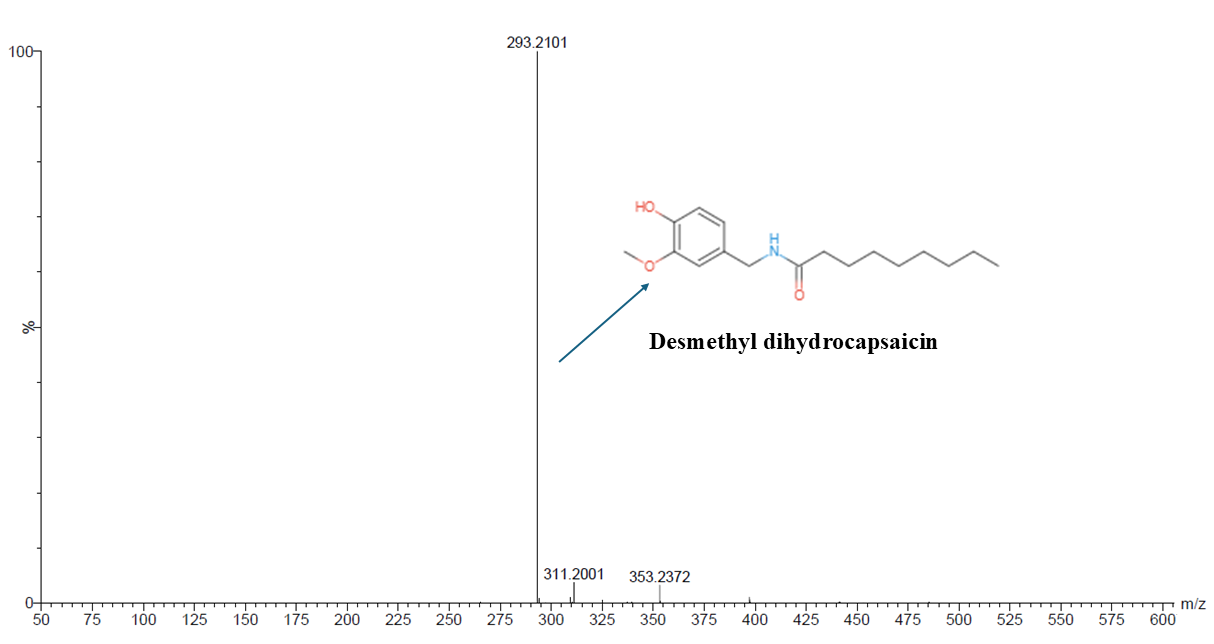
**

Figure 32S: LCMS spectra of Desmethyl dihydrocapsaicin

**
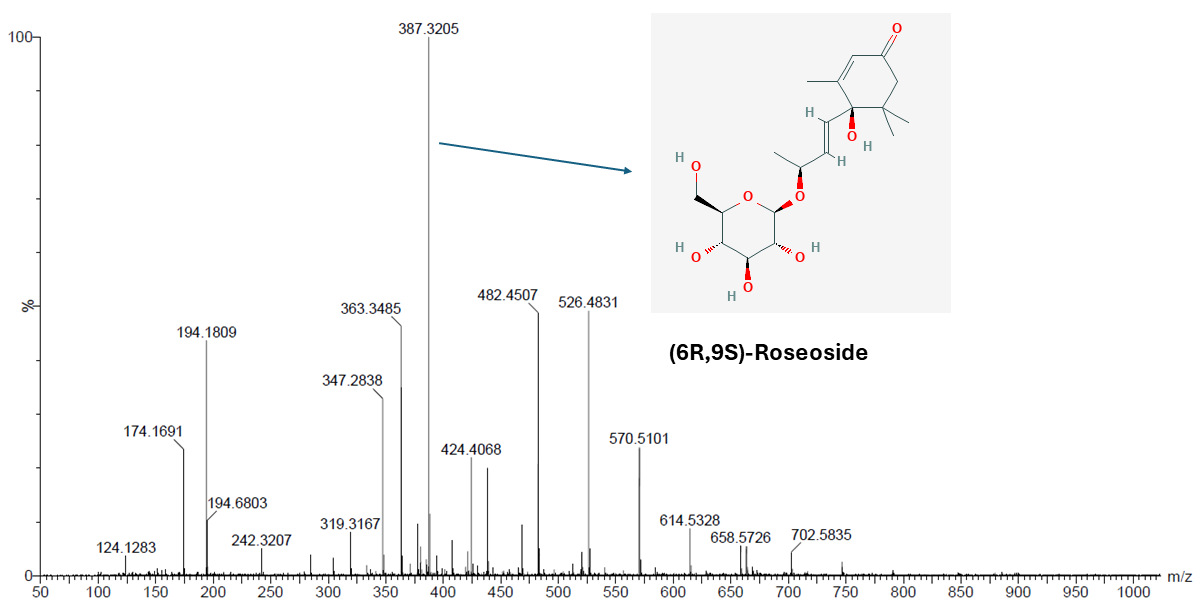
**

Figure 33S: LCMS spectra of (6R, 9S)-Roseoside.

**
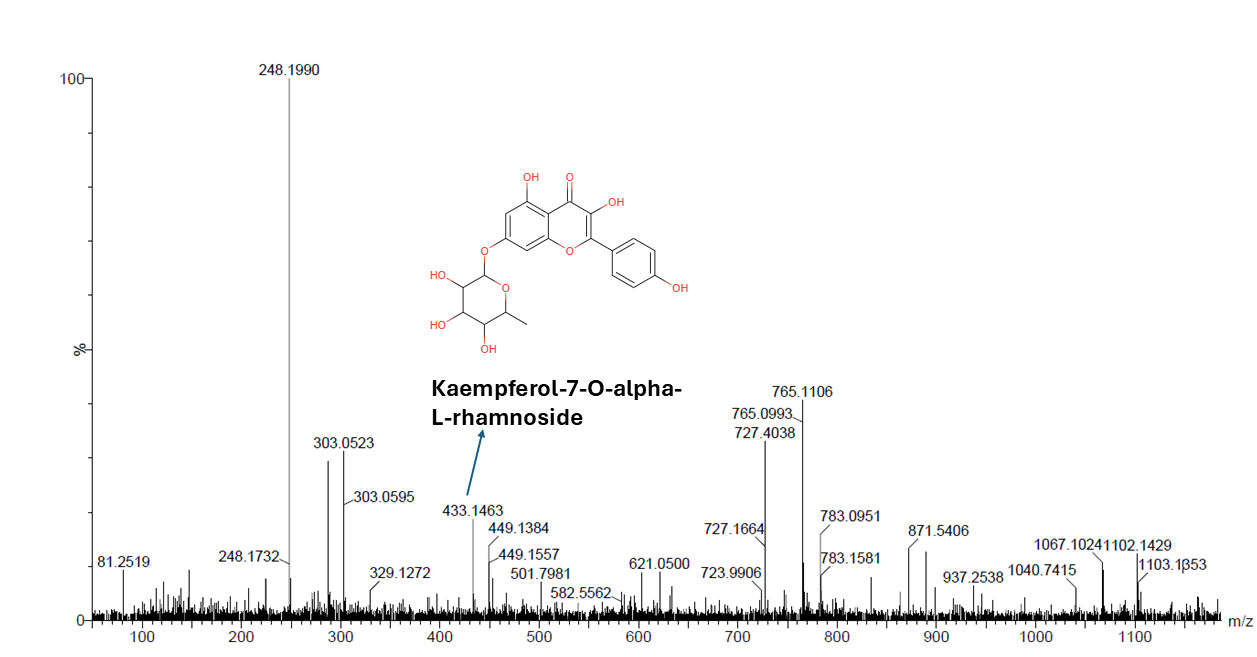
**

Figure 34S: LCMS spectra of Kaempferol-7-O-alpha-L-rhamnoside.

**
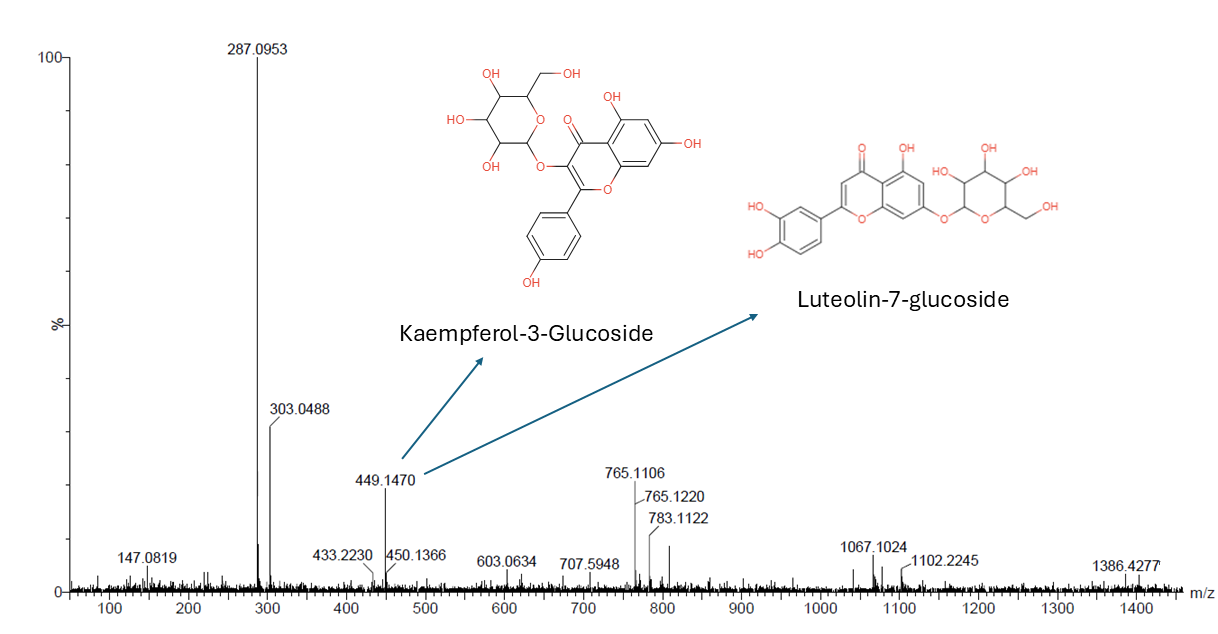
**

Figure 35S: LCMS spectra of Kaempferol-3-Glucoside and Luteolin-7-glucoside.

**
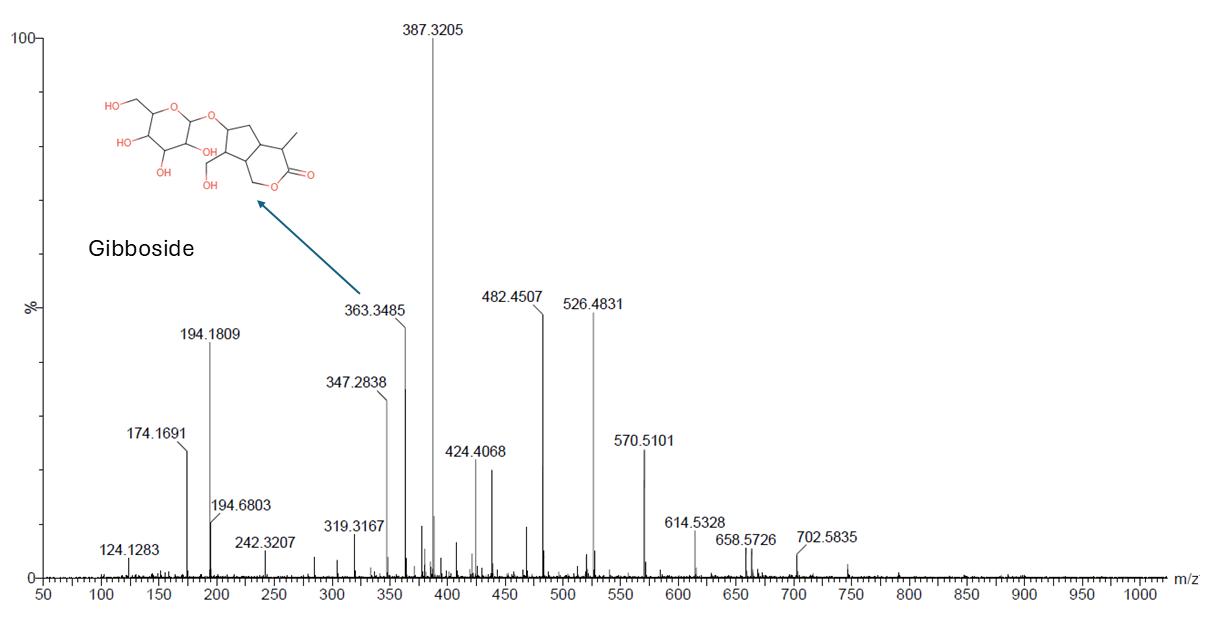
**

Figure 36S: LCMS spectra of Gibboside.

**
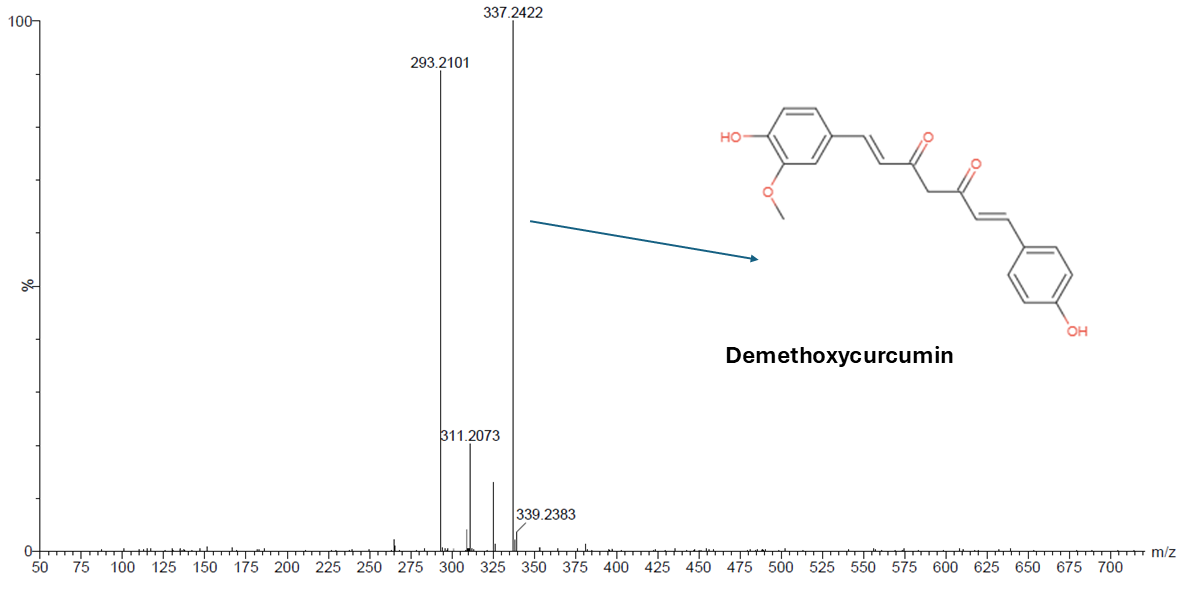
**

Figure 37S: LCMS spectra of Demethoxycurcumin.

**
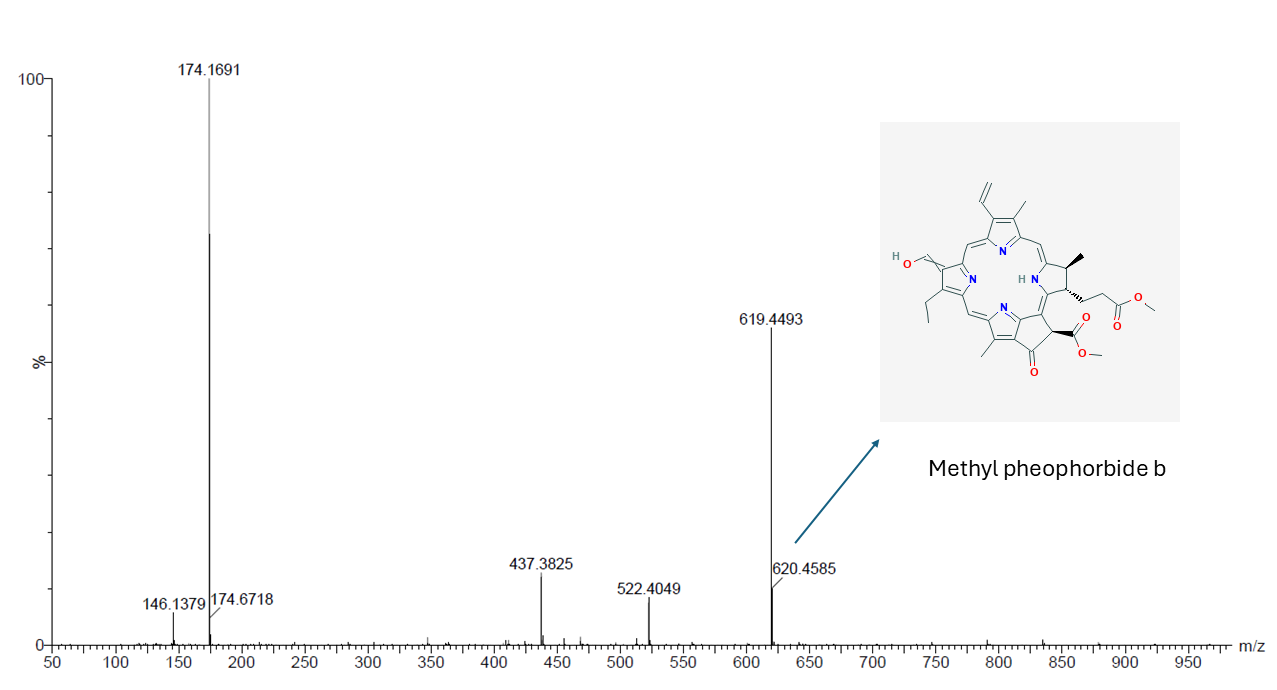
**

Figure 38S: LCMS spectra of Methyl pheophorbide b.

**
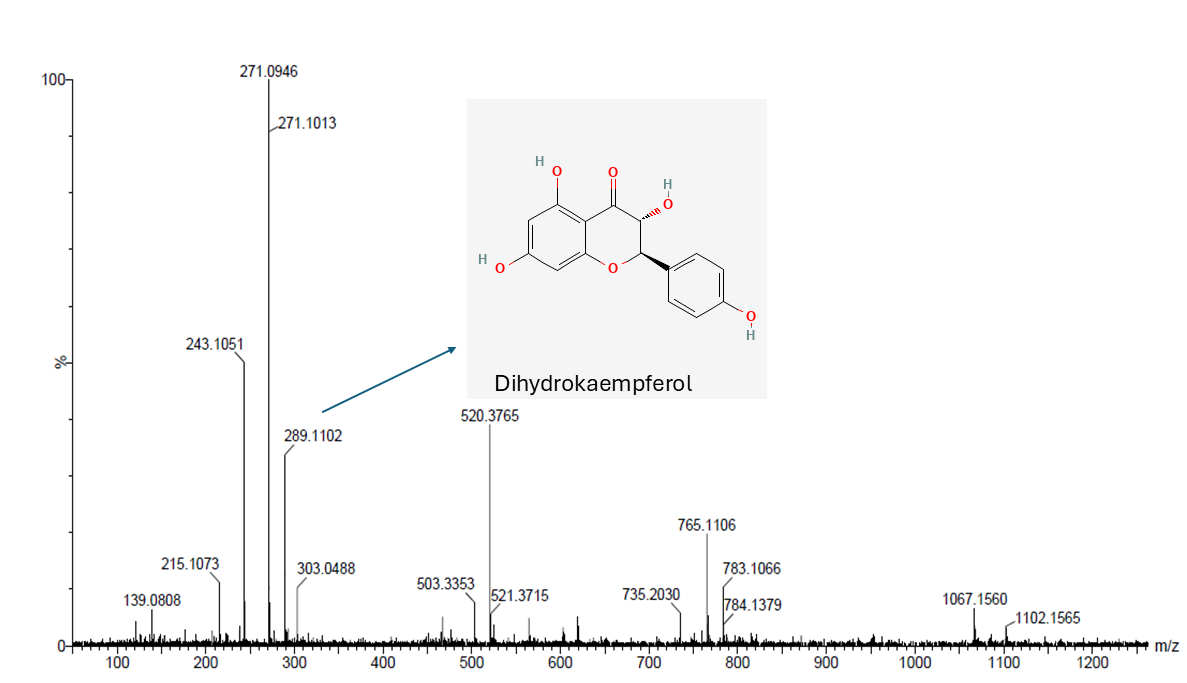
**

Figure 39S: LCMS spectra of Dihydrokaempferol.

**
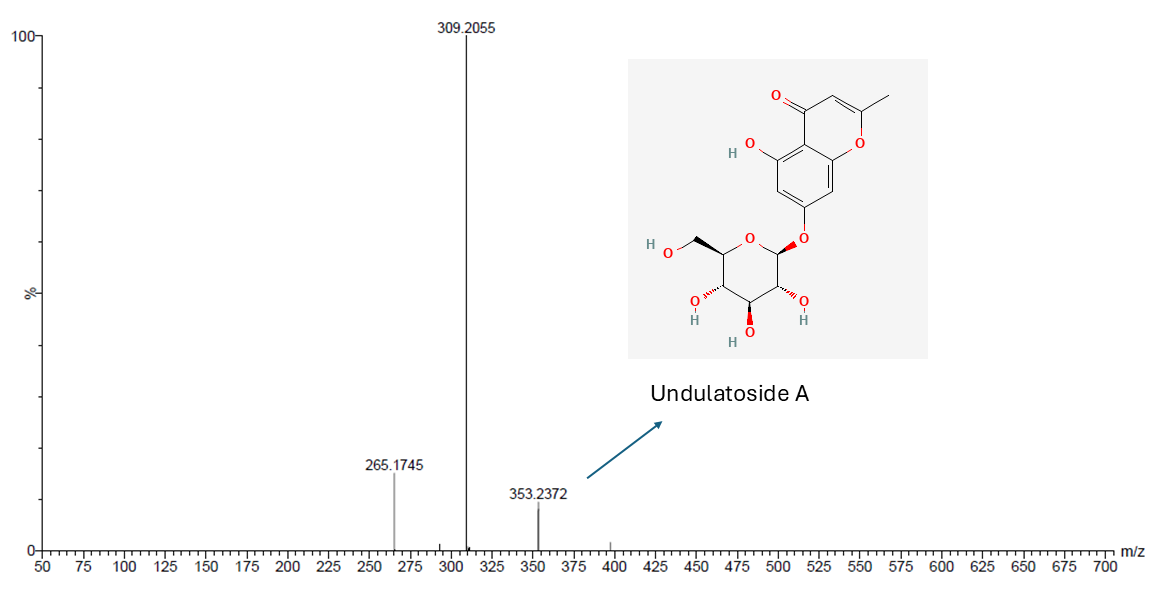
**

Figure 40S: LCMS spectra of Undulatoside A.

**
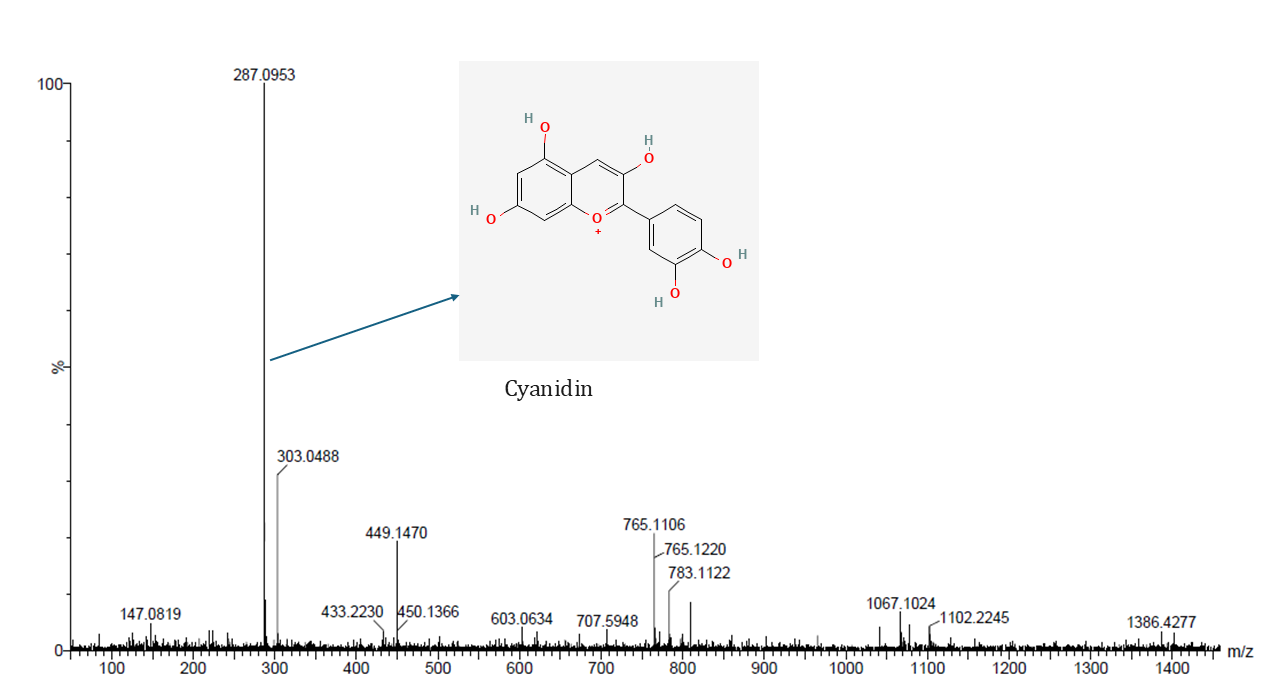
**

Figure 41S: LCMS spectra of Cyanidin.

**
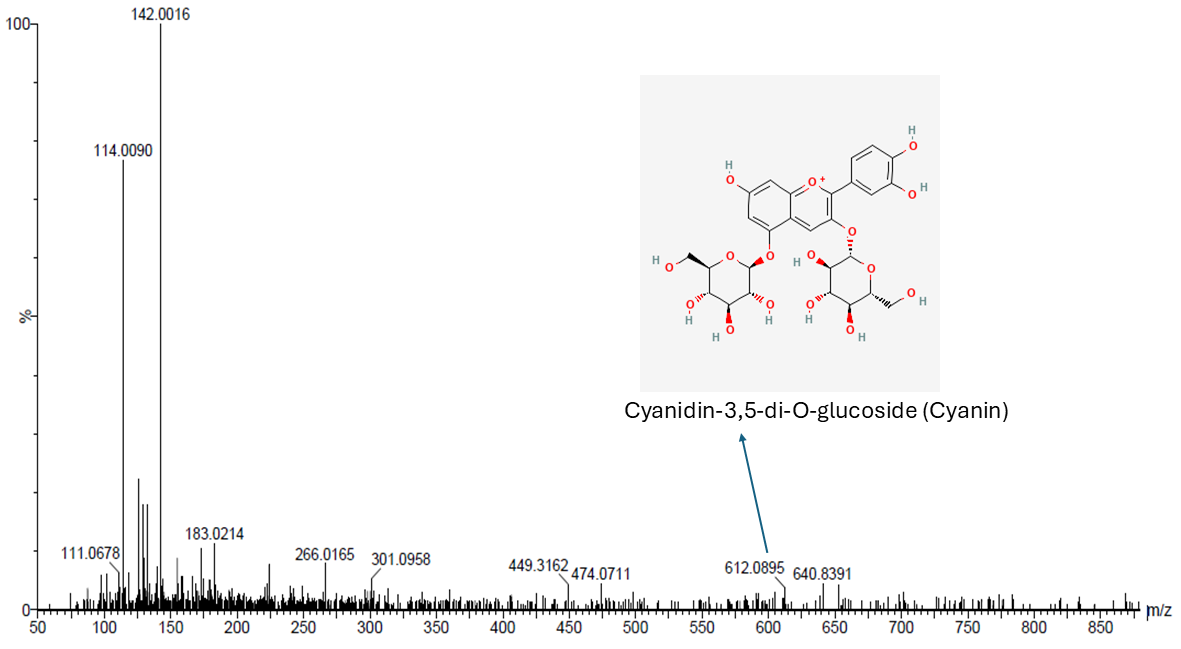
**

Figure 42S: LCMS spectra of Cyanidin-3,5-di-O-glucoside (Cyanin).

**
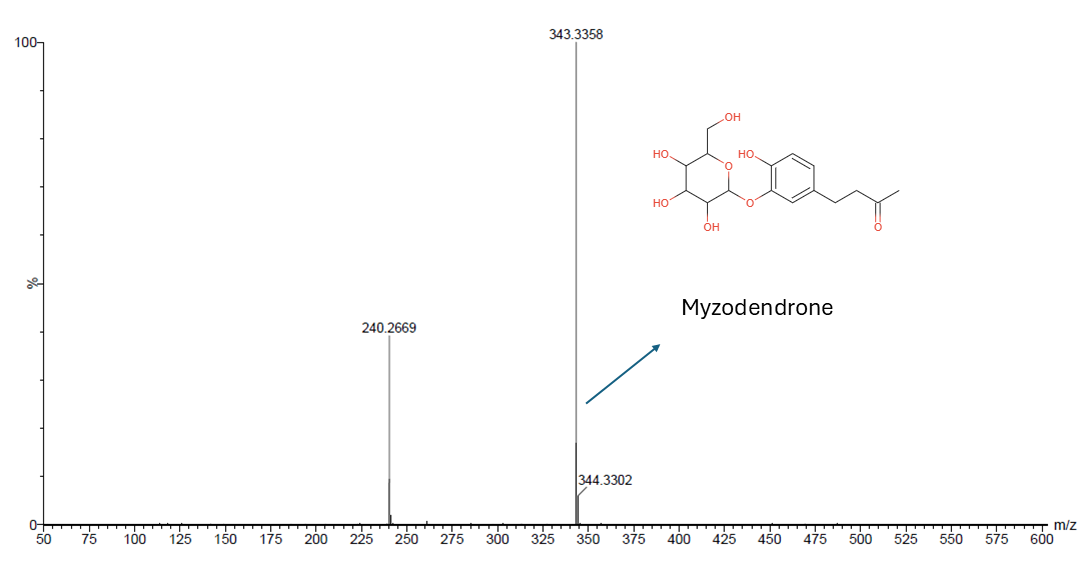
**

Figure 43S: LCMS spectra of Myzodendrone.

**
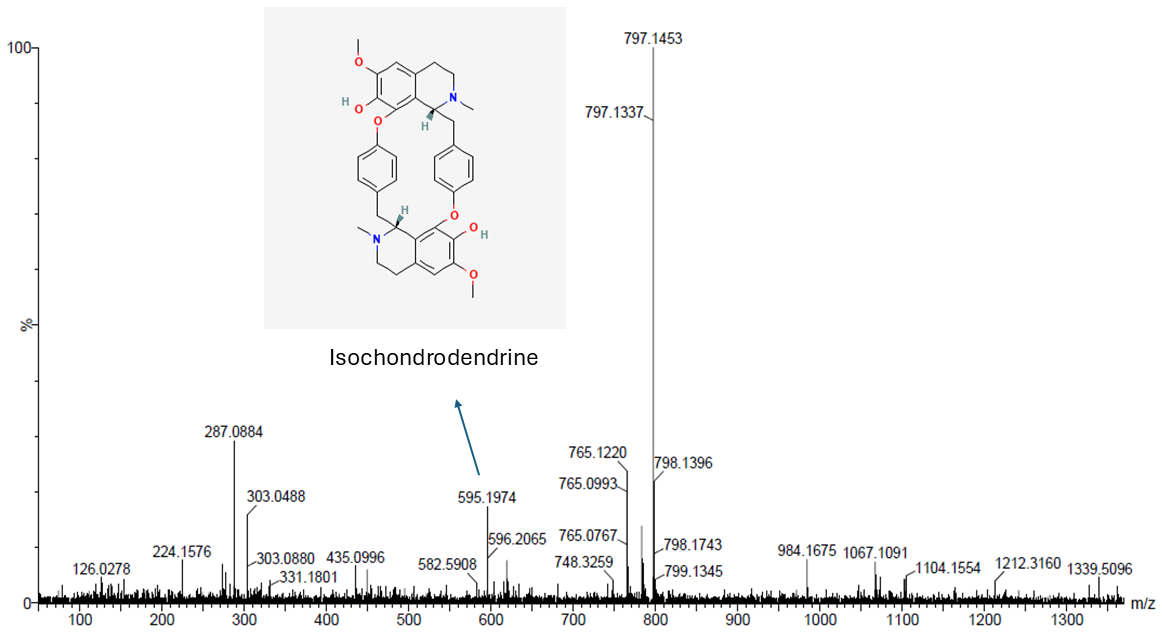
**

Figure 44S: LCMS spectra of Isochondrodendrine.

**
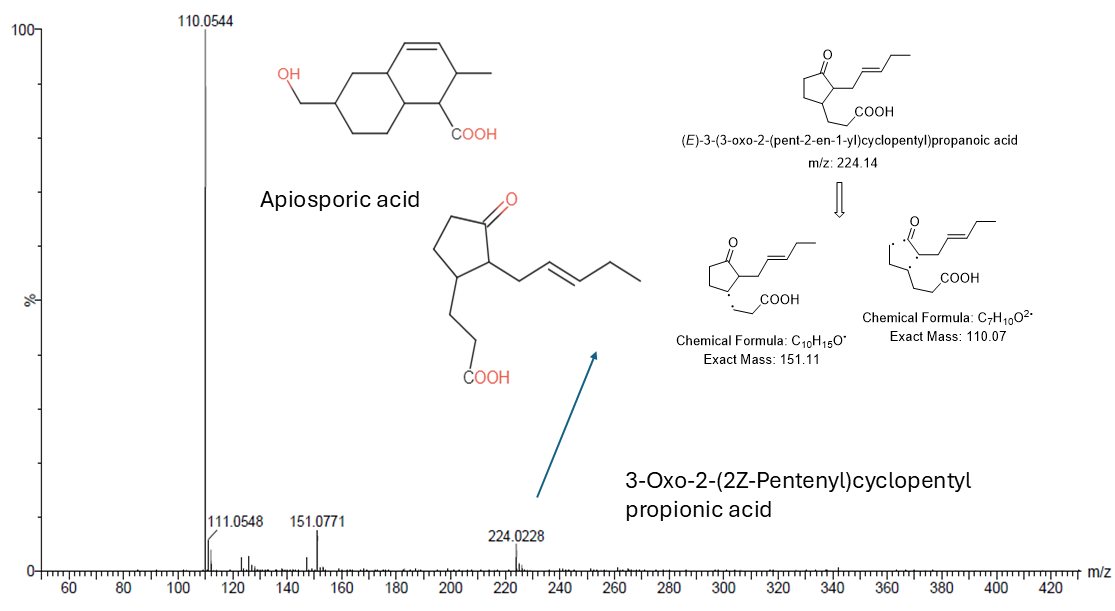
**

Figure 45S: LCMS spectra of Apiosporic acid and 3-Oxo-2-(2Z-Pentenyl)cyclopentyl propionic acid. It also contains fragmentation patters.

**
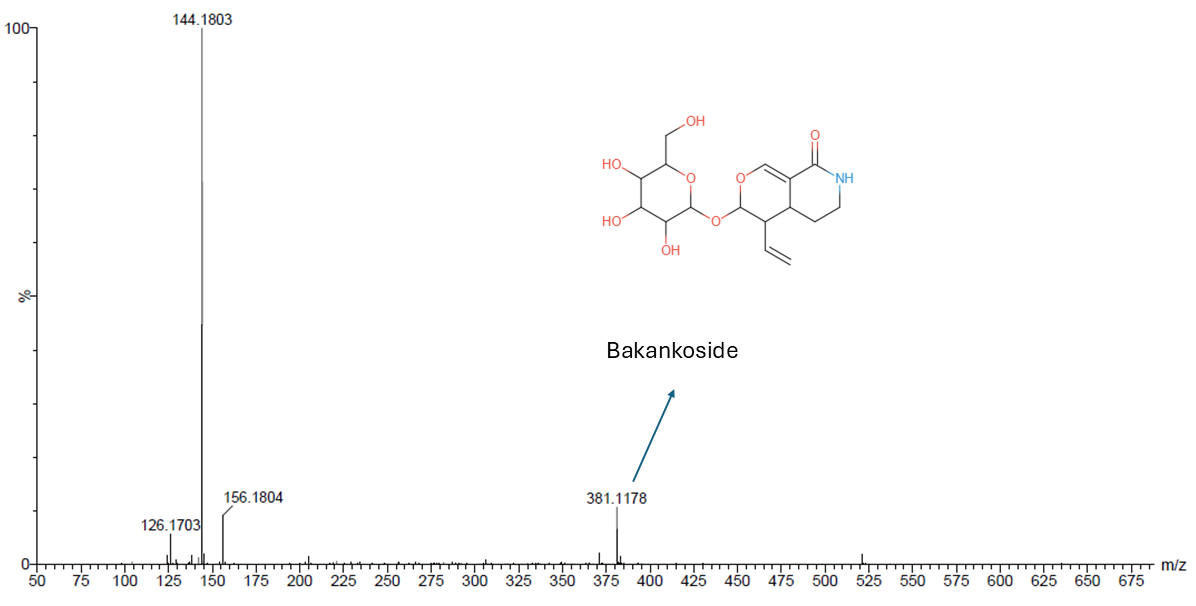
**

Figure 46S: LCMS spectra of Bakankoside.

**
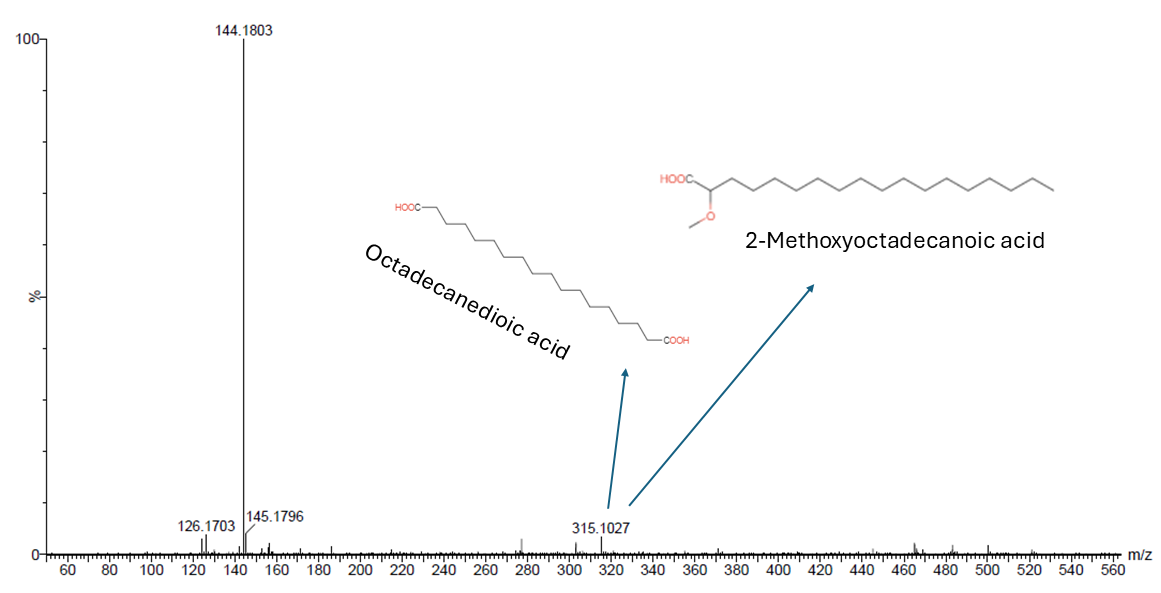
**

Figure 47S: LCMS spectra of Octadecanedioic acid and 2-Methoxyoctadecanoic acid.

**
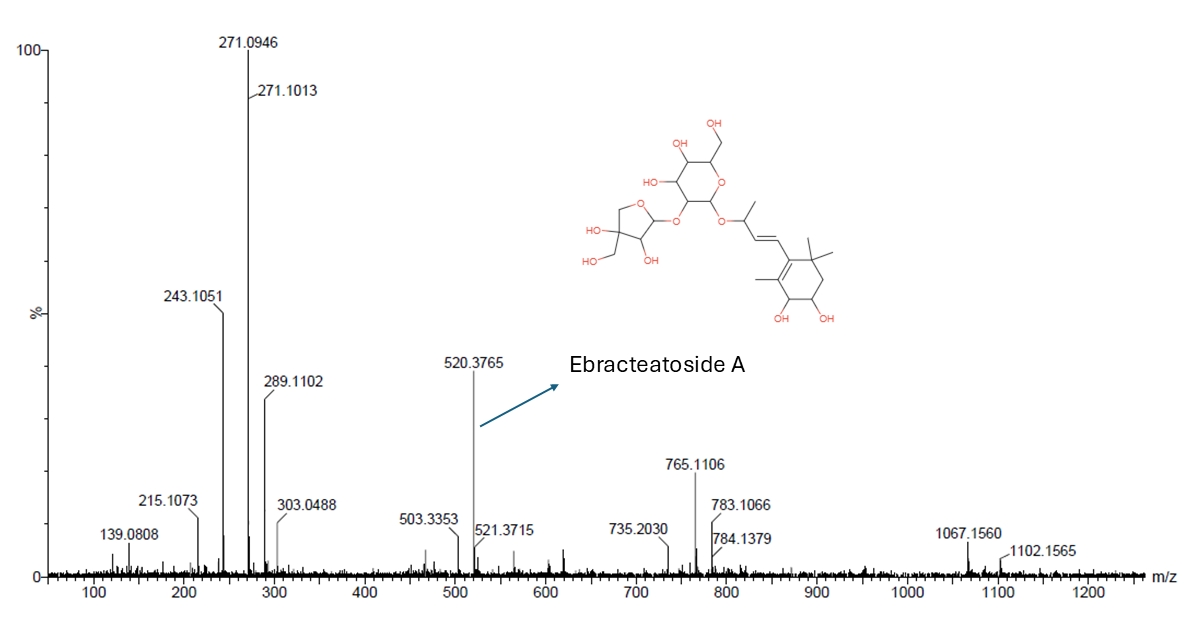
**

Figure 48S: LCMS spectra of Ebracteatoside A.

**
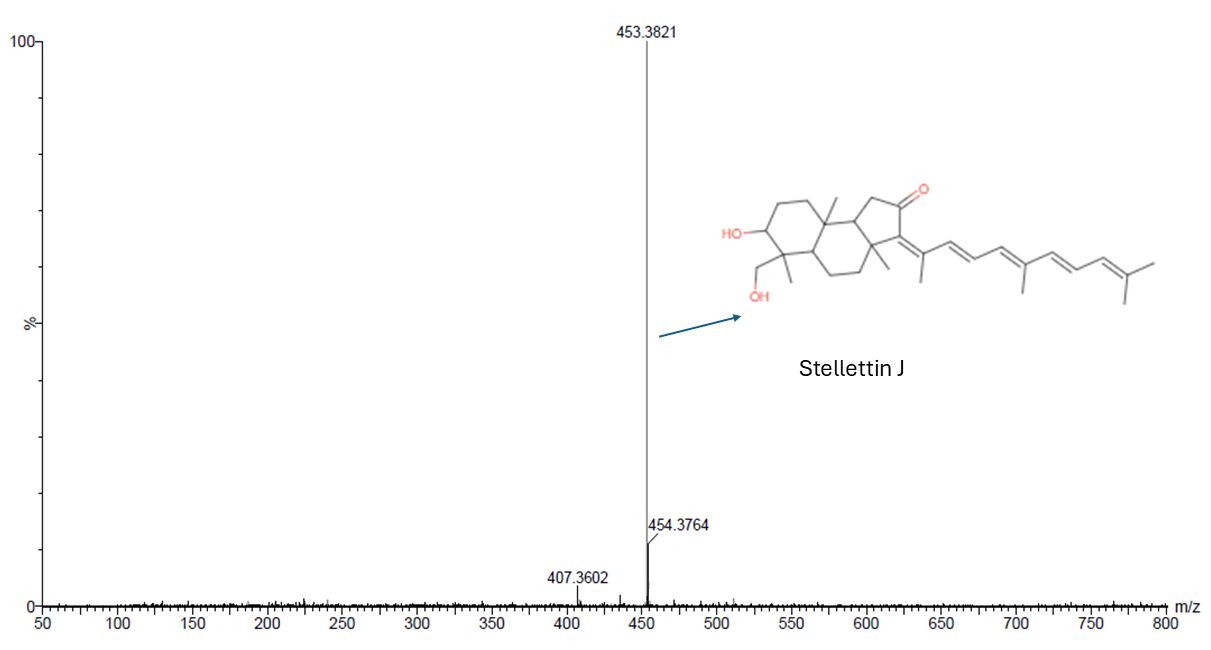
**

Figure 49S: LCMS spectra of Stellettin J.

**
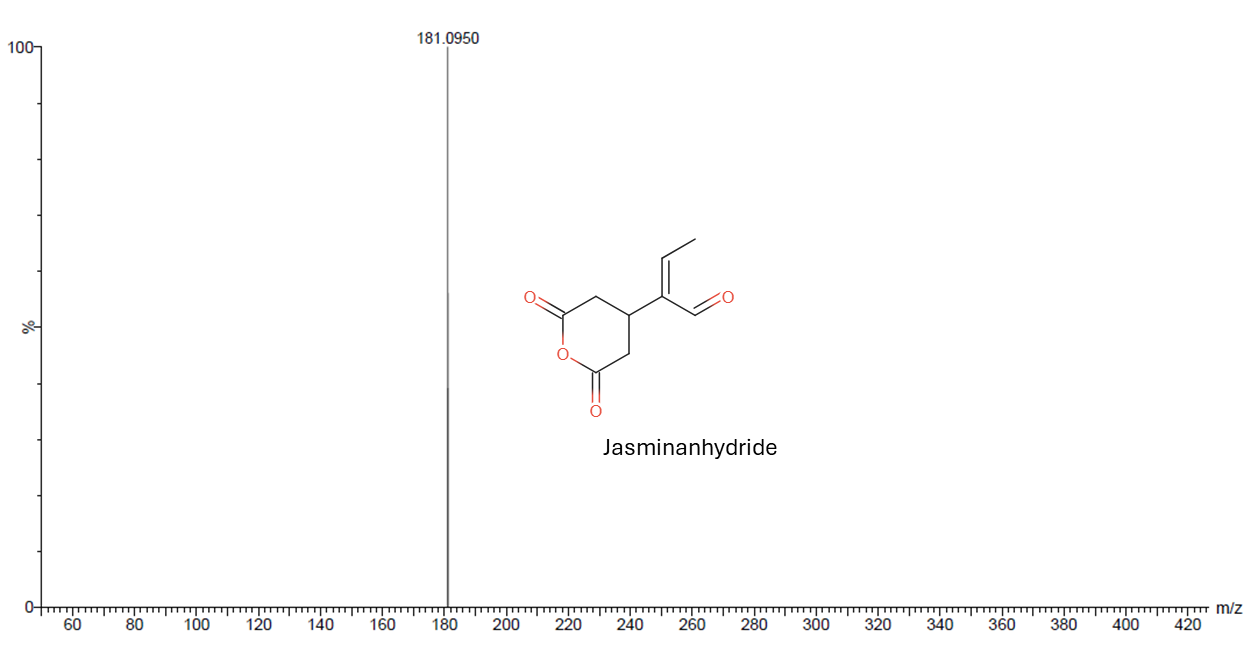
**

Figure 50S: LCMS spectra of Jasminanhydride.

**
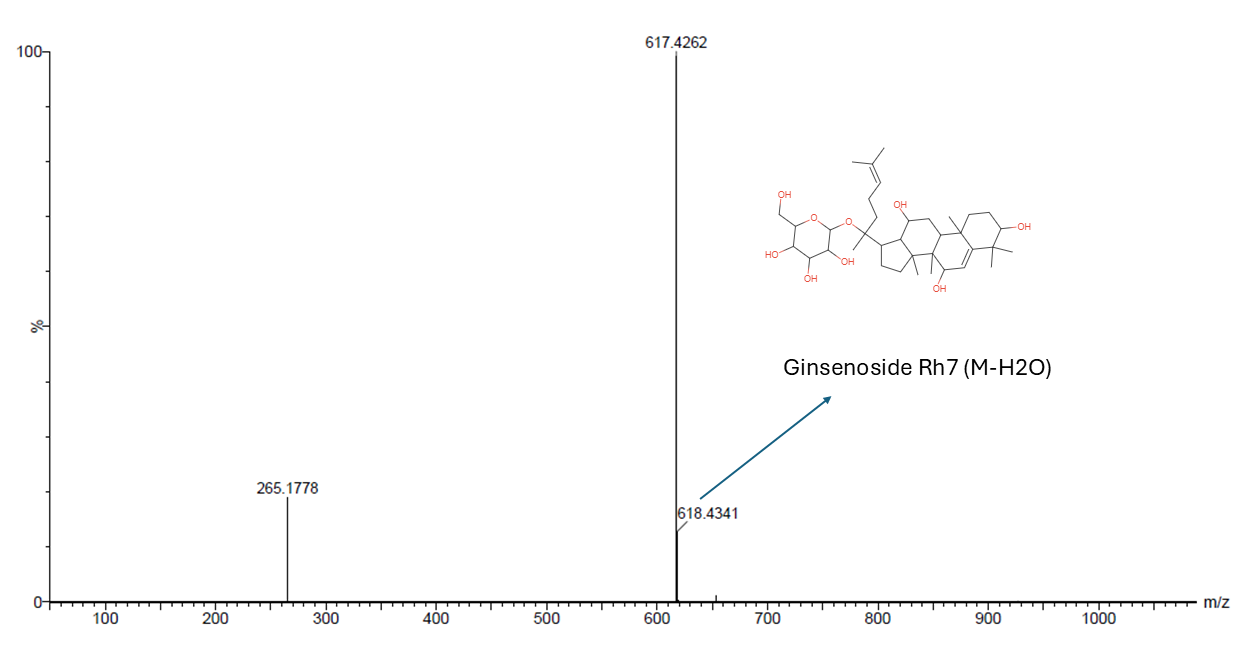
**

Figure 51S: LCMS spectra of Ginsenoside Rh7.

**
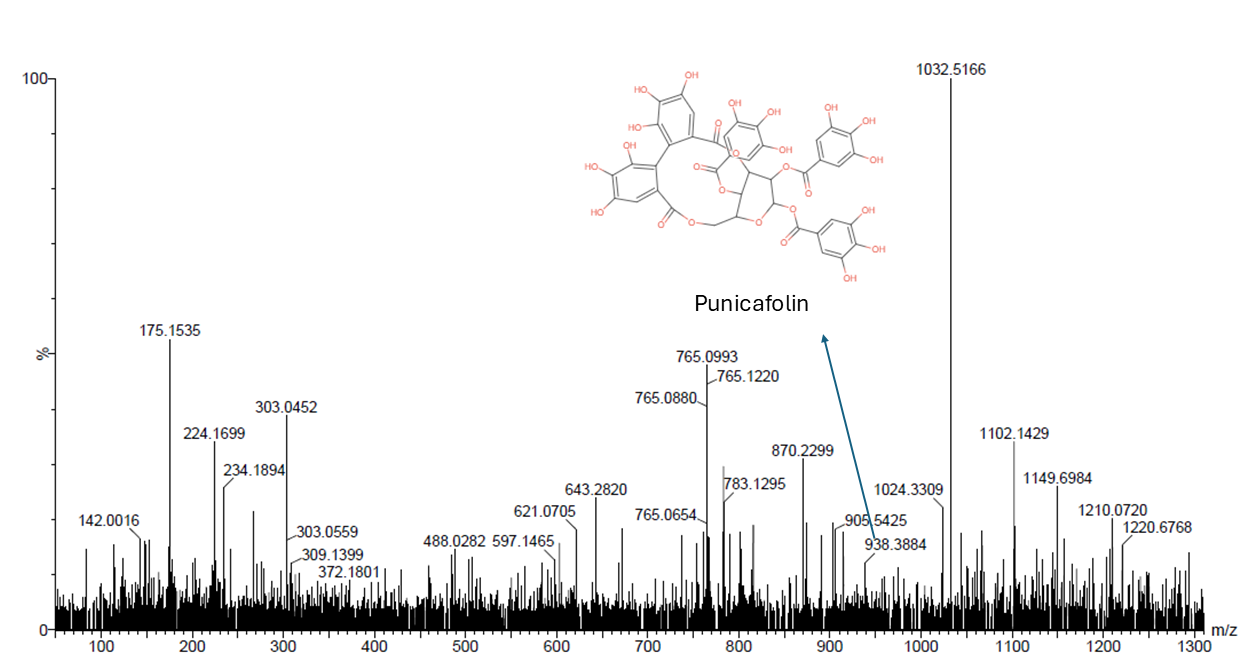
**

Figure 52S: LCMS spectra of Punicafolin.

**
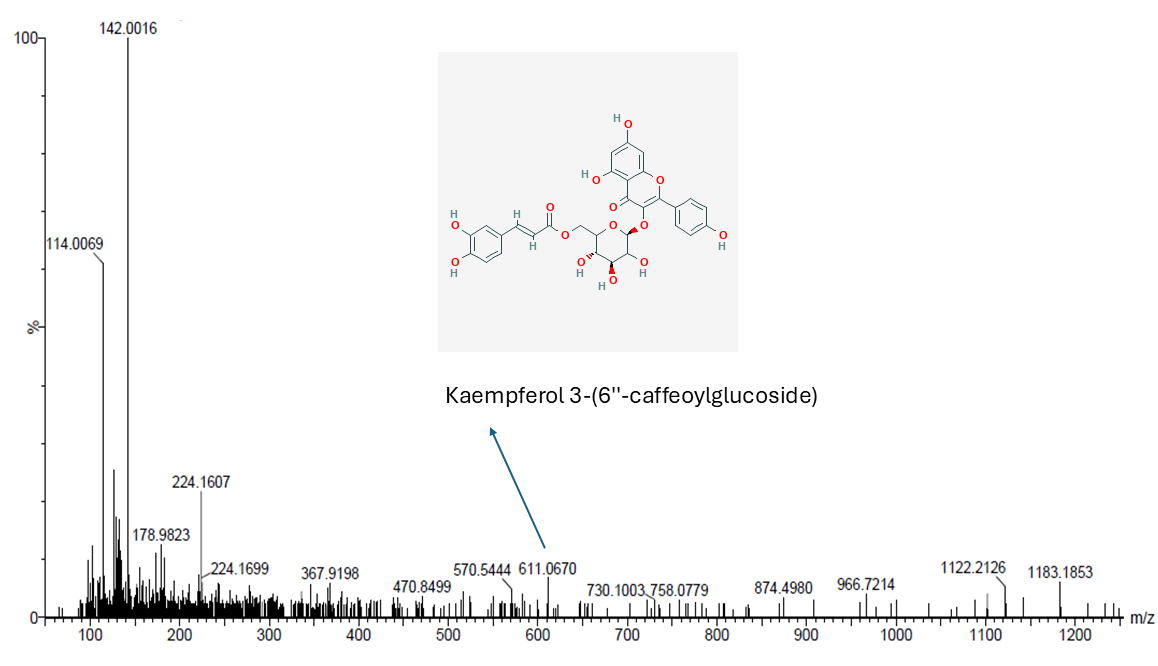
**

Figure 53S: LCMS spectra of Kaempferol 3-(6”-caffeoylglucoside).

**
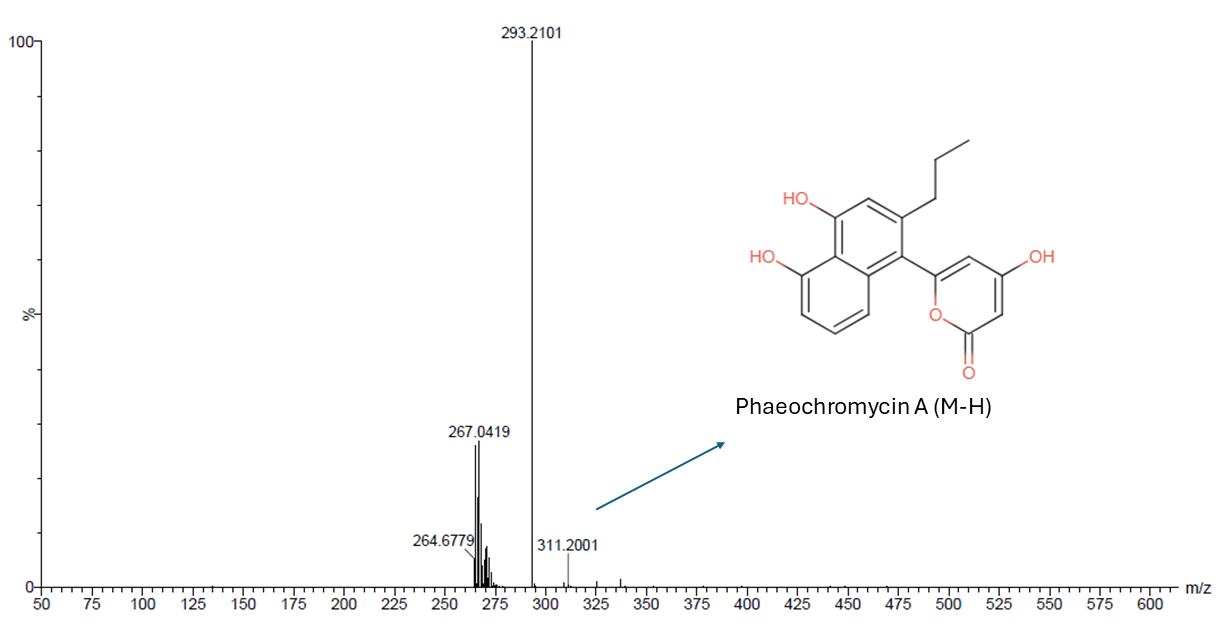
**

Figure 54S: LCMS spectra of Phaeochromycin.

**
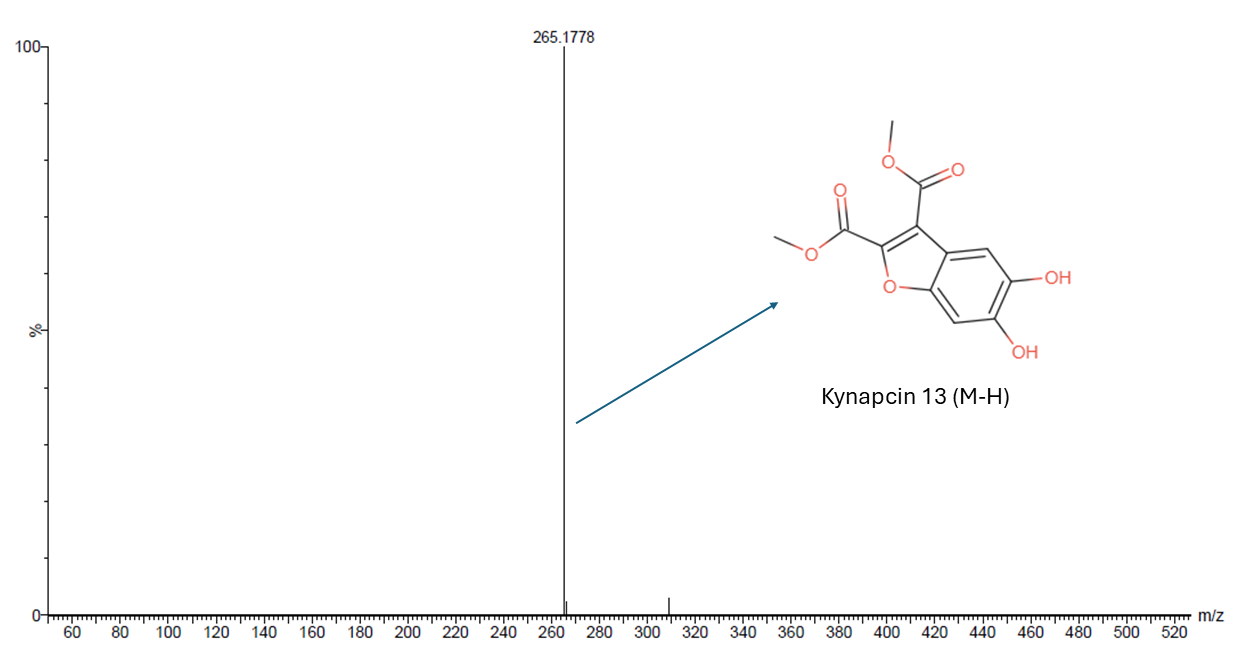
**

Figure 55S: LCMS spectra of kynapcin.

**
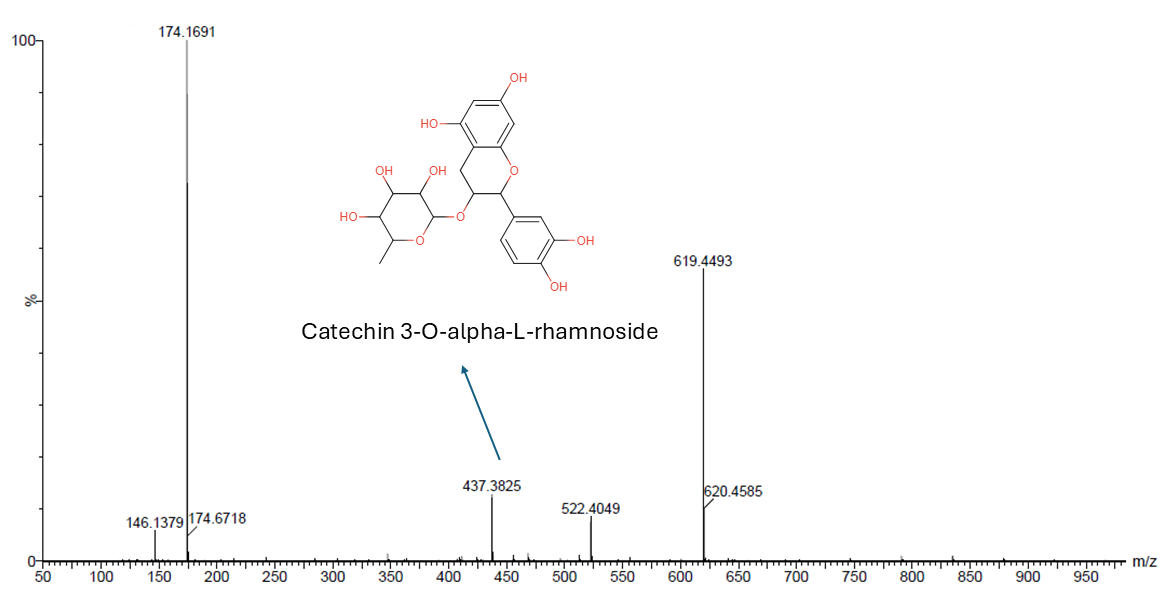
**

Figure 56S: LCMS spectra of catechin 3-Oalpha-L-rhamnoside.


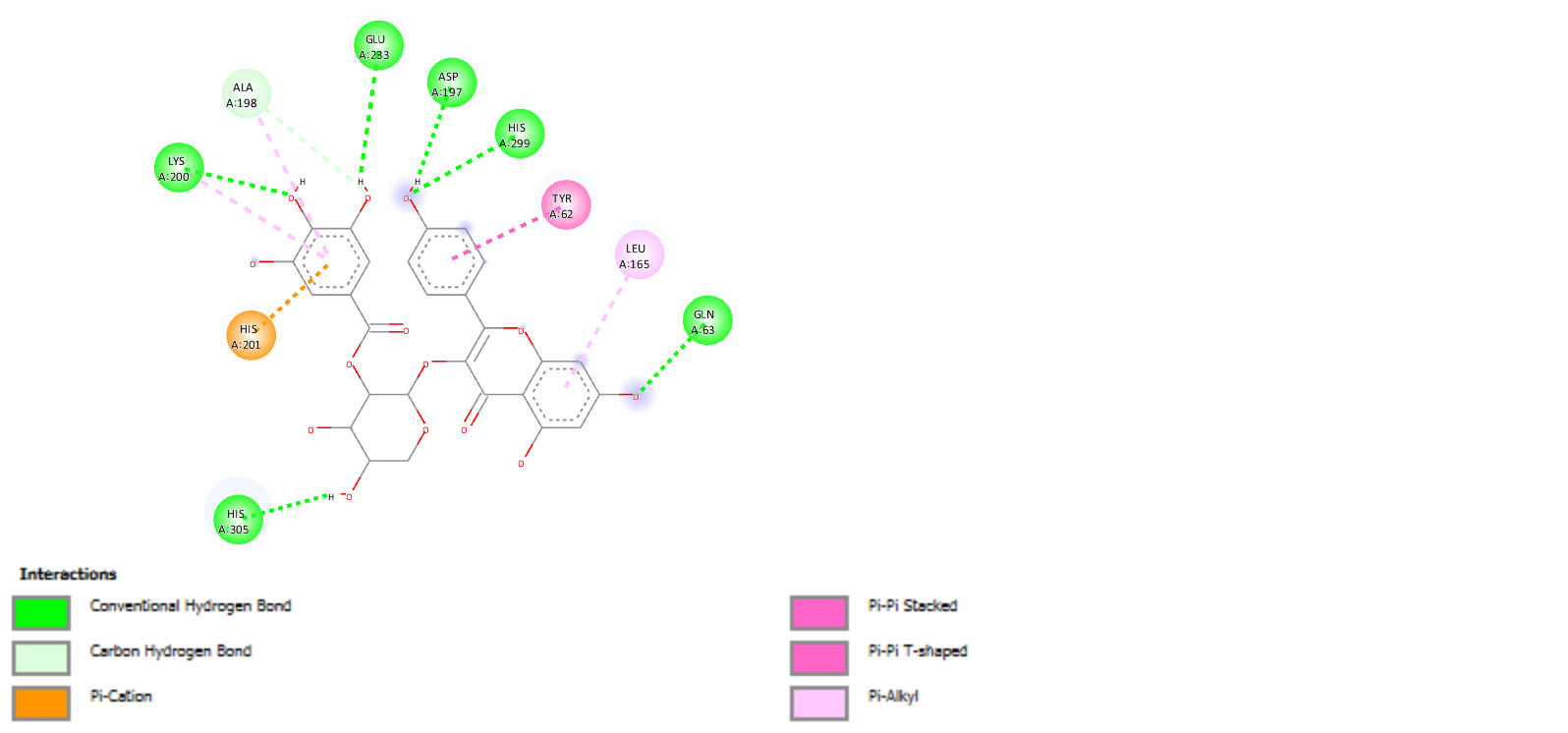


FIGURE 57S: 2D interaction of Kaempferol 3-(2’’-galloyl-α-l-arabinopyranoside) against α-amylase.


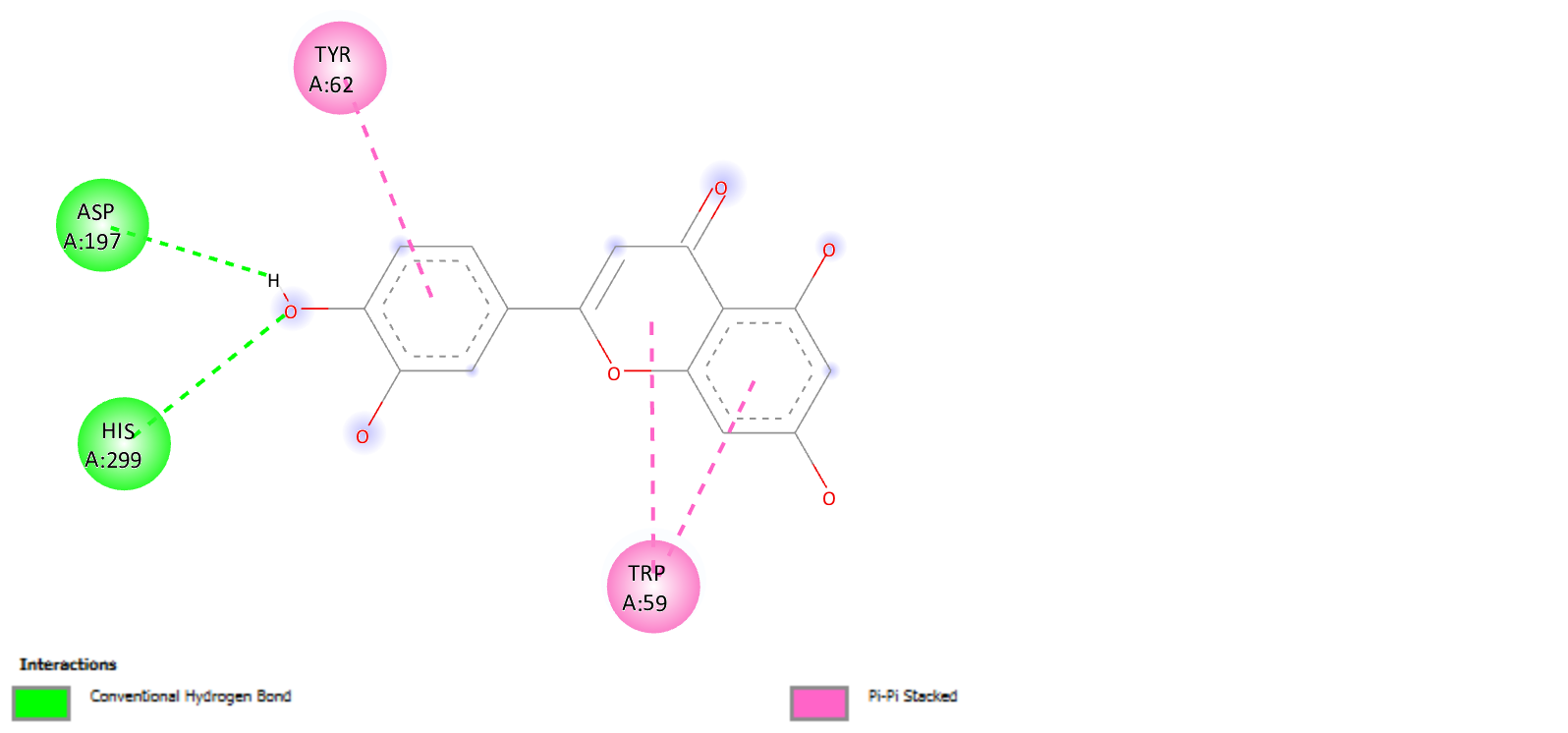


FIGURE 58S: 2D interaction of Luteolin against α-amylase.


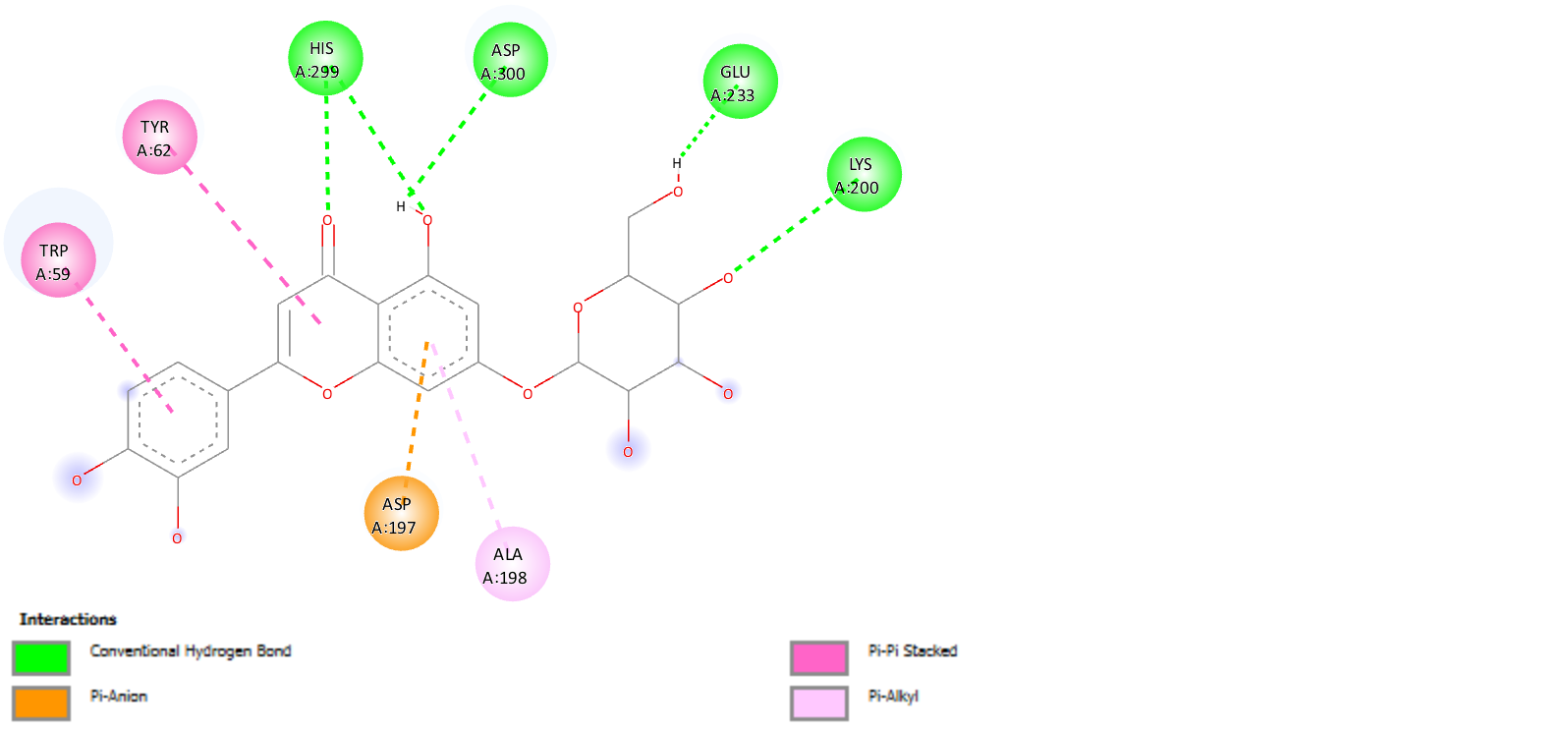


FIGURE 59S: 2D interaction of Luteolin-7-glycoside against α-amylase.


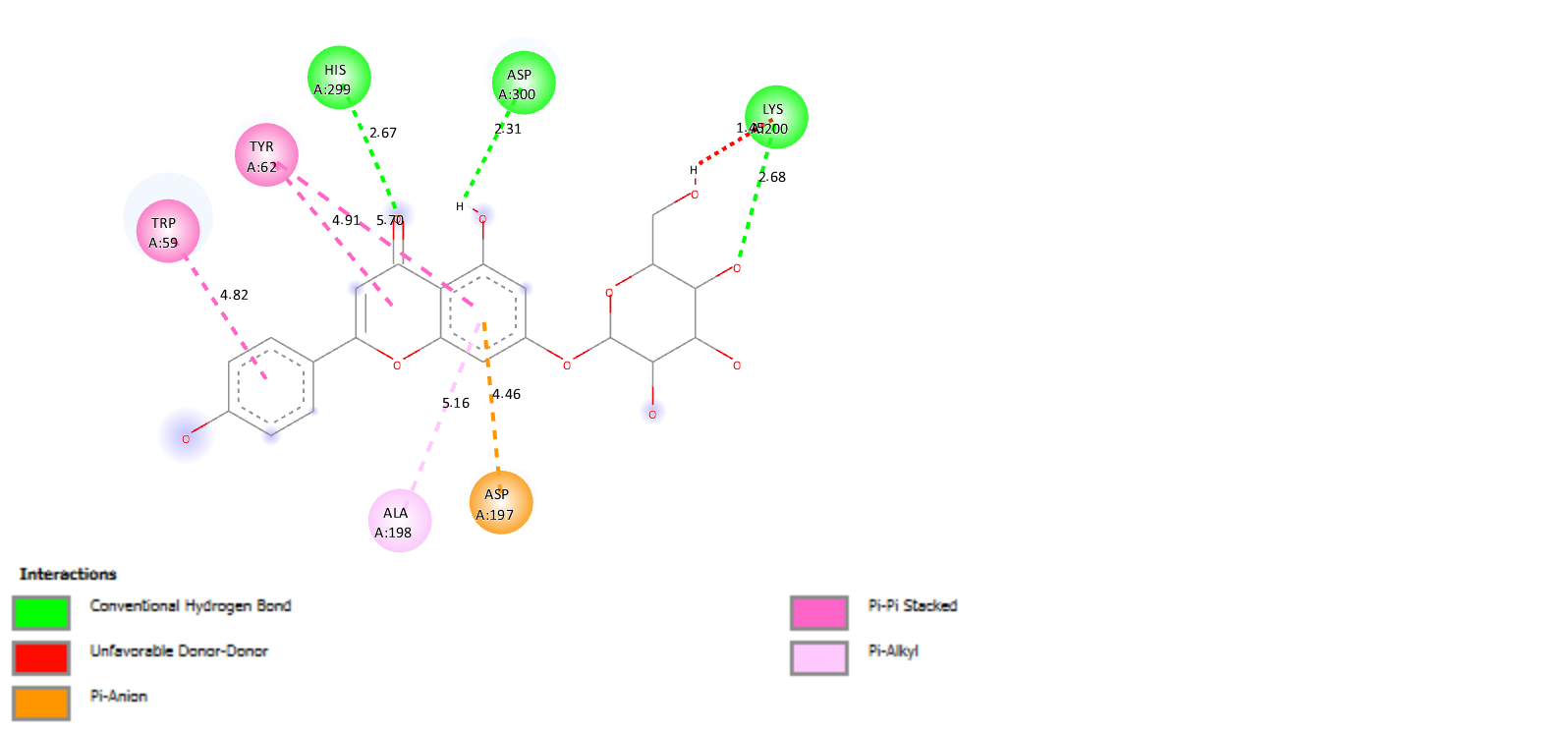


FIGURE 60S: 2D interaction of Apigetrin against α-amylase.

**
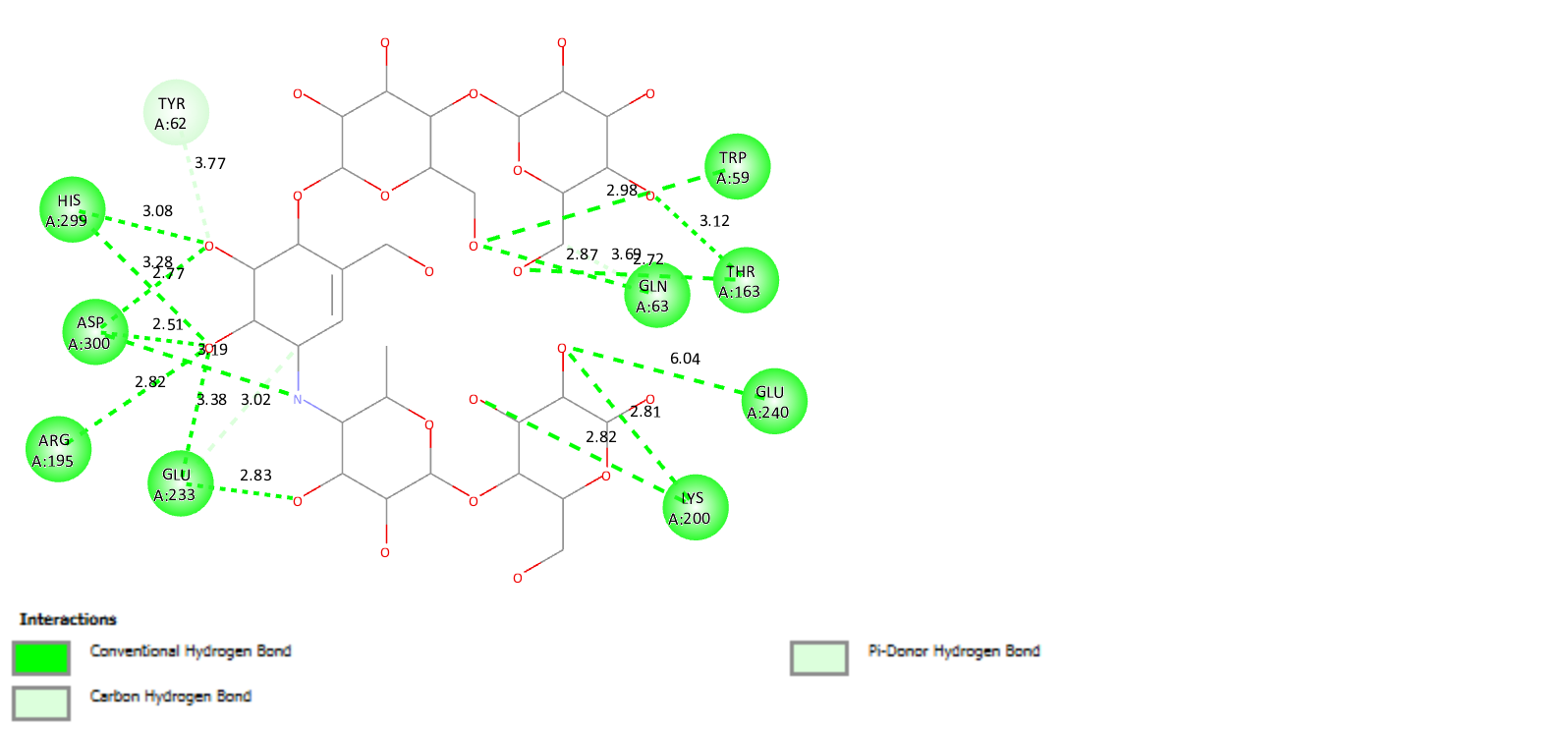
**

FIGURE 61S: 2D interaction of co-crystal ligand acarbose against α-amylase enzyme (PDBID: 3BAJ).
